# Dietary Habits and Age–Health Gradient Among Older Adults in a Region of Japan

**DOI:** 10.3390/nu18050846

**Published:** 2026-03-05

**Authors:** Makoto Hazama, Hiroyo Kagami-Katsuyama, Naohito Ito, Tairo Ogura, Mari Maeda-Yamamoto, Jun Nishihira

**Affiliations:** 1 Department of Medical Management and Informatics, Hokkaido Information University, Ebetsu 069-8585, Japan; m-hazama@do-johodai.ac.jp (M.H.); k.katsuyama@do-johodai.ac.jp (H.K.-K.); n_ito@do-johodai.ac.jp (N.I.); 2 Analytical & Measuring Instruments Division, Shimadzu Corporation, Kyoto 604-8511, Japan; ogutairo@shimadzu.co.jp; 3 Institute of Food Research, National Agriculture and Food Research Organization, Tsukuba 305-8642, Japan; yamamoto.mari733@naro.go.jp

**Keywords:** healthy aging, observational cohort study, ordinal latent block model

## Abstract

**Background/Objectives**: With increasing life expectancy, interest in healthy aging has grown substantially. Dietary habits are among the key factors that contribute to achieving healthy aging. This study analyzes the relationship between dietary habits and the age–health association in older adults, using the first two years of data from an ongoing annual cohort study conducted in a region of Japan. **Methods**: We used observational data from approximately 1200 community-dwelling males and females aged 55 to 75 at baseline, drawing on the first two years of a ten-year annual cohort study conducted from 2023 to 2032. First, dietary habits were classified using an ordinal latent block model (OLBM), a model-based clustering approach applied to food frequency questionnaire (FFQ) data. We then examined whether the age–health gradient—measured across 33 indicators—differed significantly across the derived dietary habit types, using random effects models. **Results**: Dietary habits in the analyzed sample were categorized into six distinct types. Parameter estimates from the model suggest that the extracted patterns represent a continuum ranging from low to high dietary diversity. Regression analyses indicated that, in females, a negative association between age and LDL-C levels was observed among those with highly diverse dietary habits. **Conclusions**: The data-driven classification of dietary habits based on FFQ responses highlights the potential importance of dietary diversity.

## 1. Introduction

With the advent of an aging society driven by historically unprecedented increases in average life expectancy, achieving healthy aging has become a critical challenge [1]. Healthy aging is defined as “the process of developing and maintaining the functional ability that enables well-being in older age” [2]. In older adults, the risks of chronic conditions—including cardiovascular disease, type 2 diabetes, dementia such as Alzheimer’s disease, cancers (particularly colorectal, lung, breast, and prostate cancer), arthritis, chronic respiratory diseases, and frailty or sarcopenia—are higher than in other age groups [3,4].

Strategies to promote healthy aging include modifiable lifestyle factors such as dietary habits, physical activity, and alcohol and tobacco use [5,6]. Among these factors, dietary habits are expected to contribute to the prevention and mitigation of lifestyle-related diseases, the delay in functional decline, and the avoidance of malnutrition and frailty. Consequently, numerous studies have investigated the role of diet in healthy aging [7,8,9,10,11,12,13,14,15,16,17].

The aim of this study is to analyze the relationship between dietary habits and the age–health gradient among community-dwelling elderly adults, using the first two years of data from an ongoing annual cohort survey conducted in a region of Japan. In light of the fact that healthy aging is closely linked to “well-being,” the present analysis examines a wide range of health indicators, including various blood test parameters, physical function, and cognitive function.

A distinctive feature of this study is its approach to dietary habits: rather than relying on previously established dietary patterns or indices commonly used in earlier research, it employs a model-based clustering method to classify types of dietary habits. In the real world, dietary habits exhibit considerable diversity and multidimensionality, and they may not be easily ordered along a single axis with “healthy diet” and “unhealthy diet” as its two extremes. By applying model-based clustering methods, it becomes possible to reduce the dimensionality of dietary information without relying on prior assumptions or preconceived classifications. Moreover, as described in Section 2.2, the model-based clustering method employed in this analysis is so well suited to the ordinal nature of the FFQ data that it effectively precludes the use of other clustering approaches. By estimating and extracting previously unknown dietary habit types within the study sample based on the model and then examining their associations with health status, the study attempts a data-driven analysis.

## 2. Materials and Methods

### 2.1. Dataset Overview

This study uses data from a cohort survey—the Ebetsu Active Future Study (EAFS)—conducted with approximately 1200 males and females living in Ebetsu City, Hokkaido, to examine how the relationships between various health indicators and aging differ depending on dietary habits among older adults. The cohort survey is an observational study scheduled to be conducted annually over a ten-year period from 2023 to 2032; however, the present study analyzes data from the first two survey waves conducted in 2023 and 2024. The survey collects a wide range of information, including physical and mental health status such as cognitive and motor functions, as well as lifestyle factors such as dietary and exercise habits. Participants were males and females aged 55 to 75 years as of 2023, and exclusion criteria required, among other conditions, that individuals be capable of performing basic activities of daily living independently and that they had not been diagnosed with dementia, limb paralysis, major depressive disorder, or neurodegenerative diseases. This study was approved by the Ethics Committee of the Hokkaido Information University (approval date: 2 August 2023; approval number: 2023-04), and written consent was obtained from the participants. The research was conducted in accordance with the Helsinki Declaration. For further details on EAFS, please refer to [18].

**Figure 1:**
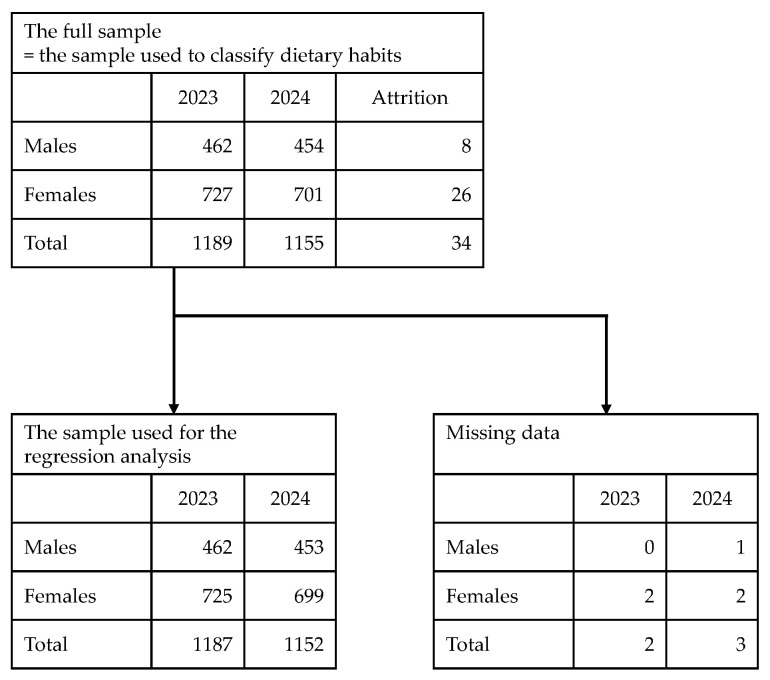
The relationships among the samples employed in each analysis.

From the two waves of EAFS data, we use items from the Food Frequency Questionnaire (FFQ) to capture dietary habits [19]; blood test parameters, cognitive function test scores [20,21], body composition measures, and physical function indicators as measures of health status; and information such as gender, educational attainment, alcohol consumption, and smoking status to control for background factors. Among the analytical methods described in the following subsections, 2344 observations (462 males and 727 females in 2023 and 454 males and 701 females in 2024) are used for the analysis of dietary habit classification, and 2339 observations (462 males and 725 females in 2023 and 453 males and 699 females in 2024) are used for the analysis of the relationships among health status, age, and dietary habits. The attrition rate over the two survey waves is approximately 2.9% overall, and the proportion of missing responses for the items required in the regression analysis is about 0.2% in total (Figure 1).

### 2.2. Dietary Typology Using the Ordinal Latent Block Model

In examining the relationship between dietary habits and age–health gradient, a central issue concerns how dietary habits are represented as variables. Typically, data on dietary habits consist of the frequency and quantity of intake for individual food items. However, owing to both the diversity of foods themselves and the variety of their combinations, dietary habits are inherently high-dimensional. Consequently, to investigate their relationship with health status, it is necessary to typologize diverse dietary habits as a means of dimensionality reduction in data analysis.

Principal component analysis is widely recognized as a representative technique for dimensionality reduction; however, because the data employed in this study on dietary habits are not continuous variables but rather ordinal discrete measures, principal component analysis is not applicable. The FFQ data used in this study include responses on the intake frequency of a total of 144 food items, measured on a nine-point scale (1 = less than once per month, 2 = one to three times per month, 3 = one to two times per week, 4 = three to four times per week, 5 = five to six times per week, 6 = once per day, 7 = two to three times per day, 8 = four to six times per day, 9 = seven or more times per day). Since the data are ordinal discrete variables, this study employs the ordinal latent block model (OLBM) [22], one of the model-based clustering methods, to estimate clusters of dietary habits.

The OLBM, applied to the nine-level intake frequency data of 144 food items from a total of 2344 observations (corresponding to approximately 1200 individuals measured at two time points), simultaneously clusters both the person-times and the food items. The clusters of the 2344 person-times represent typologies of dietary habits, while the clusters of the 144 food items represent groups of foods whose intake frequency patterns are mutually similar within the sample. In the OLBM, for each of the nine intake frequency categories, a continuous latent variable is assumed to follow a normal distribution characterized by location and scale parameters, with values determined by the combination of dietary habit clusters and food clusters. Specifically, the frequency with which an individual belonging to dietary habit cluster q consumes a food belonging to food cluster l is modeled using an ordered probit framework based on a latent variable that follows a normal distribution with mean $\mu_{q,\;l}$ and standard deviation $\sigma_{q,\;l}$. Accordingly, given the specified number of dietary habit clusters and food clusters, and subject to certain identification restrictions, the OLBM allows the classification maximum likelihood estimation of the cluster membership of individuals and food items. The numbers of dietary habit clusters and food clusters can be determined using a greedy search algorithm based on the integrated classification likelihood criterion [22]. In the OLBM, clustering is conducted separately for dietary habits and food items; however, in what follows, the terms “dietary habit cluster” and “dietary habit type” are used interchangeably, particularly with respect to dietary habits. The former reflects terminology grounded in the methodological framework, whereas the latter follows the usage appropriate to the thematic context of this study.

In general, when classifying samples using model-based clustering methods, one advantage is that probabilistically grounded inference becomes possible and the assumptions imposed by the model are explicitly stated. However, it has been pointed out that the obtained results need to be examined in light of external evidence, including domain knowledge [23]. In this study, by taking advantage of the fact that the data constitute a two-wave panel, we are able to construct a transition matrix of dietary habit types derived from the OLBM. This transition matrix allows us to evaluate the stability of each dietary habit type as well as the proximity between types.

### 2.3. Analysis of the Association Between Diet and Health Status

Using the results of typologizing dietary habits by the aforementioned method, this study investigates how aging-related effects on health status and physical function decline are associated with dietary habits among elderly adults. To this end, we estimate the regression model expressed by the following equation:

(1) $$y_{it}=\sum_{q}\left\{\left(\beta_{q}\times 1\left[{Age}_{it}<65\right]+\gamma_{q}\times 1\left[{Age}_{it}\geq 65\right]\right)\times D_{it}^{\left(q\right)}\right\}+x_{it}^{\prime }\delta +u_{i}+\varepsilon_{it}$$

In Equation (1), the subscripts i and t denote the individual $\left(i=1,\;\ldots ,\;n\right)$ and the survey year $\left(t=2023,\;2024\right)$, respectively. The explanatory variable $D_{it}^{\left(q\right)}$ is a dummy variable indicating that individual i at survey time t belongs to Dietary Habit Type q. The explanatory variable ${Age}_{it}$ represents the age of individual i at survey year t. In particular, this study focused on whether the difference in health status between age groups depends on dietary habits, and, hence, incorporated the interaction terms between cluster-specific dummies for dietary habits and age-group dummies into the explanatory variables. Accordingly, the coefficients $\beta_{q}$ and $\gamma_{q}$ capture, respectively, the expected values of the dependent variable $y_{it}$ for individuals under 65 and those aged 65 or older within Dietary Habit Type q.

As dependent variables $y_{it}$ representing health status, we employed a total of 33 items: five related to body composition, two related to physical function, twenty-one derived from blood tests, three pertaining to cognitive function, and blood pressure and heart rate. In each regression model for the respective dependent variables, the explanatory variables other than dietary habits and age included educational attainment, living alone, having experienced any major life changes in the past year, employment status, financial leeway, drinking habits, smoking habits, subjective health status (4-point scale), light exercise habits, moderate-to-vigorous exercise habits, and whether the respondent had hypertension, diabetes, hyperlipidemia, heart or kidney disease, or other illnesses, as well as the survey year. In the observational study, it is possible that, due to a self-selection mechanism, individuals who participate at older ages are relatively more likely to be healthy compared with others in the same age population. For this reason, the regression analyses control for a wide range of confounding factors, including subjective health status. The variables used in the analysis and their summary statistics for the analyzed sample are presented in Table A1 and Table A2 of Appendix A. In Equation (1), the terms $u_{i}$ and $\varepsilon_{it}$ represent the unobserved heterogeneity and idiosyncratic disturbances, respectively.

To examine the relationship between health status and age conditional on dietary habit type, we conducted post hoc multiple testing for the 15 pairwise null hypotheses $H_{0}:\gamma_{q}-\beta_{q}=\gamma_{q^{\prime }}-\beta_{q^{\prime }}$ among the set of regression coefficients $\beta_{q}$ and $\gamma_{q}$ in Equation (1), controlling the false discovery rate (FDR) at 5% [24,25]. While we controlled the FDR when making comparisons across dietary habit types within a single regression model, we did not apply the usual adjustment for multiplicity across the regression models for different dependent variables. This is because the set of dependent variables in our analysis encompasses a wide range of indicators of individual health status, and although the composite health profile captured by their combination is itself important, our primary aim in the initial stage of the analysis was to comprehensively identify which indicators might be associated with dietary habits without overlooking any potential relationships. In other words, our “multiple endpoints” did not constitute hierarchical “primary and secondary families” (e.g., [26]).

In this analysis, we employed a random effects estimation rather than a fixed effects estimation when estimating the regression model in Equation (1). The reasons are as follows. First, because the dataset consists of panel data with only two time points, fixed effects estimation becomes equivalent to the first-differencing method. As a result, the value of $\gamma_{q}-\beta_{q}$ would reflect a comparison not across age groups, but rather between the cohort that was 64 years old in the first survey year and all other cohorts. The same issue would arise even if age were used as a continuous explanatory variable rather than being grouped into categories. In that case, $\gamma_{q}-\beta_{q}$ would simply capture the survey time effect—that is, the difference between the second and first survey years. Second, the main variable of interest, Dietary Habit Type $D_{it}^{\left(q\right)}$, may not exhibit sufficiently large variation in the longitudinal dimension to ensure precise estimation of the coefficient parameters. In such situations, random effects estimation becomes a strong alternative [27]. For these reasons, we adopted random effects estimation instead of fixed effects estimation. To minimize the possibility that unobserved heterogeneity is correlated with the explanatory variables, we included a wide range of survey items as covariates (Table A2). The regression analyses were conducted separately for males and females.

For the analyses in this paper, we used the R application (version 4.5.1) on Windows for the dietary habit classification, and the Python programming language (version 3.11.5) on Windows for the other data processing tasks.

## 3. Results

### 3.1. Typology of Dietary Habits and Food Items

**Figure 2:**
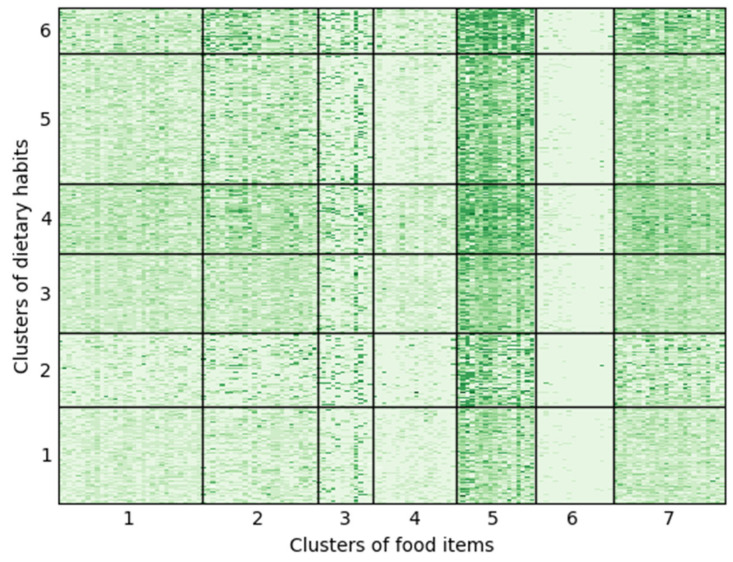
Results of the clustering of food items and dietary habits using the ordinal latent block model (OLBM).

**Figure A1:**
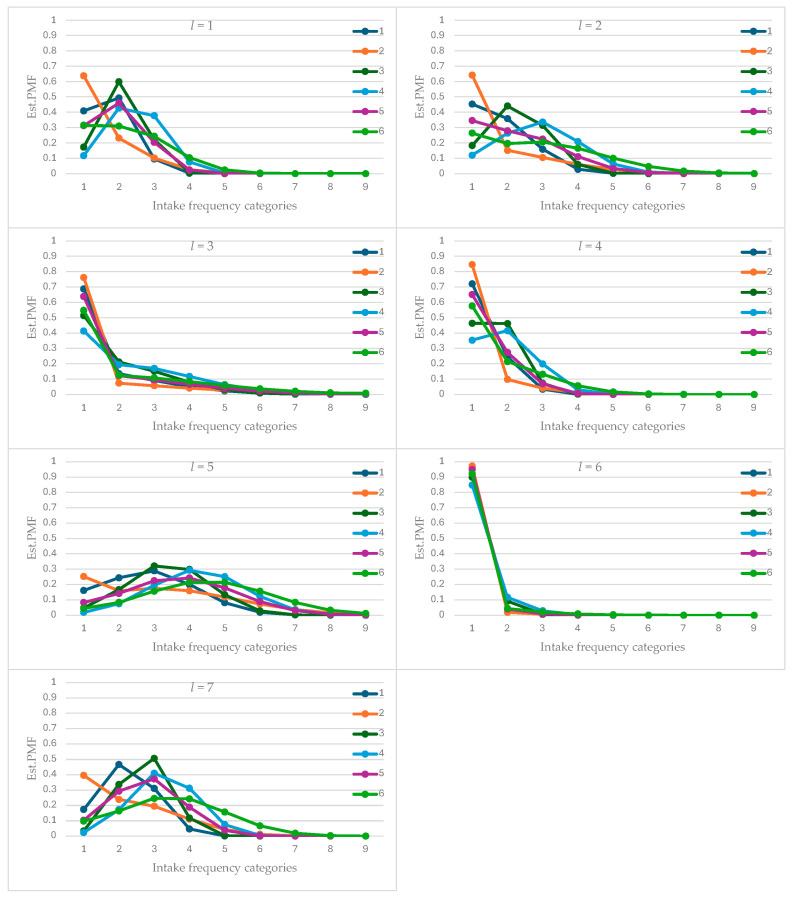
Estimated probability mass functions of stratified food-intake frequency levels for each combination of Food Item Cluster l=1,…,7 and Dietary Habit Cluster q=1, …, 6. The horizontal axes represent the levels of intake frequency (1 = less than once per month, 2 = one to three times per month, 3 = one to two times per week, 4 = three to four times per week, 5 = five to six times per week, 6 = once per day, 7 = two to three times per day, 8 = four to six times per day, 9 = seven or more times per day).

Figure 2 presents the results of clustering dietary habits and food items using the OLBM. On the horizontal axis of the heat map, 144 food items are arranged according to their assigned clusters, while on the vertical axis, a total of 2344 intake-frequency response records (1189 from 2023 and 1155 from 2024) are ordered by dietary habit clusters. According to the integrated classification likelihood criterion, the selected model consists of Q = 6 dietary habit clusters and L = 7 food item clusters (see Supplementary Material, Figure S1). The scale of the heat map corresponds to the nine-level intake-frequency categories described in Section 2.2. Lists of the food items for the seven food item clusters are presented in Table A3 and Table A4 of Appendix B. In the Supplementary Material, Table S1 reports the estimated means and standard deviations of the latent variables for each combination of dietary habit cluster and food item cluster, and Figure A1 of Appendix C presents the estimated probability mass functions for the nine-level categories of food-intake frequency.

Among the seven food item clusters, Food Clusters 5 and 6 exhibit contrasting characteristics (Figure 2 and Figure A1). Foods in Cluster 5 are consumed relatively frequently across all dietary habit clusters and consist of 17 items, including eggs, cheese, yogurt, bread, and natto (fermented soybeans), as well as seven vegetable items such as carrots, cabbage, and tomatoes, and three fruit items—mandarins, apples, and bananas (Table A3 and Table A4). In contrast, foods in Cluster 6 are consumed relatively infrequently across all dietary habit clusters and comprise 17 items that are not typically eaten on a daily basis by the average Japanese person, such as beef (steak), pork (liver), sea bream, eel, papaya, and mango. Based on the shape of the estimated probability mass function of intake frequency levels (Figure A1), along an axis with Clusters 5 and 6 at opposite ends, Food Clusters 7 (24 items including nine vegetables, three mushrooms, and three soybean products), 2 (25 items including seven fruits, four vegetables, three pickles, and four confectioneries), and 1 (31 items including eight meat items categorized by cooking method or cut, eight seafood items, and three tubers) are positioned closer to Cluster 5. Meanwhile, Food Clusters 3 (12 items including regular milk, low-fat milk, and three pickled items) and 4 (18 items including seven seafood items and three meat items categorized by cooking method or cut) are located nearer to Cluster 6. However, food groups with intermediate overall intake frequencies show relatively large variation in intake frequency across dietary habit clusters.

Among the six dietary habit clusters, Clusters 2 and 4 (or 6) exhibit contrasting characteristics (Figure 2 and Figure A1). Dietary Habit Cluster 2 shows a tendency toward relatively low intake frequencies for all food items. In contrast, Clusters 4 (or 6) tend to have relatively high intake frequencies across all food items. Based on the shape of the estimated probability mass function of intake frequency levels (Figure A1), along an axis with Cluster 2 at one extreme and Clusters 4 and 6 at the other, Clusters 1, 3, and 5 occupy intermediate positions between the two extremes. However, the degree to which these differences among dietary habit clusters arise varies by food item cluster, and appears to be particularly large for Food Item Clusters 1, 2, 4, and 7.

As a post hoc analysis, we compared the distribution of the number of food items consumed at each intake frequency across dietary habit clusters (Figure S2 in the Supplementary Materials). Dietary Habit Clusters 4 and 6 showed distributions that were significantly shifted toward higher intake frequencies. This indicates that Clusters 4 and 6 represent dietary patterns with greater dietary diversity than the other clusters. Furthermore, when comparing Clusters 4 and 6, Cluster 6 exhibited a larger variance in the number of food items across intake frequencies. In other words, although both clusters are characterized by high intake frequencies for many food items, the latter shows greater unevenness in these frequencies.

**Figure 3:**
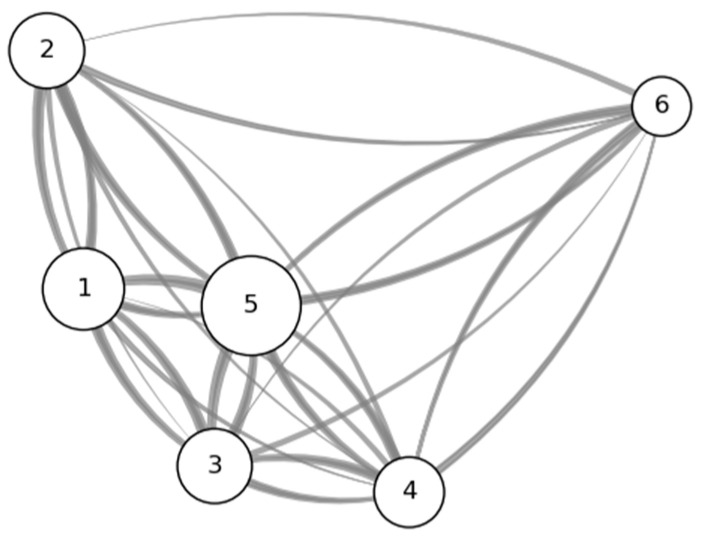
Movement among dietary habit clusters. Note: The node number represents the dietary habit type. The size of each node is proportional to the subsample size in 2023. The thickness of each link is proportional to the number of people who moved between 2023 and 2024. The visualization is created using the Python “network” package, with nodes arranged by the spectral layout.

As an external evaluation of the results from the model-based clustering, Table 1 presents the movements between dietary habit clusters across the two time points in the data period. The proportion of individuals who remained in the same dietary habit cluster across the two time points is 50–60%. Movements among Dietary Habit Types 1, 3, and 5 are relatively large, whereas movements between Types 1 and 4, 1 and 6, 2 and 3, 2 and 4, 2 and 6, and 3 and 6 are comparatively small (Table 1 and Figure 3). The limited movement between Dietary Habit Clusters 2 and 4, as well as between Clusters 2 and 6, supports the pronounced differences between these clusters shown in the results of the estimated probability mass function by dietary habit cluster and food cluster (Figure A1).

### 3.2. Association Between Age-Related Health Deterioration and Dietary Habits

**Figure 4:**
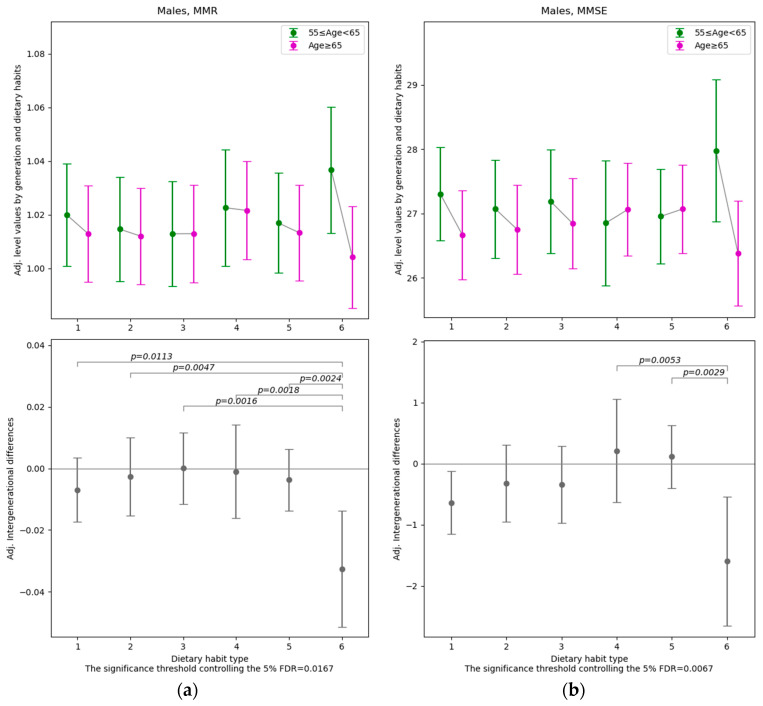
The estimated intergenerational differences in health indicators specific to each Dietary Habit Type $\beta_{q}$ (Age < 65) and $\gamma_{q}$ (Age ≥ 65) in Equation (1) for males. Note: The point estimates and 95% confidence intervals of the coefficients for each dietary habit type. The significance thresholds shown at the bottom of each graph correspond to a multiple-testing procedure that controls the false discovery rate (FDR) at 5% for the 15 pairwise null hypotheses comparing all coefficient pairs: $H_{0}:\gamma_{q}-\beta_{q}=\gamma_{q^{\prime }}-\beta_{q^{\prime }}\;\left(q,\;q^{\prime }\in \left\{1,\;2,\;\ldots ,\;6\right\};q<q^{\prime }\right)$. For the pairs in which the null hypothesis is rejected, the individual *p*-values for each comparison are reported. (**a**) Muscle mass ratio (MMR), (**b**) MMSE total score.

**Figure 5:**
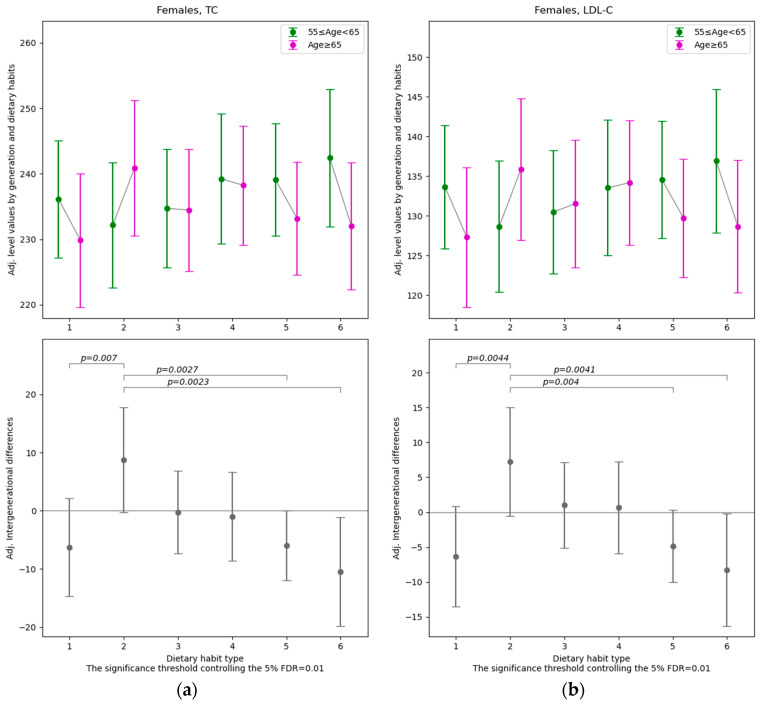
The estimated intergenerational differences in health indicators specific to each Dietary Habit Type $\beta_{q}$ (Age < 65) and $\gamma_{q}$ (Age ≥ 65) in Equation (1) for Females. Note: The point estimates and 95% confidence intervals of the coefficients for each dietary habit type. The significance thresholds shown at the bottom of each graph correspond to a multiple-testing procedure that controls the false discovery rate (FDR) at 5% for the 15 pairwise null hypotheses comparing all coefficient pairs: $H_{0}:\gamma_{q}-\beta_{q}=\gamma_{q^{\prime }}-\beta_{q^{\prime }}\;\left(q,\;q^{\prime }\in \left\{1,\;2,\;\ldots ,\;6\right\};q<q^{\prime }\right)$. For the pairs in which the null hypothesis is rejected, the individual *p*-values for each comparison are reported. (**a**) Total cholesterol (TC), (**b**) LDL-C.

Among the 33 dependent variables related to health status (Table A1), after controlling for gender, educational attainment, employment status, alcohol consumption, smoking, subjective health status, exercise habits, diseases, ApoE4 genotypes and survey year, differences across age groups were found to vary significantly depending on dietary habits for four variables (muscle mass ratio and MMSE total score for males, total cholesterol and LDL-C for females). The results with FDR controlled at 5% are presented in Figure 4 and Figure 5, while the full set of results (including those at other significance levels) is provided in Table S2 of the Supplementary Material.

Among males, the muscle mass ratio (MMR) for Dietary Habit Type 6 exhibits a significantly different age-related gap—between those aged 65 and over and those under 65—compared with any other dietary habit type (the threshold *p*-value for 5% FDR = 0.0167). In this group, the expected value of the adjusted MMR is relatively high among individuals under 65 and relatively low among those aged 65 and over, resulting in a comparatively large negative difference across the two age groups. Furthermore, among males, the MMSE total score for Dietary Habit Type 6 differs significantly between those aged 65 and over and those under 65 when compared with Types 4 and 5 (the threshold *p*-value for 5% FDR = 0.0067). Here again, a pattern similar to that observed for MMR is evident.

Among females, for total cholesterol and LDL-C, the group with Dietary Habit Type 2 shows a significantly different gap between those aged 65 and over and those under 65 compared with Dietary Habit Types 1, 5, and 6 (the threshold *p*-value for 5% FDR = 0.01). In Dietary Habit Type 2, values are higher among those aged 65 and over than among those under 65, whereas in Types 1, 5, and 6, values are lower among those aged 65 and over than among those under 65.

In summary, among males, MMR and the MMSE total score tend to be lower in older age groups within Dietary Habit Type 6. Among females, total cholesterol and LDL-C tend to be higher in older age groups within Dietary Habit Type 2 compared with Types 1, 5, and 6.

The quantitative effects and clinical significance can be assessed by comparing the sample standard deviation (std.) of the dependent variable with the absolute value of the estimated regression coefficients difference $∆\equiv \gamma_{q}-\beta_{q}$. For males with Dietary Habit Type 6, the muscle mass to standard ratio shows (∆, std.) = (−0.033, 0.071), and the MMSE total score shows (∆, std.) = (−1.60, 1.74). These values indicate that the difference between those aged 65 and older and those under 65 can be evaluated as moderate for the muscle mass to standard ratio and considerably large for the MMSE total score. In contrast, for females with Dietary Habit Type 6, total cholesterol and LDL-C show (∆, std.) = (−10.43 mg/dL, 37.00 mg/dL) and (∆, std.) = (−8.24 mg/dL, 31.28 mg/dL), respectively, suggesting that the age-related differences are small to moderate.

## 4. Discussion

The clustering results for dietary habits revealed a bipolar structure, with Dietary Habit Type 2 on one end and Dietary Habit Types 4 and 6 on the other. Dietary Habit Type 2 is characterized by relatively low intake frequencies across a wide range of the 144 food items included in the FFQ. The 144 items do not include rice—the staple food of the Japanese diet—nor alcoholic beverages, granola, and certain other items. Moreover, the consumption of ultra-processed foods and meals eaten outside the home is likely to be underestimated in the FFQ, as these eating occasions do not involve direct handling of ingredients. Thus, Dietary Habit Type 2 encompasses not only a lifestyle in which foods in general are consumed infrequently in a literal sense, but also dietary patterns that are not adequately captured by the FFQ items, as noted above. In contrast, Dietary Habit Types 4 and 6 exhibit relatively high intake frequencies across the FFQ food items. Summarizing the cluster analysis, one dietary habit type characterized by low intake frequencies of the 144 FFQ items (Cluster 2) and two types characterized by high intake frequencies (Clusters 4 and 6) were identified, along with three intermediate dietary habit types (Clusters 1, 3, and 5). It is also noted that the variation in dietary habits over time, as inferred from the transition matrix (Table 1), is moderate for the purposes of the regression analysis.

In the results of the regression analysis, contrasting characteristics were observed between Dietary Habit Type 2 and Dietary Habit Type 6. Specifically, among females, there were significant differences between the Type 2 and Type 6 groups in the age-related differences—those aged 65 years and older versus those under 65 years—in total cholesterol and LDL-C levels. Notably, in the Type 2 group, values tended to be higher in those aged 65 years and older than in those under 65, whereas in the Type 6 group, the opposite pattern was observed, with values tending to be lower in those aged 65 years and older. It is noteworthy that the contrasting patterns identified in the cluster analysis and those identified in the regression analysis both involved Dietary Habit Types 2 and 6. This suggests that differences in the nutritional intake behaviors characteristic of Types 2 and 6 may contribute to differences in health indicators across age groups.

According to previous studies, the trajectories of total cholesterol and LDL-C differ between males and females: in males, total cholesterol and LDL-C increase until their 40s, remain relatively stable until around age 60, and then show a gradual decline thereafter, whereas in females, both markers rise substantially from their 40s to around age 60 and then plateau [28,29]. In females, the postmenopausal decline in estrogen leads to an increase in LDL-C [30]. In the present study, which targeted males and females aged 55 years and older, the results showed that among females, Dietary Habit Type 6 exhibited a negative correlation between age and total cholesterol or LDL-C, whereas Dietary Habit Type 2 showed the opposite pattern. This highlighted the finding that, even within the fundamentally gender-specific differences in lipid metabolic changes, dietary habits are associated with lipid profiles among older females.

For females, it has been noted that lipid profiles are influenced not only by chronological age but also by metabolic phenotype, body composition, and cardiometabolic risk profiles [31,32]. Although addressing such complexity lies beyond the scope of this paper, our findings suggest that dietary habits may also influence lipid profiles among elderly females.

In males, although the contrast between Dietary Habit Type 2 and Type 6 was not observed, the characteristics of Dietary Habit Type 6—specifically, the tendency for those aged 65 and over to have lower values of muscle mass ratio or MMSE total score than those under 65—produced significant differences compared with several other dietary habit types. According to existing studies, both muscle mass [33,34] and cognitive function measured by the MMSE [35,36] are negatively associated with age when limited to the elderly population. However, for both muscle mass ratio and MMSE total score, individuals under 65 in Dietary Habit Type 6 show relatively high levels, while those aged 65 and over exhibit levels comparable to those of the same age group in other dietary habit types. This suggests that Dietary Habit Type 6 may not be associated with age-related decline in body composition or cognitive function among older adults, but rather may be associated with the maintenance or enhancement of body composition or cognitive function among the relatively younger segment within the older population.

The differing regression results for males and females in this study are consistent with previous research reporting sex differences in the relationship between dietary habits and health status [37]. As a possible underlying factor, for example, the negative correlation between age and relative skeletal muscle mass has been shown to be significantly stronger in males than in females [33], which may have contributed to the finding that the age–muscle association was linked to dietary habits only in males. Regarding cognitive function, existing studies have reported that the associations between age and specific cognitive components (e.g., verbal episodic memory, associative memory, short-term memory, etc.) differ between males and females [38]. It is also possible that the influence of dietary habits varies across different components of cognitive function. Moreover, gender differences have been reported in the gene expression patterns that discriminate old from young individuals [39]. In addition, the fact that the accuracy of FFQ data differs between males and females across food groups [40] may also have influenced the gender differences observed in the present analysis. When interpreting the sex-specific results, it is important to consider the potential influence of biological, behavioral, and methodological factors.

This study has several limitations. First, the panel data used in the analysis include only two time points of variation in the longitudinal (time-series) dimension. In this study, we examined the relationship between aging and health indicators conditional on dietary habits, but most of the variation corresponding to aging arises from the cross-sectional dimension. Even though random effects estimation controls for unobserved heterogeneity, the particular concern here is the potential for endogeneity bias stemming from the correlation between cross-sectional age variation and health status. Naturally, to alleviate this endogeneity, we included a broad set of survey variables as covariates, covering a wide range of potential confounders. When data from future cohort studies accumulate, it will become possible to control for unobserved effects that are correlated with respondents’ ages at the baseline survey [27].

Second, this analysis utilizes FFQ data; however, as it is self-reported and relies on memory, it has weaknesses such as recall bias, response bias, social desirability bias, and misclassification ([41] and its references). We did not conduct a validation study of the FFQ because there is no gold-standard measure, such as detailed dietary records, against which it can be validated.

## 5. Conclusions

This study used the first two years of data from a large annual cohort survey of older males and females to classify dietary habits and to examine the hypothesis that differences in health status across age groups vary depending on dietary pattern type. Model-based clustering identified one dietary habit type characterized by generally low intake frequencies across all 144 FFQ food items, two types with high intake frequencies, and three intermediate types. The contrast between the low-frequency and high-frequency dietary types appeared to reflect underlying factors such as dietary diversity, the use of ultra-processed foods and eating out, and the degree of interest in food. Among the findings on diet and health, a particularly notable result for females was that age-related changes in LDL-C were positively associated with the low-frequency dietary type and negatively associated with the high-frequency type. Based on the present analysis, dietary habits appear to be linked to the age–health association among elderly individuals.

## Figures and Tables

**Table 1:** Transition matrix between dietary habit clusters.

	1	2	3	4	5	6	All	1	2	3	4	5	6	All
1	135	24	30	5	21	0	215	62.8%	11.2%	14.0%	2.3%	9.8%	0.0%	100%
2	33	106	1	3	29	9	181	18.2%	58.6%	0.6%	1.7%	16.0%	5.0%	100%
3	31	0	91	20	31	2	175	17.7%	0.0%	52.0%	11.4%	17.7%	1.1%	100%
4	1	1	28	93	24	13	160	0.6%	0.6%	17.5%	58.1%	15.0%	8.1%	100%
5	42	24	40	27	160	21	314	13.4%	7.6%	12.7%	8.6%	51.0%	6.7%	100%
6	0	3	3	20	24	60	110	0.0%	2.7%	2.7%	18.2%	21.8%	54.5%	100%
All	242	158	193	168	289	105	1155	21.0%	13.7%	16.7%	14.5%	25.0%	9.1%	100%

Note: The row labels and column labels represent the dietary habit clusters for 2023 and 2024, respectively. The table on the left shows the raw counts, while the table on the right shows the row-wise composition ratios.

## Data Availability

The datasets presented in this article are not readily available because the data are part of an ongoing study. Requests to access the datasets should be directed to the correspondence author.

## Supplementary Materials

The following supporting information can be downloaded at: https://www.mdpi.com/article/10.3390/nu18050846/s1, Figure S1: The progression of the integrated classification likelihood criterion in the greedy search algorithm; Figure S2. Distribution of intake frequency by dietary habit clusters and gender; Table S1: Estimated parameters of the ordinal latent block model (OLBM); Table S2: Regression results.

## Abbreviations

The following abbreviations are used in this manuscript:

EAFS	Ebetsu Active Future Study
FDR	False discovery rate
FFQ	Food frequency questionnaire
OLBM	Ordinal latent block model

## Appendix A

**Table A1:** Summary statistics for the dependent variable in the regression analysis (n = 2339).

	Mean	S.D.	Min	Max
BMI	22.9	3.34	14.6	37.3
Body fat percentage (%)	27.6	7.60	4.8	53.6
Visceral fat level ^(a)^	8.20	4.27	1	23
Muscle mass to standard ratio ^(b)^	1.00	0.07	0.79	1.39
Leg muscle mass score ^(c)^	93.2	8.41	66	120
Grip strength (kg) ^(d)^	29.7	8.27	12.1	57.7
Sit-to-stand test (seconds) ^(e)^	5.95	1.80	3.1	23.3
Systolic blood pressure (mmHg)	133.1	18.62	83.3	204.7
Heart rate (bpm)	73.7	11.73	38.7	124.7
Total protein (g/dL)	7.41	0.41	6.2	11.8
Albumin (g/dL)	4.40	0.28	2.5	5.4
AST (U/L)	24.8	8.13	11	190
ALT (U/L)	23.0	11.66	5	143
LDH (U/L)	202.1	31.63	102	489
ALP (U/L)	78.1	23.41	23	477
γ-GTP (U/L)	34.8	36.01	9	502
Casual blood glucose (mg/dL)	94.5	17.15	49	432
HbA1c (%)	5.64	0.53	4.6	13.1
BUN (mg/dL)	15.8	3.79	4.9	34.7
Creatinine (mg/dL)	0.80	0.18	0.43	1.9
Uric acid (mg/dL)	4.92	1.19	0.6	10.0
Triglycerides (mg/dL)	128.5	82.10	20	1200
Total cholesterol (mg/dL)	226.0	37.00	72	400
HDL-C (mg/dL)	73.3	19.37	25	166
LDL-C (mg/dL)	126.4	31.28	23	279
Na (mEq/L)	142.0	1.77	132	147
Mg (mg/dL)	2.14	0.16	1.3	2.8
Ca (mg/dL)	9.29	0.35	8.2	11.1
Fe (μg/dL)	100.2	29.60	11	217
High-sensitivity C-reactive protein (mg/dL)	0.137	0.896	0.004	35.99
MMSE total score	28.2	1.74	18	30
MoCA-J total score	25.3	2.87	10	30
Aβ-CM ^(f)^	−0.15	0.72	−3.01	3.62

Note: The sample consists of 1187 individuals from 2023 and 1152 individuals from 2024. (a) Score (1–59 points) according to visceral fat accumulation, categorized as 1–9 (normal), 10–14 (slightly excessive), and ≥15 (excessive). (b) The ratio of muscle mass to the standard muscle mass. (c) Score (50–150 points) based on how the percentage of leg muscle mass to body weight compares with the ideal value. (d) The greater grip strength of the right and left hands. (e) Time required to perform five consecutive chair stands. (f) Amyloid beta composite marker [42].

**Table A2:** Summary statistics for the independent variable in the regression analysis (n = 2339).

	Mean	S.D.	Min	Max	
Age	65.0	5.57	55	76	
Dietary Habit Cluster 1	0.197	0.398	0	1	
Dietary Habit Cluster 2	0.146	0.353	0	1	
Dietary Habit Cluster 3	0.160	0.367	0	1	
Dietary Habit Cluster 4	0.141	0.348	0	1	
Dietary Habit Cluster 5	0.262	0.440	0	1	
Dietary Habit Cluster 6	0.094	0.292	0	1	
Female	0.609	0.488	0	1	
School years ≥ 13	0.582	0.493	0	1	13 or more years of education
Non-cohabitants	0.095	0.293	0	1	Lives alone
Lifestyle change	0.340	0.474	0	1	A major change in lifestyle during the past year
Paid	0.589	0.492	0	1	Engaged in paid work
Well off	0.267	0.443	0	1	The household is financially comfortable
Alcohol	0.543	0.498	0	1	Having a drinking habit
Smoking	0.077	0.267	0	1	Having a smoking habit
Healthy 1	0.110	0.313	0	1	Subjective health status (4-point scale) = 1 (Very)
Healthy 2	0.794	0.405	0	1	Subjective health status (4-point scale) = 2 (Fairly)
Exercise 1	0.814	0.389	0	1	Engaged in light exercise
Exercise 2	0.575	0.495	0	1	Engages in moderate or vigorous exercise
Disease 1	0.212	0.409	0	1	Hypertension
Disease 2	0.063	0.243	0	1	Diabetes
Disease 3	0.162	0.368	0	1	Hyperlipidemia
Disease 4	0.037	0.189	0	1	Heart disease
Disease 5	0.039	0.193	0	1	Kidney disease
Disease 4	0.320	0.467	0	1	Other diseases
ApoE4 hetero	0.223	0.416	0	1	Carriers of the ApoE4 heterozygous genotypes
ApoE4 homo	0.012	0.107	0	1	Carriers of the ApoE4 homozygous genotypes

Note: The sample consists of 1187 individuals from 2023 and 1152 individuals from 2024.

## Appendix B

**Table A3:** Clustering results of food items (clusters 1, 2, and 3).

Cluster	Food	Cluster	Food	Cluster	Food
1	Pork (deep-fried)	2	Fish sausage (chikuwa)	3	Beef (stir-fried)
1	Pork (stewed)	2	Pickled plum	3	Low-fat milk
1	Chicken (broiled)	2	Pickled Chinese cabbage	3	Milk
1	Chicken (stir-fried)	2	Pickled cucumber	3	Pickled radish
1	Chicken (simmered)	2	Green chive	3	Pickled eggplant
1	Chicken (deep-fried)	2	Green vegetable (komatsuna)	3	Pickled turnip
1	Ham	2	Snap bean	3	Scallion
1	Bacon	2	Garlic	3	Butter
1	Dried fish	2	Other oranges	3	Margarine
1	Canned tuna	2	Persimmon	3	Jam
1	Salmon & trout	2	Strawberry	3	Honey
1	Tunas & bonito	2	Grapes	3	Roasted soybean flour
1	Codfish & flatfish	2	Watermelon		
1	Pacific saury & mackerel	2	Pears	cluster	food
1	Shrimp	2	Kiwi fruit	4	Beef (broiled)
1	Boiled fish paste (kamaboko)	2	Biscuit & cookie	4	Beef (stewed)
1	Fried fish paste (satsuma-age)	2	Ice cream	4	Pork (soup)
1	Burdock	2	Snacks	4	Yellowtail amberjack
1	Melon	2	Rice cracker	4	Horse mackerel & sardine
1	Peach	2	Sesame	4	Shirasuboshi
1	Japanese noodles (soba)	2	Peanuts	4	Cod roe & salmon roe
1	Chinese noodles	2	Worcester sauce	4	Squid
1	Pasta	2	Ketchup	4	Octopus
1	Japanese noodles (soumen)	2	Mustard	4	Clam & corb shell
1	Japanese cake	2	Red pepper	4	Pickled green vegetables
1	Deep-fried tofu			4	Green vegetable (shungiku)
1	Sweet potatoes			4	Pak choi
1	Yams			4	Pineapple
1	Konjac			4	Rice cake
1	Seaweed (hijiki)			4	Cakes
1	Wasabi			4	Freeze-dried tofu
				4	Taros

Note: The lists of food items for the 1st to 4th of the seven clusters shown in Figure 1.

**Table A4:** Clustering results of food items (Clusters 5, 6, and 7).

Cluster	Food	Cluster	Food	Cluster	Food
5	Eggs	6	Beef (steak)	7	Pork (stir-fried)
5	Cheeses	6	Pork (simmered)	7	Sausage
5	Yogurt	6	Pork (liver)	7	Salted fish
5	Carrot	6	Chicken (liver)	7	Spinach
5	Cabbage	6	Luncheon meat	7	Pumpkin
5	Tomatoes	6	Sea breams	7	Radish
5	Japanese long onion	6	Eel	7	Green pepper
5	Onion	6	Pond snail	7	Broccoli
5	Cucumber	6	Mustard greens	7	Eggplant
5	Lettuce	6	Bitter melon	7	Chinese cabbage
5	Mandarin	6	Swiss chard	7	Bean sprout
5	Apple	6	Sponge gourd	7	Green asparagus
5	Banana	6	Mugwort	7	Japanese noodles (udon)
5	Bread	6	Papaya	7	Bean curd (in miso soup)
5	Chocolate	6	Mango	7	Bean curd (boiled tofu)
5	Fermented soybeans	6	Okinawa noodles	7	Fried tofu pouch
5	Salad dressings	6	Fresh tofu	7	Potatoes
				7	Mushroom (shiitake)
				7	Mushroom (enokidake)
				7	Mushroom (shimeji)
				7	Seaweed (wakame)
				7	Dried seaweed (nori)
				7	Mayonnaise
				7	Ginger

Note: The lists of food items for the 5th to 7th of the seven clusters shown in Figure 1.
