# Supplementary material for: Dietary Habits and Age–Health Gradient Among Older Adults in a Region of Japan
Source: Nutrients. 2026 Mar 5;18(5):846. doi: 10.3390/nu18050846 (PMC12986981; doi:10.3390/nu18050846)
Supplement: Supplementary file 1 [file nutrients-18-00846-s001.zip › nutrients-4149524-supplementary.pdf]

Supplementary Materials

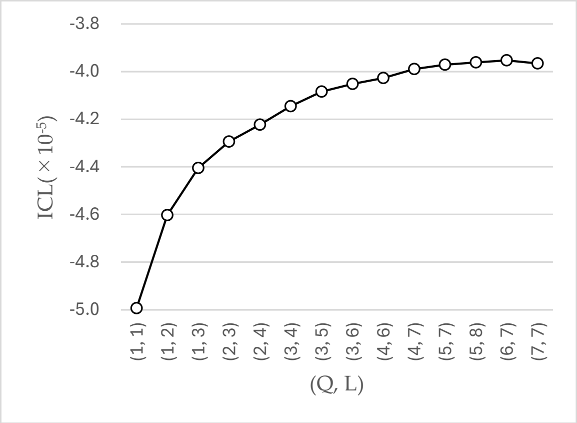

Figure S1. The progression of the integrated classification likelihood criterion in the greedy search algorithm.

Note: Q = the number of dietary-habit clusters; L = the number of food-item clusters.

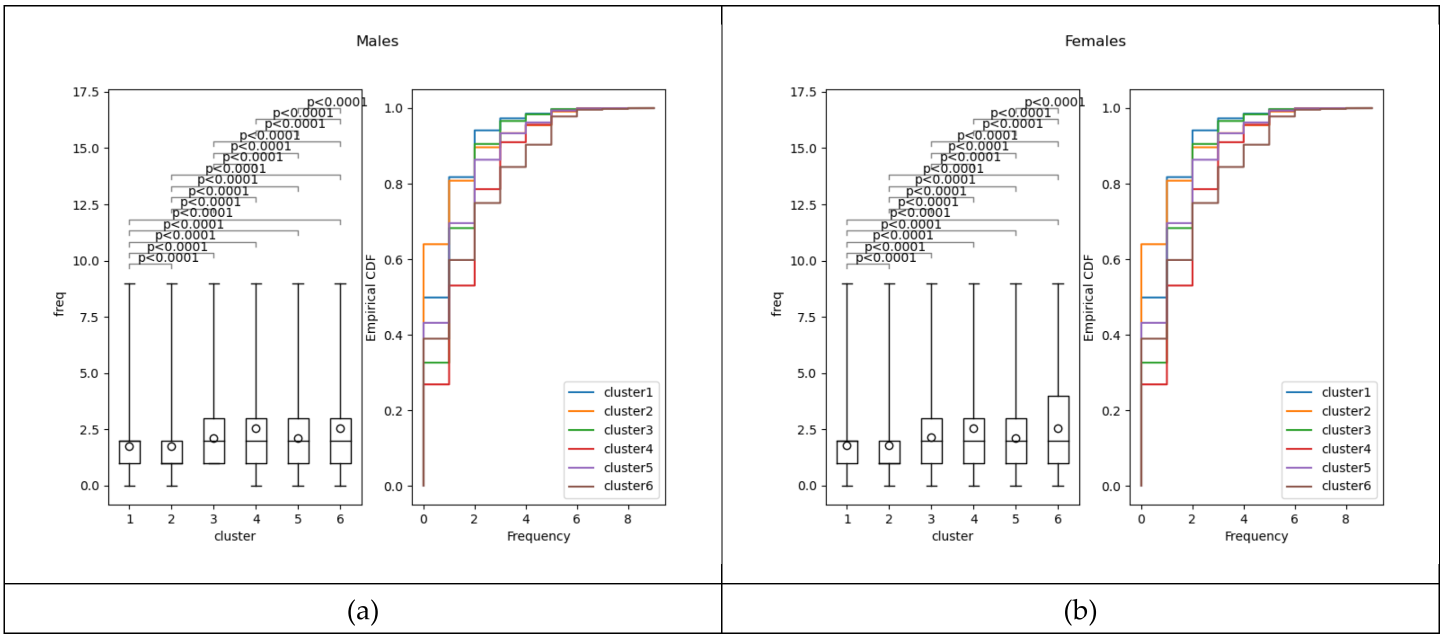

(a) (b)

Figure S2. Distribution of intake frequency by dietary habit clusters and gender

Note: the p-values from the Dwass, Steel, Critchlow and Fligner all-pairs comparison test. (a) Males, (b) Females.

Table S1. Estimated parameters of the ordinal latent block model (OLBM)

$\mu_{q,l}$

	1	2	3	4	5	6	7
1	1.65	1.62	0.39	1.02	2.82	-0.47	2.22
2	1.04	0.70	-1.20	-0.29	3.00	-2.54	1.94
3	2.06	2.24	1.44	1.56	3.40	0.39	2.72
4	2.42	2.84	1.94	1.84	4.23	0.20	3.26
5	1.90	2.06	0.50	1.13	3.70	-0.89	2.76
6	2.10	2.69	1.14	1.19	4.50	-2.00	3.47

$\sigma_{q,l}$

	1	2	3	4	5	6	7
1	0.43	1.00	5.22	0.67	1.78	1.36	0.59
2	1.69	4.82	14.36	3.09	5.06	4.45	2.69
3	0.35	0.67	3.14	0.42	1.29	0.74	0.45
4	0.59	1.30	4.13	0.81	1.71	1.57	0.79
5	0.65	1.96	7.94	0.91	2.51	2.14	1.00
6	1.56	3.56	9.31	2.64	3.11	6.08	2.31

Note: The left and right tables show, respectively, the means and standard deviations of the latent variables for each combination of dietary habit cluster q and food cluster l. In each table, the rows correspond to the dietary habit clusters q, and the columns correspond to the food clusters l.

Table S2. Regression results (BMI)

BMI	Males						Females					
	Coef.	S.E.	t	P(t)	[95%	C.I.]	Coef.	S.E.	t	P(t)	[95%	C.I.]
1[Age<65] × Dietary 1	23.72	0.339	69.97	0.000	23.05	24.38	22.73	0.239	95.18	0.000	22.26	23.20
1[Age<65] × Dietary 2	23.59	0.343	68.76	0.000	22.91	24.26	22.92	0.245	93.42	0.000	22.44	23.41
1[Age<65] × Dietary 3	23.72	0.345	68.83	0.000	23.04	24.39	22.72	0.236	96.37	0.000	22.26	23.19
1[Age<65] × Dietary 4	23.89	0.375	63.79	0.000	23.16	24.63	22.55	0.246	91.82	0.000	22.07	23.03
1[Age<65] × Dietary 5	23.64	0.333	71.05	0.000	22.98	24.29	22.79	0.232	98.28	0.000	22.34	23.25
1[Age<65] × Dietary 6	24.27	0.400	60.64	0.000	23.48	25.05	22.79	0.256	88.89	0.000	22.28	23.29
1[Age≥65] × Dietary 1	23.53	0.324	72.67	0.000	22.90	24.17	22.80	0.252	90.51	0.000	22.30	23.29
1[Age≥65] × Dietary 2	23.65	0.322	73.57	0.000	23.02	24.28	22.94	0.261	87.91	0.000	22.42	23.45
1[Age≥65] × Dietary 3	23.65	0.326	72.46	0.000	23.01	24.29	22.93	0.239	95.88	0.000	22.47	23.40
1[Age≥65] × Dietary 4	23.67	0.329	72.07	0.000	23.03	24.32	22.81	0.238	96.02	0.000	22.34	23.28
1[Age≥65] × Dietary 5	23.67	0.321	73.69	0.000	23.04	24.30	22.80	0.232	98.21	0.000	22.35	23.26
1[Age≥65] × Dietary 6	23.50	0.337	69.67	0.000	22.83	24.16	22.84	0.246	92.91	0.000	22.35	23.32
School	0.362	0.298	1.215	0.225	-0.223	0.947	-0.715	0.236	-3.033	0.003	-1.177	-0.252
Non cohabitants	0.222	0.254	0.876	0.381	-0.276	0.720	-0.250	0.149	-1.676	0.094	-0.543	0.043
Lifestyle change	-0.043	0.060	-0.713	0.476	-0.160	0.075	-0.051	0.045	-1.131	0.258	-0.140	0.038
Paid	-0.025	0.110	-0.228	0.820	-0.242	0.192	-0.198	0.087	-2.270	0.023	-0.368	-0.027
Well off	-0.050	0.093	-0.541	0.588	-0.232	0.131	-0.013	0.065	-0.201	0.841	-0.140	0.114
Alcohol	0.305	0.133	2.294	0.022	0.044	0.566	-0.121	0.082	-1.472	0.141	-0.283	0.040
Smoking	-0.005	0.227	-0.021	0.984	-0.450	0.441	0.134	0.246	0.545	0.586	-0.348	0.616
Healthy 1	0.026	0.150	0.172	0.863	-0.269	0.320	-0.092	0.124	-0.740	0.459	-0.334	0.151
Healthy 2	0.134	0.108	1.244	0.214	-0.078	0.347	-0.152	0.097	-1.561	0.119	-0.343	0.039
Exercise 1	0.064	0.095	0.669	0.504	-0.123	0.250	-0.066	0.072	-0.924	0.356	-0.207	0.075
Exercise 2	-0.205	0.082	-2.489	0.013	-0.367	-0.043	0.073	0.062	1.179	0.239	-0.048	0.194
Disease 1	0.128	0.109	1.174	0.241	-0.086	0.342	0.187	0.102	1.826	0.068	-0.014	0.387
Disease 2	-0.343	0.181	-1.890	0.059	-0.699	0.013	0.417	0.241	1.730	0.084	-0.056	0.890
Disease 3	-0.041	0.126	-0.328	0.743	-0.289	0.206	0.134	0.097	1.383	0.167	-0.056	0.325
Disease 4	-0.013	0.155	-0.082	0.935	-0.317	0.292	0.432	0.284	1.521	0.129	-0.125	0.988
Disease 5	-0.028	0.115	-0.240	0.810	-0.253	0.198	0.340	0.299	1.137	0.256	-0.247	0.926
disease 6	-0.009	0.069	-0.137	0.891	-0.144	0.126	-0.033	0.059	-0.556	0.579	-0.150	0.084
ApoE4 hetero	-0.037	0.319	-0.115	0.909	-0.663	0.590	-0.334	0.294	-1.137	0.256	-0.910	0.242
ApoE4 homo	-2.605	1.719	-1.515	0.130	-5.979	0.770	0.405	0.964	0.420	0.675	-1.487	2.297
Y2024	-0.089	0.033	-2.682	0.008	-0.154	-0.024	0.087	0.027	3.239	0.001	0.034	0.140
	N		915	F		2.856	N		1424	F		5.893
	R2 (Between)		0.015	P(F)		0.000	R2 (Between)		0.053	P(F)		0.000
	R2 (Within)		0.098	σ2(u)		8.276	R2 (Within)		0.047	σ2(u)		9.410
	R2 (Overall)		0.017	σ2(ε)		0.211	R2 (Overall)		0.052	σ2(ε)		0.208
	Log-likelihood		-589.8	σ2(u)/σ2(u+ε)		0.975	Log-likelihood		-920.8	σ2(u)/σ2(u+ε)		0.978

Note: "Dietary" denotes the dietary habit cluster: "Dietary 1" is a dummy variable indicating whether an individual belongs to dietary habit cluster 1, and so on. "School": an individual has 13 or more years of education; "Non cohabitants": the individual lives alone; "Lifestyle change": the individual experienced a major change in lifestyle during the past year; "Paid": the individual is engaged in paid work; "Well off": the household is financially comfortable; "Alcohol": the individual has a drinking habit; "Smoking": the individual has a smoking habit; "Health 1 (or 2)": the subjective health status is "very" (or "fairly") healthy; "Exercise1": the individual engages in light exercise at least once per week; "Exercise2": the individual engages in moderate or vigorous exercise at least once per week; "Y 2024": that the survey year is 2024. "Disease1" through "Disease6" are indicators for diseases under treatment or with lasting effects: "Disease1" is hypertension, "Disease2" is diabetes, "Disease3" is hyperlipidemia, "Disease4" is heat disease, "Disease 5" is kindey disease,, and "Disease 6" is other diseases. "ApoE4 hetero" and "ApoE4 homo" are dummy variables indicating carriers of the ApoE4 heterozygous and homozygous genotypes, respectively.

Table S2 (continued). Regression results (Body fat percentage)

Body fat %	Males						Females					
	Coef.	S.E.	t	P(t)	[95%	C.I.]	Coef.	S.E.	t	P(t)	[95%	C.I.]
1[Age<65] × Dietary 1	23.08	0.766	30.15	0.000	21.57	24.58	32.62	0.577	56.51	0.000	31.49	33.75
1[Age<65] × Dietary 2	23.20	0.780	29.74	0.000	21.67	24.73	32.83	0.597	54.96	0.000	31.66	34.00
1[Age<65] × Dietary 3	23.68	0.790	29.96	0.000	22.13	25.23	32.52	0.569	57.14	0.000	31.41	33.64
1[Age<65] × Dietary 4	24.13	0.891	27.07	0.000	22.38	25.87	32.07	0.599	53.50	0.000	30.89	33.24
1[Age<65] × Dietary 5	23.42	0.750	31.25	0.000	21.95	24.89	32.67	0.557	58.66	0.000	31.58	33.76
1[Age<65] × Dietary 6	23.69	0.976	24.27	0.000	21.77	25.60	32.79	0.631	51.98	0.000	31.55	34.03
1[Age≥65] × Dietary 1	23.38	0.720	32.49	0.000	21.97	24.79	32.49	0.618	52.62	0.000	31.28	33.70
1[Age≥65] × Dietary 2	23.77	0.714	33.30	0.000	22.37	25.17	32.98	0.642	51.37	0.000	31.72	34.24
1[Age≥65] × Dietary 3	23.78	0.729	32.63	0.000	22.35	25.21	32.86	0.580	56.69	0.000	31.72	34.00
1[Age≥65] × Dietary 4	23.23	0.735	31.59	0.000	21.78	24.67	32.68	0.574	56.92	0.000	31.56	33.81
1[Age≥65] × Dietary 5	23.71	0.711	33.34	0.000	22.31	25.11	32.66	0.558	58.58	0.000	31.57	33.75
1[Age≥65] × Dietary 6	23.88	0.769	31.05	0.000	22.37	25.39	32.52	0.599	54.31	0.000	31.34	33.69
School	0.707	0.557	1.271	0.204	-0.385	1.799	-1.442	0.515	-2.800	0.005	-2.453	-0.432
Non cohabitants	0.306	0.654	0.468	0.640	-0.978	1.590	-0.576	0.393	-1.465	0.143	-1.347	0.195
Lifestyle change	0.206	0.168	1.226	0.220	-0.124	0.536	-0.034	0.124	-0.271	0.786	-0.276	0.209
Paid	-0.201	0.296	-0.680	0.497	-0.782	0.379	-0.799	0.232	-3.443	0.001	-1.254	-0.344
Well off	0.036	0.254	0.143	0.886	-0.462	0.535	-0.073	0.175	-0.417	0.677	-0.416	0.270
Alcohol	0.524	0.343	1.529	0.127	-0.148	1.196	-0.330	0.219	-1.504	0.133	-0.760	0.100
Smoking	-0.594	0.558	-1.064	0.288	-1.689	0.502	-0.261	0.639	-0.409	0.683	-1.514	0.992
Healthy 1	-0.557	0.419	-1.329	0.184	-1.381	0.266	-0.371	0.336	-1.105	0.270	-1.030	0.288
Healthy 2	-0.149	0.303	-0.491	0.624	-0.743	0.446	-0.549	0.264	-2.078	0.038	-1.066	-0.031
Exercise 1	-0.053	0.266	-0.200	0.841	-0.576	0.469	-0.180	0.195	-0.920	0.358	-0.563	0.204
Exercise 2	-0.552	0.229	-2.406	0.016	-1.002	-0.102	0.157	0.168	0.936	0.349	-0.172	0.485
Disease 1	0.526	0.293	1.793	0.073	-0.050	1.101	0.681	0.274	2.484	0.013	0.143	1.219
Disease 2	-0.041	0.475	-0.086	0.932	-0.974	0.892	0.594	0.643	0.924	0.356	-0.667	1.855
Disease 3	0.089	0.344	0.259	0.796	-0.586	0.764	0.171	0.262	0.652	0.515	-0.343	0.684
Disease 4	-0.314	0.428	-0.735	0.463	-1.153	0.525	0.906	0.759	1.193	0.233	-0.584	2.396
Disease 5	0.030	0.321	0.092	0.927	-0.600	0.660	1.149	0.815	1.409	0.159	-0.450	2.749
disease 6	-0.190	0.193	-0.987	0.324	-0.568	0.188	-0.143	0.161	-0.884	0.377	-0.459	0.174
ApoE4 hetero	-0.606	0.596	-1.017	0.310	-1.776	0.564	-0.704	0.641	-1.099	0.272	-1.962	0.553
ApoE4 homo	-2.245	3.207	-0.700	0.484	-8.538	4.049	0.641	2.105	0.305	0.761	-3.488	4.771
Y2024	-0.979	0.095	-10.32	0.000	-1.165	-0.793	-0.426	0.074	-5.775	0.000	-0.570	-0.281
	N		915	F		5.275	N		1424	F		4.316
	R2 (Between)		0.034	P(F)		0.000	R2 (Between)		0.053	P(F)		0.000
	R2 (Within)		0.237	σ2(u)		28.17	R2 (Within)		0.073	σ2(u)		44.54
	R2 (Overall)		0.040	σ2(ε)		1.750	R2 (Overall)		0.053	σ2(ε)		1.575
	Log-likelihood		-1558.1	σ2(u)/σ2(u+ε)		0.942	Log-likelihood		-2361.8	σ2(u)/σ2(u+ε)		0.966

Note: "Dietary" denotes the dietary habit cluster: "Dietary 1" is a dummy variable indicating whether an individual belongs to dietary habit cluster 1, and so on. "School": an individual has 13 or more years of education; "Non cohabitants": the individual lives alone; "Lifestyle change": the individual experienced a major change in lifestyle during the past year; "Paid": the individual is engaged in paid work; "Well off": the household is financially comfortable; "Alcohol": the individual has a drinking habit; "Smoking": the individual has a smoking habit; "Health 1 (or 2)": the subjective health status is "very" (or "fairly") healthy; "Exercise1": the individual engages in light exercise at least once per week; "Exercise2": the individual engages in moderate or vigorous exercise at least once per week; "Y 2024": that the survey year is 2024. "Disease1" through "Disease6" are indicators for diseases under treatment or with lasting effects: "Disease1" is hypertension, "Disease2" is diabetes, "Disease3" is hyperlipidemia, "Disease4" is heat disease, "Disease 5" is kindey disease,, and "Disease 6" is other diseases. "ApoE4 hetero" and "ApoE4 homo" are dummy variables indicating carriers of the ApoE4 heterozygous and homozygous genotypes, respectively.

Table S2 (continued). Regression results (Visceral fat level)

Visceral fat level	Males						Females					
	Coef.	S.E.	t	P(t)	[95%	C.I.]	Coef.	S.E.	t	P(t)	[95%	C.I.]
1[Age<65] × Dietary 1	11.92	0.410	29.05	0.000	11.11	12.72	6.333	0.219	28.88	0.000	5.903	6.763
1[Age<65] × Dietary 2	11.86	0.417	28.43	0.000	11.04	12.68	6.427	0.228	28.19	0.000	5.980	6.875
1[Age<65] × Dietary 3	11.96	0.421	28.39	0.000	11.13	12.78	6.321	0.216	29.23	0.000	5.897	6.745
1[Age<65] × Dietary 4	12.12	0.470	25.80	0.000	11.19	13.04	6.239	0.229	27.21	0.000	5.789	6.689
1[Age<65] × Dietary 5	11.89	0.402	29.61	0.000	11.10	12.68	6.396	0.211	30.33	0.000	5.983	6.810
1[Age<65] × Dietary 6	12.37	0.511	24.22	0.000	11.37	13.37	6.407	0.243	26.43	0.000	5.931	6.883
1[Age≥65] × Dietary 1	11.88	0.387	30.68	0.000	11.12	12.64	6.385	0.237	26.97	0.000	5.921	6.850
1[Age≥65] × Dietary 2	11.93	0.384	31.05	0.000	11.17	12.68	6.516	0.246	26.46	0.000	6.033	6.999
1[Age≥65] × Dietary 3	12.04	0.392	30.74	0.000	11.27	12.81	6.527	0.221	29.57	0.000	6.094	6.960
1[Age≥65] × Dietary 4	11.91	0.395	30.16	0.000	11.13	12.68	6.424	0.218	29.43	0.000	5.996	6.853
1[Age≥65] × Dietary 5	12.08	0.383	31.53	0.000	11.33	12.83	6.472	0.211	30.65	0.000	6.058	6.886
1[Age≥65] × Dietary 6	11.90	0.410	29.00	0.000	11.09	12.70	6.481	0.229	28.34	0.000	6.033	6.930
School	0.449	0.317	1.414	0.158	-0.174	1.072	-0.434	0.183	-2.375	0.018	-0.792	-0.075
Non cohabitants	0.187	0.341	0.548	0.584	-0.482	0.855	-0.239	0.155	-1.544	0.123	-0.542	0.065
Lifestyle change	0.043	0.085	0.511	0.610	-0.123	0.209	-0.019	0.050	-0.376	0.707	-0.117	0.080
Paid	0.002	0.152	0.012	0.990	-0.296	0.300	-0.329	0.092	-3.562	0.000	-0.510	-0.148
Well off	-0.073	0.129	-0.566	0.572	-0.327	0.181	0.014	0.071	0.192	0.848	-0.125	0.152
Alcohol	0.364	0.178	2.040	0.042	0.014	0.714	-0.094	0.087	-1.078	0.281	-0.265	0.077
Smoking	-0.164	0.295	-0.554	0.579	-0.744	0.416	0.066	0.249	0.265	0.791	-0.423	0.555
Healthy 1	-0.086	0.212	-0.407	0.684	-0.502	0.330	-0.349	0.136	-2.576	0.010	-0.615	-0.083
Healthy 2	0.011	0.153	0.072	0.942	-0.289	0.311	-0.292	0.107	-2.742	0.006	-0.501	-0.083
Exercise 1	-0.026	0.135	-0.190	0.850	-0.289	0.238	-0.026	0.079	-0.328	0.743	-0.181	0.129
Exercise 2	-0.228	0.116	-1.962	0.050	-0.455	0.000	0.053	0.068	0.782	0.434	-0.080	0.185
Disease 1	0.243	0.150	1.616	0.107	-0.052	0.538	0.278	0.110	2.539	0.011	0.063	0.493
Disease 2	-0.256	0.246	-1.039	0.299	-0.739	0.227	0.414	0.256	1.619	0.106	-0.088	0.916
Disease 3	0.063	0.175	0.361	0.719	-0.281	0.408	0.195	0.105	1.857	0.064	-0.011	0.400
Disease 4	-0.116	0.217	-0.536	0.592	-0.542	0.310	0.281	0.303	0.927	0.354	-0.313	0.875
Disease 5	-0.021	0.162	-0.129	0.897	-0.339	0.297	-0.029	0.330	-0.089	0.929	-0.677	0.618
disease 6	-0.109	0.097	-1.123	0.262	-0.300	0.082	0.009	0.065	0.143	0.886	-0.118	0.137
ApoE4 hetero	-0.163	0.340	-0.479	0.632	-0.830	0.504	-0.318	0.227	-1.399	0.162	-0.763	0.128
ApoE4 homo	-2.071	1.829	-1.132	0.258	-5.662	1.520	0.174	0.746	0.234	0.815	-1.288	1.637
Y2024	-0.301	0.048	-6.333	0.000	-0.394	-0.208	-0.096	0.030	-3.188	0.002	-0.154	-0.037
	N		915	F		2.604	N		1424	F		2.653
	R2 (Between)		0.028	P(F)		0.000	R2 (Between)		0.065	P(F)		0.000
	R2 (Within)		0.123	σ2(u)		9.271	R2 (Within)		0.032	σ2(u)		5.584
	R2 (Overall)		0.031	σ2(ε)		0.437	R2 (Overall)		0.064	σ2(ε)		0.264
	Log-likelihood		-922.6	σ2(u)/σ2(u+ε)		0.955	Log-likelihood		-1085.2	σ2(u)/σ2(u+ε)		0.955

Note: "Dietary" denotes the dietary habit cluster: "Dietary 1" is a dummy variable indicating whether an individual belongs to dietary habit cluster 1, and so on. "School": an individual has 13 or more years of education; "Non cohabitants": the individual lives alone; "Lifestyle change": the individual experienced a major change in lifestyle during the past year; "Paid": the individual is engaged in paid work; "Well off": the household is financially comfortable; "Alcohol": the individual has a drinking habit; "Smoking": the individual has a smoking habit; "Health 1 (or 2)": the subjective health status is "very" (or "fairly") healthy; "Exercise1": the individual engages in light exercise at least once per week; "Exercise2": the individual engages in moderate or vigorous exercise at least once per week; "Y 2024": that the survey year is 2024. "Disease1" through "Disease6" are indicators for diseases under treatment or with lasting effects: "Disease1" is hypertension, "Disease2" is diabetes, "Disease3" is hyperlipidemia, "Disease4" is heat disease, "Disease 5" is kindey disease,, and "Disease 6" is other diseases. "ApoE4 hetero" and "ApoE4 homo" are dummy variables indicating carriers of the ApoE4 heterozygous and homozygous genotypes, respectively.

Table S2 (continued). Regression results (Muscle mass to standard ratio)

Muscle mass ratio	Males						Females					
	Coef.	S.E.	t	P(t)	[95%	C.I.]	Coef.	S.E.	t	P(t)	[95%	C.I.]
1[Age<65] × Dietary 1	1.020	0.010	104.95	0.000	1.001	1.039	0.977	0.005	211.14	0.000	0.968	0.986
1[Age<65] × Dietary 2	1.015	0.010	102.76	0.000	0.995	1.034	0.982	0.005	204.54	0.000	0.972	0.991
1[Age<65] × Dietary 3	1.013	0.010	101.71	0.000	0.993	1.032	0.979	0.005	214.47	0.000	0.970	0.988
1[Age<65] × Dietary 4	1.023	0.011	92.46	0.000	1.001	1.044	0.977	0.005	202.76	0.000	0.968	0.987
1[Age<65] × Dietary 5	1.017	0.010	106.86	0.000	0.998	1.036	0.979	0.005	219.54	0.000	0.970	0.988
1[Age<65] × Dietary 6	1.037	0.012	86.46	0.000	1.013	1.060	0.978	0.005	192.45	0.000	0.968	0.988
1[Age≥65] × Dietary 1	1.013	0.009	110.21	0.000	0.995	1.031	0.984	0.005	197.89	0.000	0.974	0.993
1[Age≥65] × Dietary 2	1.012	0.009	111.00	0.000	0.994	1.030	0.983	0.005	190.21	0.000	0.973	0.994
1[Age≥65] × Dietary 3	1.013	0.009	109.05	0.000	0.995	1.031	0.984	0.005	211.59	0.000	0.975	0.994
1[Age≥65] × Dietary 4	1.022	0.009	109.12	0.000	1.003	1.040	0.982	0.005	213.31	0.000	0.973	0.991
1[Age≥65] × Dietary 5	1.013	0.009	111.40	0.000	0.995	1.031	0.982	0.005	219.89	0.000	0.973	0.991
1[Age≥65] × Dietary 6	1.004	0.010	103.44	0.000	0.985	1.023	0.985	0.005	204.74	0.000	0.976	0.995
School	0.006	0.008	0.831	0.407	-0.009	0.022	-0.012	0.004	-2.945	0.003	-0.020	-0.004
Non cohabitants	0.012	0.008	1.445	0.149	-0.004	0.027	-0.005	0.003	-1.585	0.113	-0.011	0.001
Lifestyle change	-0.005	0.002	-2.305	0.021	-0.008	-0.001	-0.002	0.001	-1.493	0.136	-0.004	0.001
Paid	0.002	0.004	0.420	0.675	-0.006	0.008	0.002	0.002	1.183	0.237	-0.002	0.006
Well off	-0.002	0.003	-0.642	0.521	-0.008	0.004	-0.001	0.001	-0.576	0.565	-0.004	0.002
Alcohol	0.006	0.004	1.516	0.130	-0.002	0.015	-0.001	0.002	-0.377	0.706	-0.004	0.003
Smoking	0.007	0.007	1.017	0.309	-0.007	0.021	0.001	0.005	0.246	0.805	-0.009	0.012
Healthy 1	0.004	0.005	0.828	0.408	-0.006	0.014	0.002	0.003	0.841	0.401	-0.003	0.008
Healthy 2	0.007	0.004	1.838	0.066	0.000	0.013	0.002	0.002	0.902	0.367	-0.002	0.006
Exercise 1	0.004	0.003	1.355	0.176	-0.002	0.010	-0.001	0.002	-0.620	0.535	-0.004	0.002
Exercise 2	-0.002	0.003	-0.597	0.551	-0.007	0.004	0.002	0.001	1.113	0.266	-0.001	0.004
Disease 1	0.003	0.004	0.855	0.393	-0.004	0.010	-0.001	0.002	-0.242	0.809	-0.005	0.004
Disease 2	-0.010	0.006	-1.761	0.079	-0.021	0.001	0.013	0.005	2.515	0.012	0.003	0.024
Disease 3	0.001	0.004	0.230	0.818	-0.007	0.009	0.005	0.002	2.503	0.012	0.001	0.010
Disease 4	0.007	0.005	1.303	0.193	-0.003	0.016	0.009	0.006	1.481	0.139	-0.003	0.021
Disease 5	-0.003	0.004	-0.754	0.451	-0.010	0.005	0.007	0.007	1.099	0.272	-0.006	0.021
disease 6	0.001	0.002	0.481	0.631	-0.003	0.006	0.001	0.001	0.383	0.702	-0.002	0.003
ApoE4 hetero	0.005	0.008	0.599	0.549	-0.011	0.021	-0.002	0.005	-0.388	0.698	-0.012	0.008
ApoE4 homo	-0.092	0.044	-2.065	0.039	-0.179	-0.005	0.009	0.016	0.555	0.579	-0.023	0.041
Y2024	0.010	0.001	9.019	0.000	0.008	0.012	0.009	0.001	14.58	0.000	0.008	0.010
	N		915	F		6.928	N		1424	F		34.499
	R2 (Between)		0.016	P(F)		0.000	R2 (Between)		0.051	P(F)		0.000
	R2 (Within)		0.225	σ2(u)		0.006	R2 (Within)		0.295	σ2(u)		0.003
	R2 (Overall)		0.021	σ2(ε)		0.000	R2 (Overall)		0.055	σ2(ε)		0.000
	Log-likelihood		2526.6	σ2(u)/σ2(u+ε)		0.959	Log-likelihood		4466.4	σ2(u)/σ2(u+ε)		0.962

Note: "Dietary" denotes the dietary habit cluster: "Dietary 1" is a dummy variable indicating whether an individual belongs to dietary habit cluster 1, and so on. "School": an individual has 13 or more years of education; "Non cohabitants": the individual lives alone; "Lifestyle change": the individual experienced a major change in lifestyle during the past year; "Paid": the individual is engaged in paid work; "Well off": the household is financially comfortable; "Alcohol": the individual has a drinking habit; "Smoking": the individual has a smoking habit; "Health 1 (or 2)": the subjective health status is "very" (or "fairly") healthy; "Exercise1": the individual engages in light exercise at least once per week; "Exercise2": the individual engages in moderate or vigorous exercise at least once per week; "Y 2024": that the survey year is 2024. "Disease1" through "Disease6" are indicators for diseases under treatment or with lasting effects: "Disease1" is hypertension, "Disease2" is diabetes, "Disease3" is hyperlipidemia, "Disease4" is heat disease, "Disease 5" is kindey disease,, and "Disease 6" is other diseases. "ApoE4 hetero" and "ApoE4 homo" are dummy variables indicating carriers of the ApoE4 heterozygous and homozygous genotypes, respectively.

Table S2 (continued). Regression results (Leg muscle mass score)

Leg muscle score	Males						Females					
	Coef.	S.E.	t	P(t)	[95%	C.I.]	Coef.	S.E.	t	P(t)	[95%	C.I.]
1[Age<65] × Dietary 1	89.37	1.055	84.75	0.000	87.30	91.44	93.05	0.705	131.90	0.000	91.66	94.43
1[Age<65] × Dietary 2	89.25	1.076	82.98	0.000	87.14	91.36	92.84	0.735	126.24	0.000	91.40	94.28
1[Age<65] × Dietary 3	89.04	1.091	81.59	0.000	86.90	91.19	92.81	0.696	133.36	0.000	91.45	94.18
1[Age<65] × Dietary 4	88.19	1.236	71.36	0.000	85.77	90.62	93.28	0.741	125.90	0.000	91.82	94.73
1[Age<65] × Dietary 5	88.87	1.033	86.07	0.000	86.85	90.90	93.02	0.677	137.39	0.000	91.69	94.35
1[Age<65] × Dietary 6	88.51	1.357	65.21	0.000	85.85	91.18	93.46	0.785	119.04	0.000	91.92	95.00
1[Age≥65] × Dietary 1	88.09	0.990	88.97	0.000	86.14	90.03	91.63	0.766	119.68	0.000	90.13	93.14
1[Age≥65] × Dietary 2	88.03	0.982	89.63	0.000	86.10	89.95	91.13	0.796	114.46	0.000	89.57	92.70
1[Age≥65] × Dietary 3	87.97	1.003	87.68	0.000	86.00	89.94	91.34	0.711	128.39	0.000	89.95	92.74
1[Age≥65] × Dietary 4	88.71	1.012	87.62	0.000	86.72	90.69	91.66	0.703	130.46	0.000	90.29	93.04
1[Age≥65] × Dietary 5	87.87	0.978	89.85	0.000	85.95	89.78	91.57	0.678	135.05	0.000	90.24	92.90
1[Age≥65] × Dietary 6	87.91	1.062	82.79	0.000	85.82	89.99	91.46	0.738	123.94	0.000	90.01	92.90
School	0.426	0.747	0.570	0.569	-1.040	1.892	2.321	0.562	4.130	0.000	1.219	3.424
Non cohabitants	-1.083	0.909	-1.191	0.234	-2.867	0.702	0.902	0.505	1.785	0.074	-0.089	1.892
Lifestyle change	-0.232	0.237	-0.978	0.328	-0.698	0.234	-0.024	0.167	-0.146	0.884	-0.352	0.303
Paid	0.791	0.414	1.912	0.056	-0.021	1.603	0.847	0.304	2.792	0.005	0.252	1.443
Well off	0.032	0.357	0.091	0.928	-0.668	0.732	0.174	0.234	0.745	0.457	-0.285	0.633
Alcohol	-0.453	0.476	-0.952	0.341	-1.387	0.481	0.763	0.286	2.667	0.008	0.202	1.325
Smoking	0.152	0.770	0.198	0.843	-1.360	1.664	0.617	0.809	0.763	0.445	-0.969	2.203
Healthy 1	0.856	0.591	1.449	0.148	-0.304	2.015	0.302	0.450	0.672	0.502	-0.581	1.185
Healthy 2	0.242	0.427	0.566	0.572	-0.596	1.080	0.644	0.354	1.823	0.069	-0.049	1.338
Exercise 1	0.160	0.375	0.427	0.670	-0.576	0.897	0.303	0.263	1.154	0.249	-0.212	0.818
Exercise 2	0.678	0.323	2.098	0.036	0.044	1.312	-0.233	0.224	-1.038	0.299	-0.673	0.207
Disease 1	-0.994	0.410	-2.424	0.016	-1.799	-0.189	-0.751	0.362	-2.076	0.038	-1.460	-0.042
Disease 2	-0.172	0.662	-0.260	0.795	-1.472	1.128	-0.943	0.841	-1.122	0.262	-2.593	0.706
Disease 3	-0.132	0.482	-0.275	0.784	-1.078	0.814	-0.485	0.346	-1.401	0.162	-1.165	0.194
Disease 4	0.633	0.601	1.054	0.292	-0.546	1.812	-0.919	0.997	-0.922	0.357	-2.876	1.037
Disease 5	-0.136	0.452	-0.302	0.763	-1.024	0.751	-1.385	1.100	-1.260	0.208	-3.542	0.772
disease 6	0.245	0.271	0.902	0.367	-0.288	0.777	0.253	0.216	1.169	0.243	-0.171	0.677
ApoE4 hetero	0.873	0.800	1.091	0.276	-0.697	2.443	0.503	0.698	0.720	0.472	-0.867	1.872
ApoE4 homo	-0.652	4.304	-0.152	0.880	-9.099	7.795	-0.926	2.294	-0.404	0.687	-5.426	3.574
Y2024	1.082	0.134	8.069	0.000	0.819	1.346	0.320	0.100	3.198	0.001	0.124	0.517
	N		915	F		5.755	N		1424	F		15.336
	R2 (Between)		0.075	P(F)		0.000	R2 (Between)		0.115	P(F)		0.000
	R2 (Within)		0.126	σ2(u)		49.30	R2 (Within)		0.008	σ2(u)		51.75
	R2 (Overall)		0.076	σ2(ε)		3.425	R2 (Overall)		0.114	σ2(ε)		2.913
	Log-likelihood		-1876.7	σ2(u)/σ2(u+ε)		0.935	Log-likelihood		-2806.0	σ2(u)/σ2(u+ε)		0.947

Note: "Dietary" denotes the dietary habit cluster: "Dietary 1" is a dummy variable indicating whether an individual belongs to dietary habit cluster 1, and so on. "School": an individual has 13 or more years of education; "Non cohabitants": the individual lives alone; "Lifestyle change": the individual experienced a major change in lifestyle during the past year; "Paid": the individual is engaged in paid work; "Well off": the household is financially comfortable; "Alcohol": the individual has a drinking habit; "Smoking": the individual has a smoking habit; "Health 1 (or 2)": the subjective health status is "very" (or "fairly") healthy; "Exercise1": the individual engages in light exercise at least once per week; "Exercise2": the individual engages in moderate or vigorous exercise at least once per week; "Y 2024": that the survey year is 2024. "Disease1" through "Disease6" are indicators for diseases under treatment or with lasting effects: "Disease1" is hypertension, "Disease2" is diabetes, "Disease3" is hyperlipidemia, "Disease4" is heat disease, "Disease 5" is kindey disease,, and "Disease 6" is other diseases. "ApoE4 hetero" and "ApoE4 homo" are dummy variables indicating carriers of the ApoE4 heterozygous and homozygous genotypes, respectively.

Table S2 (continued). Regression results (Grip strength)

Grip strength	Males						Females					
	Coef.	S.E.	t	P(t)	[95%	C.I.]	Coef.	S.E.	t	P(t)	[95%	C.I.]
1[Age<65] × Dietary 1	37.51	0.953	39.37	0.000	35.64	39.38	23.32	0.478	48.83	0.000	22.38	24.26
1[Age<65] × Dietary 2	37.08	0.977	37.94	0.000	35.17	39.00	23.99	0.508	47.26	0.000	22.99	24.98
1[Age<65] × Dietary 3	38.55	1.002	38.49	0.000	36.59	40.52	23.77	0.479	49.60	0.000	22.83	24.71
1[Age<65] × Dietary 4	37.91	1.160	32.70	0.000	35.64	40.19	23.17	0.526	44.09	0.000	22.14	24.20
1[Age<65] × Dietary 5	38.38	0.936	40.99	0.000	36.54	40.22	23.51	0.456	51.53	0.000	22.62	24.41
1[Age<65] × Dietary 6	38.84	1.292	30.08	0.000	36.31	41.38	23.42	0.558	41.99	0.000	22.33	24.52
1[Age≥65] × Dietary 1	36.61	0.891	41.10	0.000	34.86	38.36	23.25	0.542	42.89	0.000	22.19	24.31
1[Age≥65] × Dietary 2	36.19	0.885	40.89	0.000	34.46	37.93	22.86	0.550	41.56	0.000	21.78	23.94
1[Age≥65] × Dietary 3	36.18	0.906	39.95	0.000	34.40	37.95	23.18	0.495	46.82	0.000	22.21	24.15
1[Age≥65] × Dietary 4	37.01	0.915	40.43	0.000	35.21	38.80	22.70	0.483	47.03	0.000	21.75	23.65
1[Age≥65] × Dietary 5	37.15	0.878	42.30	0.000	35.43	38.88	22.73	0.459	49.49	0.000	21.83	23.63
1[Age≥65] × Dietary 6	36.32	0.977	37.19	0.000	34.40	38.24	22.93	0.514	44.62	0.000	21.92	23.93
School	0.242	0.584	0.415	0.679	-0.904	1.389	0.103	0.266	0.389	0.698	-0.418	0.624
Non cohabitants	0.278	0.841	0.331	0.741	-1.372	1.928	0.100	0.337	0.297	0.767	-0.561	0.761
Lifestyle change	-0.202	0.242	-0.834	0.405	-0.678	0.274	-0.056	0.142	-0.395	0.693	-0.335	0.223
Paid	0.538	0.397	1.354	0.176	-0.242	1.318	0.346	0.218	1.585	0.113	-0.082	0.774
Well off	0.436	0.352	1.238	0.216	-0.255	1.127	-0.147	0.186	-0.789	0.430	-0.511	0.218
Alcohol	0.550	0.439	1.253	0.211	-0.312	1.411	-0.112	0.203	-0.549	0.583	-0.511	0.287
Smoking	0.803	0.685	1.173	0.241	-0.540	2.147	0.612	0.514	1.191	0.234	-0.396	1.620
Healthy 1	-0.432	0.597	-0.724	0.469	-1.603	0.739	0.620	0.364	1.702	0.089	-0.095	1.334
Healthy 2	-0.155	0.433	-0.358	0.720	-1.005	0.694	0.666	0.285	2.333	0.020	0.106	1.225
Exercise 1	0.282	0.380	0.741	0.459	-0.464	1.028	0.227	0.216	1.050	0.294	-0.197	0.652
Exercise 2	0.004	0.325	0.011	0.991	-0.634	0.642	0.231	0.181	1.278	0.201	-0.124	0.586
Disease 1	0.180	0.395	0.455	0.649	-0.596	0.955	0.107	0.268	0.399	0.690	-0.419	0.633
Disease 2	-1.535	0.621	-2.471	0.014	-2.755	-0.316	-0.183	0.604	-0.303	0.762	-1.367	1.001
Disease 3	0.389	0.471	0.827	0.409	-0.535	1.314	0.030	0.263	0.112	0.911	-0.486	0.545
Disease 4	-0.083	0.596	-0.140	0.889	-1.254	1.087	1.543	0.728	2.120	0.034	0.115	2.971
Disease 5	0.080	0.457	0.174	0.862	-0.817	0.976	-1.049	0.922	-1.138	0.256	-2.857	0.760
disease 6	-0.423	0.274	-1.541	0.124	-0.961	0.116	-0.105	0.175	-0.602	0.547	-0.449	0.238
ApoE4 hetero	0.397	0.626	0.635	0.526	-0.831	1.625	-0.052	0.328	-0.160	0.873	-0.696	0.592
ApoE4 homo	-0.648	3.360	-0.193	0.847	-7.242	5.946	-1.206	1.080	-1.116	0.265	-3.325	0.913
Y2024	-0.079	0.140	-0.566	0.572	-0.354	0.196	-0.045	0.090	-0.499	0.618	-0.221	0.132
	N		915	F		2.447	N		1424	F		3.503
	R2 (Between)		0.067	P(F)		0.000	R2 (Between)		0.036	P(F)		0.000
	R2 (Within)		0.050	σ2(u)		31.16	R2 (Within)		0.023	σ2(u)		10.96
	R2 (Overall)		0.065	σ2(ε)		4.068	R2 (Overall)		0.036	σ2(ε)		2.570
	Log-likelihood		-1923.5	σ2(u)/σ2(u+ε)		0.885	Log-likelihood		-2680.4	σ2(u)/σ2(u+ε)		0.810

Note: “Dietary” denotes the dietary habit cluster: “Dietary 1” is a dummy variable indicating whether an individual belongs to dietary habit cluster 1, and so on. “School”: an individual has 13 or more years of education; “Non cohabitants”: the individual lives alone; “Lifestyle change”: the individual experienced a major change in lifestyle during the past year; “Paid”: the individual is engaged in paid work; “Well off”: the household is financially comfortable; “Alcohol”: the individual has a drinking habit; “Smoking”: the individual has a smoking habit; “Health 1 (or 2)”: the subjective health status is “very” (or “fairly”) healthy; “Exercise1”: the individual engages in light exercise at least once per week; “Exercise2”: the individual engages in moderate or vigorous exercise at least once per week; “Y 2024”: that the survey year is 2024. “Disease1” through “Disease6” are indicators for diseases under treatment or with lasting effects: “Disease1” is hypertension, “Disease2” is diabetes, “Disease3” is hyperlipidemia, “Disease4” is heat disease, “Disease 5” is kindey disease,, and “Disease 6” is other diseases. “ApoE4 hetero” and “ApoE4 homo” are dummy variables indicating carriers of the ApoE4 heterozygous and homozygous genotypes, respectively.

Table S2 (continued). Regression results (Sit-to-stand test)

Sit-to-stand test	Males						Females					
	Coef.	S.E.	t	P(t)	[95%	C.I.]	Coef.	S.E.	t	P(t)	[95%	C.I.]
1[Age<65] × Dietary 1	6.728	0.362	18.59	0.000	6.018	7.439	6.387	0.270	23.63	0.000	5.856	6.917
1[Age<65] × Dietary 2	6.881	0.383	17.97	0.000	6.130	7.633	6.342	0.291	21.77	0.000	5.770	6.913
1[Age<65] × Dietary 3	6.793	0.404	16.80	0.000	6.000	7.587	6.351	0.280	22.70	0.000	5.803	6.900
1[Age<65] × Dietary 4	7.741	0.487	15.91	0.000	6.785	8.696	6.187	0.314	19.72	0.000	5.572	6.803
1[Age<65] × Dietary 5	6.624	0.368	18.02	0.000	5.903	7.346	6.397	0.260	24.56	0.000	5.886	6.908
1[Age<65] × Dietary 6	6.974	0.554	12.59	0.000	5.887	8.062	6.374	0.325	19.59	0.000	5.735	7.012
1[Age≥65] × Dietary 1	7.020	0.347	20.26	0.000	6.340	7.700	6.880	0.319	21.59	0.000	6.255	7.505
1[Age≥65] × Dietary 2	7.149	0.346	20.65	0.000	6.469	7.828	7.096	0.309	23.00	0.000	6.491	7.702
1[Age≥65] × Dietary 3	7.267	0.353	20.56	0.000	6.573	7.960	6.872	0.291	23.59	0.000	6.301	7.444
1[Age≥65] × Dietary 4	7.111	0.362	19.67	0.000	6.402	7.821	6.522	0.278	23.46	0.000	5.977	7.067
1[Age≥65] × Dietary 5	7.191	0.344	20.93	0.000	6.517	7.866	6.550	0.265	24.74	0.000	6.031	7.069
1[Age≥65] × Dietary 6	7.104	0.412	17.26	0.000	6.296	7.911	6.998	0.297	23.57	0.000	6.416	7.581
School	-0.322	0.153	-2.107	0.035	-0.622	-0.022	0.138	0.115	1.201	0.230	-0.087	0.362
Non cohabitants	0.656	0.292	2.247	0.025	0.083	1.229	0.104	0.169	0.616	0.538	-0.227	0.436
Lifestyle change	-0.046	0.126	-0.364	0.716	-0.293	0.201	-0.076	0.096	-0.790	0.430	-0.264	0.112
Paid	-0.210	0.153	-1.376	0.169	-0.509	0.090	-0.169	0.117	-1.445	0.149	-0.398	0.060
Well off	-0.092	0.151	-0.611	0.541	-0.387	0.203	-0.074	0.111	-0.663	0.508	-0.291	0.144
Alcohol	-0.185	0.150	-1.233	0.218	-0.479	0.109	-0.004	0.107	-0.041	0.967	-0.215	0.206
Smoking	-0.141	0.215	-0.657	0.512	-0.564	0.281	0.081	0.250	0.326	0.744	-0.408	0.571
Healthy 1	-0.420	0.289	-1.452	0.147	-0.988	0.148	-0.365	0.225	-1.620	0.106	-0.807	0.077
Healthy 2	-0.093	0.214	-0.435	0.664	-0.513	0.327	-0.416	0.175	-2.374	0.018	-0.759	-0.072
Exercise 1	-0.346	0.188	-1.839	0.066	-0.716	0.023	-0.318	0.138	-2.313	0.021	-0.589	-0.048
Exercise 2	-0.223	0.155	-1.440	0.150	-0.527	0.081	-0.213	0.112	-1.899	0.058	-0.434	0.007
Disease 1	-0.040	0.153	-0.263	0.793	-0.341	0.260	0.247	0.147	1.678	0.094	-0.042	0.535
Disease 2	0.339	0.221	1.534	0.125	-0.095	0.772	-0.040	0.323	-0.123	0.902	-0.674	0.595
Disease 3	-0.100	0.192	-0.520	0.603	-0.476	0.276	0.019	0.148	0.131	0.896	-0.272	0.310
Disease 4	0.122	0.263	0.464	0.643	-0.394	0.638	0.704	0.393	1.790	0.074	-0.067	1.474
Disease 5	-0.464	0.217	-2.139	0.033	-0.890	-0.038	-0.137	0.601	-0.227	0.820	-1.316	1.043
disease 6	-0.019	0.132	-0.146	0.884	-0.279	0.241	-0.090	0.108	-0.831	0.406	-0.302	0.122
ApoE4 hetero	-0.066	0.164	-0.404	0.686	-0.388	0.256	0.047	0.141	0.336	0.737	-0.229	0.324
ApoE4 homo	1.643	0.873	1.883	0.060	-0.069	3.355	0.205	0.466	0.441	0.659	-0.708	1.119
Y2024	0.221	0.091	2.442	0.015	0.043	0.399	0.189	0.071	2.647	0.008	0.049	0.329
	N		915	F		2.084	N		1424	F		2.371
	R2 (Between)		0.101	P(F)		0.001	R2 (Between)		0.082	P(F)		0.000
	R2 (Within)		0.028	σ2(u)		1.304	R2 (Within)		0.005	σ2(u)		1.361
	R2 (Overall)		0.081	σ2(ε)		1.806	R2 (Overall)		0.064	σ2(ε)		1.690
	Log-likelihood		-1550.1	σ2(u)/σ2(u+ε)		0.419	Log-likelihood		-2384.9	σ2(u)/σ2(u+ε)		0.446

Note: "Dietary" denotes the dietary habit cluster: "Dietary 1" is a dummy variable indicating whether an individual belongs to dietary habit cluster 1, and so on. "School": an individual has 13 or more years of education; "Non cohabitants": the individual lives alone; "Lifestyle change": the individual experienced a major change in lifestyle during the past year; "Paid": the individual is engaged in paid work; "Well off": the household is financially comfortable; "Alcohol": the individual has a drinking habit; "Smoking": the individual has a smoking habit; "Health 1 (or 2)": the subjective health status is "very" (or "fairly") healthy; "Exercise1": the individual engages in light exercise at least once per week; "Exercise2": the individual engages in moderate or vigorous exercise at least once per week; "Y 2024": that the survey year is 2024. "Disease1" through "Disease6" are indicators for diseases under treatment or with lasting effects: "Disease1" is hypertension, "Disease2" is diabetes, "Disease3" is hyperlipidemia, "Disease4" is heat disease, "Disease 5" is kindey disease,, and "Disease 6" is other diseases. "ApoE4 hetero" and "ApoE4 homo" are dummy variables indicating carriers of the ApoE4 heterozygous and homozygous genotypes, respectively.

Table S2 (continued). Regression results (Systolic blood pressure)

SBP	Males						Females					
	Coef.	S.E.	t	P(t)	[95%	C.I.]	Coef.	S.E.	t	P(t)	[95%	C.I.]
1[Age<65] × Dietary 1	135.88	3.290	41.30	0.000	129.43	142.34	131.48	2.402	54.73	0.000	126.77	136.20
1[Age<65] × Dietary 2	133.02	3.408	39.04	0.000	126.33	139.71	131.12	2.556	51.31	0.000	126.11	136.13
1[Age<65] × Dietary 3	138.81	3.544	39.17	0.000	131.85	145.77	132.95	2.415	55.05	0.000	128.21	137.68
1[Age<65] × Dietary 4	138.79	4.190	33.13	0.000	130.57	147.02	131.13	2.653	49.43	0.000	125.93	136.34
1[Age<65] × Dietary 5	139.20	3.266	42.62	0.000	132.79	145.62	132.87	2.296	57.88	0.000	128.37	137.37
1[Age<65] × Dietary 6	137.54	4.730	29.08	0.000	128.25	146.82	132.12	2.813	46.96	0.000	126.60	137.64
1[Age≥65] × Dietary 1	140.75	3.088	45.58	0.000	134.69	146.81	138.89	2.735	50.78	0.000	133.53	144.26
1[Age≥65] × Dietary 2	140.55	3.080	45.64	0.000	134.50	146.59	138.09	2.768	49.90	0.000	132.66	143.52
1[Age≥65] × Dietary 3	141.06	3.149	44.79	0.000	134.88	147.25	139.20	2.497	55.74	0.000	134.30	144.10
1[Age≥65] × Dietary 4	140.97	3.192	44.16	0.000	134.71	147.24	138.57	2.431	57.00	0.000	133.80	143.34
1[Age≥65] × Dietary 5	142.57	3.046	46.81	0.000	136.59	148.55	138.22	2.313	59.76	0.000	133.68	142.75
1[Age≥65] × Dietary 6	138.84	3.495	39.72	0.000	131.98	145.70	138.31	2.590	53.41	0.000	133.23	143.39
School	-0.497	1.700	-0.292	0.770	-3.832	2.839	-2.914	1.306	-2.230	0.026	-5.476	-0.351
Non cohabitants	3.134	2.848	1.101	0.271	-2.456	8.724	-0.680	1.683	-0.404	0.686	-3.981	2.622
Lifestyle change	1.000	0.961	1.040	0.298	-0.886	2.885	-0.268	0.724	-0.370	0.712	-1.689	1.153
Paid	0.004	1.414	0.003	0.998	-2.770	2.778	0.261	1.096	0.238	0.812	-1.889	2.411
Well off	0.528	1.308	0.404	0.687	-2.040	3.095	0.271	0.940	0.288	0.773	-1.573	2.116
Alcohol	1.147	1.480	0.775	0.438	-1.757	4.051	0.589	1.020	0.577	0.564	-1.413	2.590
Smoking	0.197	2.213	0.089	0.929	-4.146	4.540	-1.976	2.558	-0.772	0.440	-6.994	3.043
Healthy 1	-2.230	2.314	-0.964	0.335	-6.771	2.311	1.553	1.846	0.841	0.401	-2.069	5.175
Healthy 2	-0.647	1.690	-0.383	0.702	-3.965	2.670	-0.292	1.447	-0.202	0.840	-3.131	2.546
Exercise 1	-1.039	1.483	-0.701	0.484	-3.950	1.872	0.649	1.099	0.591	0.555	-1.507	2.806
Exercise 2	-1.683	1.252	-1.344	0.179	-4.141	0.775	-0.926	0.917	-1.010	0.313	-2.726	0.873
Disease 1	-0.669	1.411	-0.475	0.635	-3.438	2.099	3.844	1.350	2.849	0.005	1.197	6.492
Disease 2	-1.285	2.137	-0.601	0.548	-5.479	2.910	-1.148	3.031	-0.379	0.705	-7.094	4.798
Disease 3	0.318	1.719	0.185	0.853	-3.055	3.691	-2.071	1.325	-1.563	0.118	-4.671	0.529
Disease 4	-3.485	2.240	-1.556	0.120	-7.882	0.911	2.344	3.659	0.641	0.522	-4.833	9.521
Disease 5	0.328	1.765	0.186	0.853	-3.136	3.791	6.050	4.692	1.289	0.198	-3.155	15.26
disease 6	-0.399	1.064	-0.375	0.708	-2.486	1.689	0.533	0.888	0.601	0.548	-1.209	2.275
ApoE4 hetero	1.424	1.821	0.782	0.435	-2.151	4.998	1.035	1.613	0.642	0.521	-2.129	4.200
ApoE4 homo	9.403	9.750	0.964	0.335	-9.734	28.54	-2.447	5.310	-0.461	0.645	-12.86	7.969
Y2024	-6.212	0.585	-10.62	0.000	-7.359	-5.064	-4.806	0.462	-10.41	0.000	-5.712	-3.900
	N		915	F		6.368	N		1424	F		8.519
	R2 (Between)		0.037	P(F)		0.000	R2 (Between)		0.095	P(F)		0.000
	R2 (Within)		0.223	σ2(u)		240.18	R2 (Within)		0.121	σ2(u)		254.85
	R2 (Overall)		0.060	σ2(ε)		72.27	R2 (Overall)		0.098	σ2(ε)		66.23
	Log-likelihood		-3242.2	σ2(u)/σ2(u+ε)		0.769	Log-likelihood		-5012.1	σ2(u)/σ2(u+ε)		0.794

Note: "Dietary" denotes the dietary habit cluster: "Dietary 1" is a dummy variable indicating whether an individual belongs to dietary habit cluster 1, and so on. "School": an individual has 13 or more years of education; "Non cohabitants": the individual lives alone; "Lifestyle change": the individual experienced a major change in lifestyle during the past year; "Paid": the individual is engaged in paid work; "Well off": the household is financially comfortable; "Alcohol": the individual has a drinking habit; "Smoking": the individual has a smoking habit; "Health 1 (or 2)": the subjective health status is "very" (or "fairly") healthy; "Exercise1": the individual engages in light exercise at least once per week; "Exercise2": the individual engages in moderate or vigorous exercise at least once per week; "Y 2024": that the survey year is 2024. "Disease1" through "Disease6" are indicators for diseases under treatment or with lasting effects: "Disease1" is hypertension, "Disease2" is diabetes, "Disease3" is hyperlipidemia, "Disease4" is heat disease, "Disease 5" is kindey disease,, and "Disease 6" is other diseases. "ApoE4 hetero" and "ApoE4 homo" are dummy variables indicating carriers of the ApoE4 heterozygous and homozygous genotypes, respectively.

Table S2 (continued). Regression results (Heart rate)

Heart rate	Males						Females					
	Coef.	S.E.	t	P(t)	[95%	C.I.]	Coef.	S.E.	t	P(t)	[95%	C.I.]
1[Age<65] × Dietary 1	76.32	2.251	33.90	0.000	71.90	80.74	80.09	1.539	52.06	0.000	77.08	83.11
1[Age<65] × Dietary 2	73.21	2.342	31.27	0.000	68.62	77.81	80.66	1.641	49.15	0.000	77.44	83.88
1[Age<65] × Dietary 3	76.52	2.446	31.28	0.000	71.72	81.32	79.26	1.555	50.98	0.000	76.21	82.31
1[Age<65] × Dietary 4	76.06	2.908	26.16	0.000	70.35	81.77	79.20	1.716	46.16	0.000	75.83	82.56
1[Age<65] × Dietary 5	74.22	2.246	33.05	0.000	69.82	78.63	79.66	1.472	54.12	0.000	76.77	82.54
1[Age<65] × Dietary 6	72.03	3.293	21.87	0.000	65.57	78.49	80.61	1.814	44.45	0.000	77.05	84.17
1[Age≥65] × Dietary 1	75.74	2.120	35.72	0.000	71.58	79.90	79.67	1.765	45.14	0.000	76.20	83.13
1[Age≥65] × Dietary 2	77.35	2.117	36.54	0.000	73.20	81.51	81.33	1.772	45.90	0.000	77.86	84.81
1[Age≥65] × Dietary 3	75.25	2.164	34.78	0.000	71.00	79.49	80.95	1.610	50.28	0.000	77.79	84.11
1[Age≥65] × Dietary 4	76.77	2.197	34.95	0.000	72.46	81.08	79.02	1.563	50.57	0.000	75.96	82.09
1[Age≥65] × Dietary 5	74.15	2.093	35.43	0.000	70.04	78.26	80.76	1.485	54.37	0.000	77.85	83.67
1[Age≥65] × Dietary 6	74.08	2.427	30.52	0.000	69.32	78.84	79.33	1.666	47.62	0.000	76.07	82.60
School	0.205	1.104	0.186	0.853	-1.961	2.371	-0.349	0.792	-0.441	0.659	-1.902	1.204
Non cohabitants	2.777	1.922	1.445	0.149	-0.995	6.549	-1.724	1.057	-1.631	0.103	-3.797	0.350
Lifestyle change	0.595	0.687	0.866	0.387	-0.753	1.943	0.302	0.480	0.630	0.529	-0.639	1.243
Paid	-1.925	0.968	-1.988	0.047	-3.825	-0.025	-2.391	0.698	-3.428	0.001	-3.759	-1.022
Well off	0.046	0.910	0.051	0.960	-1.740	1.832	-0.249	0.611	-0.408	0.683	-1.448	0.949
Alcohol	-2.857	0.996	-2.868	0.004	-4.811	-0.902	-0.472	0.648	-0.729	0.466	-1.743	0.798
Smoking	4.567	1.473	3.101	0.002	1.676	7.457	2.929	1.595	1.836	0.067	-0.200	6.058
Healthy 1	-1.996	1.638	-1.218	0.223	-5.211	1.219	-2.915	1.206	-2.417	0.016	-5.281	-0.549
Healthy 2	1.116	1.200	0.930	0.353	-1.239	3.471	-1.568	0.944	-1.661	0.097	-3.421	0.284
Exercise 1	-1.704	1.053	-1.618	0.106	-3.771	0.363	-0.931	0.721	-1.290	0.197	-2.346	0.484
Exercise 2	-1.118	0.884	-1.264	0.207	-2.853	0.618	0.078	0.599	0.130	0.896	-1.097	1.254
Disease 1	0.409	0.967	0.423	0.672	-1.490	2.308	0.179	0.863	0.208	0.835	-1.514	1.873
Disease 2	2.028	1.447	1.401	0.162	-0.813	4.868	1.763	1.929	0.914	0.361	-2.020	5.547
Disease 3	1.061	1.187	0.894	0.372	-1.269	3.391	-1.274	0.852	-1.495	0.135	-2.946	0.398
Disease 4	-1.040	1.565	-0.665	0.506	-4.111	2.031	-0.001	2.333	0.000	1.000	-4.578	4.576
Disease 5	-0.210	1.246	-0.169	0.866	-2.656	2.235	1.713	3.092	0.554	0.580	-4.353	7.780
disease 6	0.540	0.753	0.717	0.474	-0.938	2.017	0.150	0.580	0.259	0.796	-0.988	1.287
ApoE4 hetero	-0.128	1.183	-0.109	0.914	-2.450	2.194	-0.357	0.977	-0.365	0.715	-2.273	1.560
ApoE4 homo	8.952	6.326	1.415	0.157	-3.463	21.37	5.063	3.217	1.574	0.116	-1.248	11.37
Y2024	-1.465	0.429	-3.413	0.001	-2.308	-0.623	-1.764	0.312	-5.659	0.000	-2.376	-1.153
	N		915	F		3.387	N		1424	F		5.906
	R2 (Between)		0.102	P(F)		0.000	R2 (Between)		0.031	P(F)		0.000
	R2 (Within)		0.064	σ2(u)		96.60	R2 (Within)		0.082	σ2(u)		92.70
	R2 (Overall)		0.097	σ2(ε)		39.36	R2 (Overall)		0.037	σ2(ε)		31.35
	Log-likelihood		-2963.0	σ2(u)/σ2(u+ε)		0.711	Log-likelihood		-4459.0	σ2(u)/σ2(u+ε)		0.747

Note: "Dietary" denotes the dietary habit cluster: "Dietary 1" is a dummy variable indicating whether an individual belongs to dietary habit cluster 1, and so on. "School": an individual has 13 or more years of education; "Non cohabitants": the individual lives alone; "Lifestyle change": the individual experienced a major change in lifestyle during the past year; "Paid": the individual is engaged in paid work; "Well off": the household is financially comfortable; "Alcohol": the individual has a drinking habit; "Smoking": the individual has a smoking habit; "Health 1 (or 2)": the subjective health status is "very" (or "fairly") healthy; "Exercise1": the individual engages in light exercise at least once per week; "Exercise2": the individual engages in moderate or vigorous exercise at least once per week; "Y 2024": that the survey year is 2024. "Disease1" through "Disease6" are indicators for diseases under treatment or with lasting effects: "Disease1" is hypertension, "Disease2" is diabetes, "Disease3" is hyperlipidemia, "Disease4" is heat disease, "Disease 5" is kindey disease,, and "Disease 6" is other diseases. "ApoE4 hetero" and "ApoE4 homo" are dummy variables indicating carriers of the ApoE4 heterozygous and homozygous genotypes, respectively.

Table S2 (continued). Regression results (Total protein)

Total protein	Males						Females					
	Coef.	S.E.	t	P(t)	[95%	C.I.]	Coef.	S.E.	t	P(t)	[95%	C.I.]
1[Age<65] × Dietary 1	7.522	0.077	97.68	0.000	7.371	7.673	7.486	0.053	142.05	0.000	7.383	7.590
1[Age<65] × Dietary 2	7.465	0.080	93.50	0.000	7.308	7.622	7.497	0.056	133.73	0.000	7.387	7.607
1[Age<65] × Dietary 3	7.545	0.083	90.78	0.000	7.382	7.708	7.523	0.053	141.99	0.000	7.419	7.627
1[Age<65] × Dietary 4	7.467	0.098	75.90	0.000	7.274	7.660	7.493	0.058	128.72	0.000	7.378	7.607
1[Age<65] × Dietary 5	7.507	0.077	98.09	0.000	7.357	7.658	7.515	0.050	149.21	0.000	7.416	7.614
1[Age<65] × Dietary 6	7.382	0.111	66.43	0.000	7.164	7.600	7.558	0.062	122.46	0.000	7.437	7.679
1[Age≥65] × Dietary 1	7.522	0.072	104.00	0.000	7.380	7.664	7.448	0.060	124.13	0.000	7.331	7.566
1[Age≥65] × Dietary 2	7.592	0.072	105.22	0.000	7.450	7.734	7.495	0.061	123.44	0.000	7.375	7.614
1[Age≥65] × Dietary 3	7.585	0.074	102.81	0.000	7.440	7.730	7.494	0.055	136.80	0.000	7.387	7.602
1[Age≥65] × Dietary 4	7.612	0.075	101.76	0.000	7.465	7.759	7.493	0.053	140.50	0.000	7.389	7.598
1[Age≥65] × Dietary 5	7.577	0.071	106.19	0.000	7.437	7.717	7.502	0.051	147.86	0.000	7.402	7.601
1[Age≥65] × Dietary 6	7.548	0.082	91.99	0.000	7.387	7.709	7.498	0.057	131.99	0.000	7.386	7.609
School	-0.059	0.039	-1.500	0.134	-0.136	0.018	-0.022	0.029	-0.776	0.438	-0.078	0.034
Non cohabitants	-0.072	0.067	-1.087	0.277	-0.203	0.058	-0.059	0.037	-1.609	0.108	-0.132	0.013
Lifestyle change	-0.006	0.023	-0.253	0.800	-0.050	0.039	-0.042	0.016	-2.655	0.008	-0.073	-0.011
Paid	-0.021	0.033	-0.639	0.523	-0.086	0.044	-0.034	0.024	-1.418	0.156	-0.081	0.013
Well off	0.031	0.031	1.012	0.312	-0.029	0.091	-0.021	0.021	-1.001	0.317	-0.061	0.020
Alcohol	-0.097	0.035	-2.797	0.005	-0.164	-0.029	0.033	0.022	1.462	0.144	-0.011	0.077
Smoking	-0.009	0.052	-0.180	0.857	-0.110	0.092	-0.096	0.056	-1.705	0.089	-0.206	0.014
Healthy 1	-0.032	0.055	-0.593	0.553	-0.139	0.075	-0.087	0.041	-2.141	0.032	-0.166	-0.007
Healthy 2	-0.060	0.040	-1.508	0.132	-0.138	0.018	-0.046	0.032	-1.436	0.151	-0.108	0.017
Exercise 1	0.014	0.035	0.409	0.683	-0.054	0.083	-0.002	0.024	-0.079	0.937	-0.049	0.045
Exercise 2	-0.018	0.030	-0.622	0.534	-0.076	0.040	-0.021	0.020	-1.048	0.295	-0.061	0.018
Disease 1	-0.003	0.033	-0.095	0.924	-0.068	0.062	0.070	0.030	2.347	0.019	0.011	0.128
Disease 2	0.104	0.050	2.085	0.037	0.006	0.202	0.020	0.067	0.299	0.765	-0.111	0.150
Disease 3	0.083	0.040	2.052	0.041	0.004	0.162	-0.020	0.029	-0.698	0.485	-0.077	0.037
Disease 4	-0.055	0.053	-1.038	0.300	-0.158	0.049	0.057	0.080	0.704	0.481	-0.101	0.214
Disease 5	-0.055	0.042	-1.319	0.188	-0.137	0.027	0.023	0.103	0.220	0.826	-0.179	0.225
disease 6	0.004	0.025	0.177	0.860	-0.045	0.054	0.000	0.020	0.010	0.992	-0.038	0.038
ApoE4 hetero	-0.020	0.042	-0.472	0.637	-0.103	0.063	-0.052	0.035	-1.463	0.144	-0.121	0.018
ApoE4 homo	0.043	0.226	0.188	0.851	-0.401	0.485	0.178	0.116	1.530	0.126	-0.050	0.407
Y2024	0.026	0.014	1.842	0.066	-0.002	0.053	0.040	0.010	3.950	0.000	0.020	0.060
	N		915	F		7.773	N		1424	F		19.487
	R2 (Between)		0.078	P(F)		0.000	R2 (Between)		0.047	P(F)		0.000
	R2 (Within)		0.025	σ2(u)		0.127	R2 (Within)		0.037	σ2(u)		0.126
	R2 (Overall)		0.073	σ2(ε)		0.041	R2 (Overall)		0.046	σ2(ε)		0.033
	Log-likelihood		179.6	σ2(u)/σ2(u+ε)		0.758	Log-likelihood		426.5	σ2(u)/σ2(u+ε)		0.793

Note: "Dietary" denotes the dietary habit cluster: "Dietary 1" is a dummy variable indicating whether an individual belongs to dietary habit cluster 1, and so on. "School": an individual has 13 or more years of education; "Non cohabitants": the individual lives alone; "Lifestyle change": the individual experienced a major change in lifestyle during the past year; "Paid": the individual is engaged in paid work; "Well off": the household is financially comfortable; "Alcohol": the individual has a drinking habit; "Smoking": the individual has a smoking habit; "Health 1 (or 2)": the subjective health status is "very" (or "fairly") healthy; "Exercise1": the individual engages in light exercise at least once per week; "Exercise2": the individual engages in moderate or vigorous exercise at least once per week; "Y 2024": that the survey year is 2024. "Disease1" through "Disease6" are indicators for diseases under treatment or with lasting effects: "Disease1" is hypertension, "Disease2" is diabetes, "Disease3" is hyperlipidemia, "Disease4" is heat disease, "Disease 5" is kindey disease,, and "Disease 6" is other diseases. "ApoE4 hetero" and "ApoE4 homo" are dummy variables indicating carriers of the ApoE4 heterozygous and homozygous genotypes, respectively.

Table S2 (continued). Regression results (Albumin)

Albumin	Males						Females					
	Coef.	S.E.	t	P(t)	[95%	C.I.]	Coef.	S.E.	t	P(t)	[95%	C.I.]
1[Age<65] × Dietary 1	4.535	0.055	82.79	0.000	4.427	4.642	4.524	0.038	118.53	0.000	4.450	4.599
1[Age<65] × Dietary 2	4.440	0.057	77.67	0.000	4.328	4.552	4.514	0.041	110.41	0.000	4.434	4.595
1[Age<65] × Dietary 3	4.533	0.060	75.67	0.000	4.415	4.650	4.534	0.039	116.50	0.000	4.458	4.610
1[Age<65] × Dietary 4	4.433	0.072	62.04	0.000	4.293	4.573	4.517	0.043	104.45	0.000	4.432	4.601
1[Age<65] × Dietary 5	4.512	0.055	82.27	0.000	4.405	4.620	4.542	0.037	124.09	0.000	4.470	4.613
1[Age<65] × Dietary 6	4.404	0.081	54.32	0.000	4.245	4.563	4.558	0.045	100.33	0.000	4.468	4.647
1[Age≥65] × Dietary 1	4.474	0.052	86.46	0.000	4.372	4.576	4.435	0.044	100.12	0.000	4.348	4.522
1[Age≥65] × Dietary 2	4.466	0.052	86.38	0.000	4.364	4.567	4.468	0.044	101.83	0.000	4.382	4.554
1[Age≥65] × Dietary 3	4.443	0.053	84.13	0.000	4.340	4.547	4.483	0.040	110.97	0.000	4.404	4.563
1[Age≥65] × Dietary 4	4.482	0.054	83.48	0.000	4.377	4.588	4.475	0.039	114.80	0.000	4.399	4.551
1[Age≥65] × Dietary 5	4.501	0.051	88.06	0.000	4.401	4.602	4.496	0.037	121.37	0.000	4.424	4.569
1[Age≥65] × Dietary 6	4.479	0.060	74.98	0.000	4.362	4.596	4.460	0.042	107.17	0.000	4.378	4.542
School	0.000	0.026	0.017	0.987	-0.050	0.051	-0.028	0.018	-1.563	0.118	-0.064	0.007
Non cohabitants	-0.041	0.046	-0.883	0.378	-0.132	0.050	-0.024	0.025	-0.936	0.350	-0.073	0.026
Lifestyle change	0.019	0.017	1.122	0.262	-0.015	0.053	-0.003	0.013	-0.217	0.828	-0.027	0.022
Paid	0.012	0.024	0.504	0.615	-0.034	0.058	-0.026	0.017	-1.540	0.124	-0.060	0.007
Well off	-0.001	0.022	-0.028	0.978	-0.045	0.043	-0.007	0.015	-0.450	0.653	-0.037	0.023
Alcohol	0.001	0.024	0.031	0.975	-0.046	0.048	0.034	0.016	2.139	0.033	0.003	0.065
Smoking	-0.053	0.035	-1.506	0.132	-0.122	0.016	-0.004	0.038	-0.118	0.906	-0.079	0.070
Healthy 1	-0.021	0.041	-0.507	0.612	-0.101	0.059	-0.023	0.031	-0.749	0.454	-0.083	0.037
Healthy 2	-0.047	0.030	-1.556	0.120	-0.105	0.012	0.006	0.024	0.236	0.813	-0.042	0.053
Exercise 1	-0.011	0.026	-0.406	0.685	-0.062	0.041	-0.022	0.019	-1.169	0.243	-0.058	0.015
Exercise 2	-0.022	0.022	-0.990	0.323	-0.065	0.021	-0.038	0.015	-2.503	0.012	-0.068	-0.008
Disease 1	-0.005	0.024	-0.220	0.826	-0.051	0.041	0.045	0.021	2.133	0.033	0.004	0.087
Disease 2	0.150	0.035	4.299	0.000	0.081	0.218	0.006	0.047	0.128	0.898	-0.086	0.099
Disease 3	0.034	0.029	1.159	0.247	-0.023	0.091	0.007	0.021	0.337	0.736	-0.034	0.049
Disease 4	0.002	0.039	0.053	0.958	-0.074	0.078	-0.007	0.057	-0.127	0.899	-0.119	0.105
Disease 5	-0.037	0.031	-1.209	0.227	-0.098	0.023	-0.117	0.080	-1.464	0.143	-0.274	0.040
disease 6	-0.032	0.019	-1.715	0.087	-0.069	0.005	-0.018	0.015	-1.234	0.218	-0.047	0.011
ApoE4 hetero	-0.038	0.028	-1.354	0.176	-0.092	0.017	0.021	0.022	0.933	0.351	-0.023	0.065
ApoE4 homo	0.088	0.148	0.591	0.554	-0.203	0.378	0.158	0.074	2.149	0.032	0.014	0.302
Y2024	-0.086	0.011	-7.817	0.000	-0.108	-0.065	-0.083	0.009	-9.788	0.000	-0.100	-0.066
	N		915	F		8.000	N		1424	F		18.912
	R2 (Between)		0.099	P(F)		0.000	R2 (Between)		0.071	P(F)		0.000
	R2 (Within)		0.136	σ2(u)		0.050	R2 (Within)		0.149	σ2(u)		0.044
	R2 (Overall)		0.104	σ2(ε)		0.026	R2 (Overall)		0.086	σ2(ε)		0.023
	Log-likelihood		383.5	σ2(u)/σ2(u+ε)		0.659	Log-likelihood		663.5	σ2(u)/σ2(u+ε)		0.654

Note: "Dietary" denotes the dietary habit cluster: "Dietary 1" is a dummy variable indicating whether an individual belongs to dietary habit cluster 1, and so on. "School": an individual has 13 or more years of education; "Non cohabitants": the individual lives alone; "Lifestyle change": the individual experienced a major change in lifestyle during the past year; "Paid": the individual is engaged in paid work; "Well off": the household is financially comfortable; "Alcohol": the individual has a drinking habit; "Smoking": the individual has a smoking habit; "Health 1 (or 2)": the subjective health status is "very" (or "fairly") healthy; "Exercise1": the individual engages in light exercise at least once per week; "Exercise2": the individual engages in moderate or vigorous exercise at least once per week; "Y 2024": that the survey year is 2024. "Disease1" through "Disease6" are indicators for diseases under treatment or with lasting effects: "Disease1" is hypertension, "Disease2" is diabetes, "Disease3" is hyperlipidemia, "Disease4" is heat disease, "Disease 5" is kindey disease,, and "Disease 6" is other diseases. "ApoE4 hetero" and "ApoE4 homo" are dummy variables indicating carriers of the ApoE4 heterozygous and homozygous genotypes, respectively.

Table S2 (continued). Regression results (AST)

AST	Males						Females					
	Coef.	S.E.	t	P(t)	[95%	C.I.]	Coef.	S.E.	t	P(t)	[95%	C.I.]
1[Age<65] × Dietary 1	22.38	1.914	11.69	0.000	18.62	26.14	25.19	1.049	24.02	0.000	23.13	27.25
1[Age<65] × Dietary 2	21.16	2.012	10.51	0.000	17.21	25.11	26.24	1.124	23.36	0.000	24.04	28.44
1[Age<65] × Dietary 3	21.01	2.119	9.915	0.000	16.85	25.17	26.03	1.070	24.34	0.000	23.93	28.13
1[Age<65] × Dietary 4	19.05	2.542	7.495	0.000	14.06	24.04	25.22	1.189	21.22	0.000	22.89	27.55
1[Age<65] × Dietary 5	21.65	1.932	11.21	0.000	17.86	25.44	24.80	1.006	24.66	0.000	22.83	26.77
1[Age<65] × Dietary 6	18.09	2.891	6.259	0.000	12.42	23.77	24.65	1.248	19.75	0.000	22.20	27.10
1[Age≥65] × Dietary 1	23.92	1.821	13.13	0.000	20.34	27.49	24.11	1.217	19.80	0.000	21.72	26.50
1[Age≥65] × Dietary 2	21.90	1.820	12.03	0.000	18.33	25.47	25.13	1.205	20.85	0.000	22.76	27.49
1[Age≥65] × Dietary 3	22.47	1.859	12.09	0.000	18.82	26.12	25.36	1.110	22.84	0.000	23.18	27.54
1[Age≥65] × Dietary 4	26.21	1.896	13.83	0.000	22.49	29.93	24.61	1.071	22.97	0.000	22.51	26.71
1[Age≥65] × Dietary 5	22.87	1.803	12.68	0.000	19.33	26.40	24.55	1.018	24.12	0.000	22.55	26.55
1[Age≥65] × Dietary 6	22.17	2.137	10.38	0.000	17.98	26.37	24.44	1.144	21.37	0.000	22.19	26.68
School	0.091	0.847	0.108	0.914	-1.571	1.753	-0.094	0.496	-0.190	0.850	-1.068	0.880
Non cohabitants	2.436	1.575	1.546	0.122	-0.656	5.527	-0.075	0.695	-0.108	0.914	-1.438	1.289
Lifestyle change	-0.315	0.639	-0.493	0.622	-1.568	0.939	-0.197	0.345	-0.571	0.568	-0.873	0.480
Paid	1.532	0.815	1.880	0.060	-0.067	3.131	-0.681	0.468	-1.455	0.146	-1.599	0.237
Well off	0.584	0.791	0.738	0.461	-0.969	2.137	-0.077	0.424	-0.182	0.856	-0.909	0.755
Alcohol	1.356	0.811	1.671	0.095	-0.237	2.949	0.263	0.433	0.608	0.543	-0.586	1.112
Smoking	-1.491	1.175	-1.269	0.205	-3.798	0.816	0.104	1.038	0.100	0.920	-1.933	2.141
Healthy 1	0.158	1.487	0.107	0.915	-2.760	3.077	0.283	0.846	0.335	0.738	-1.376	1.942
Healthy 2	1.441	1.096	1.315	0.189	-0.710	3.592	0.062	0.661	0.094	0.925	-1.233	1.358
Exercise 1	-1.021	0.963	-1.060	0.289	-2.911	0.869	0.157	0.510	0.308	0.758	-0.843	1.158
Exercise 2	0.886	0.798	1.111	0.267	-0.680	2.452	-0.498	0.420	-1.184	0.237	-1.323	0.327
Disease 1	0.334	0.817	0.409	0.683	-1.269	1.937	-0.383	0.584	-0.656	0.512	-1.528	0.762
Disease 2	-0.818	1.191	-0.687	0.492	-3.156	1.520	0.583	1.294	0.450	0.653	-1.956	3.122
Disease 3	0.284	1.016	0.279	0.780	-1.710	2.278	1.648	0.581	2.835	0.005	0.508	2.788
Disease 4	-0.291	1.375	-0.212	0.832	-2.989	2.407	3.633	1.570	2.313	0.021	0.552	6.713
Disease 5	-0.378	1.121	-0.337	0.736	-2.579	1.823	3.457	2.203	1.570	0.117	-0.864	7.777
disease 6	0.141	0.682	0.207	0.836	-1.196	1.479	-0.009	0.406	-0.023	0.982	-0.806	0.788
ApoE4 hetero	-0.118	0.908	-0.130	0.897	-1.900	1.664	-0.836	0.611	-1.367	0.172	-2.035	0.363
ApoE4 homo	6.173	4.840	1.275	0.203	-3.327	15.67	-1.966	2.016	-0.975	0.330	-5.921	1.989
Y2024	-0.100	0.433	-0.231	0.818	-0.950	0.750	-0.494	0.234	-2.112	0.035	-0.953	-0.035
	N		915	F		1.356	N		1424	F		1.945
	R2 (Between)		0.012	P(F)		0.094	R2 (Between)		0.040	P(F)		0.002
	R2 (Within)		0.074	σ2(u)		46.16	R2 (Within)		0.016	σ2(u)		32.91
	R2 (Overall)		0.028	σ2(ε)		39.72	R2 (Overall)		0.033	σ2(ε)		17.66
	Log-likelihood		-2978.7	σ2(u)/σ2(u+ε)		0.537	Log-likelihood		-4059.0	σ2(u)/σ2(u+ε)		0.651

Note: "Dietary" denotes the dietary habit cluster: "Dietary 1" is a dummy variable indicating whether an individual belongs to dietary habit cluster 1, and so on. "School": an individual has 13 or more years of education; "Non cohabitants": the individual lives alone; "Lifestyle change": the individual experienced a major change in lifestyle during the past year; "Paid": the individual is engaged in paid work; "Well off": the household is financially comfortable; "Alcohol": the individual has a drinking habit; "Smoking": the individual has a smoking habit; "Health 1 (or 2)": the subjective health status is "very" (or "fairly") healthy; "Exercise1": the individual engages in light exercise at least once per week; "Exercise2": the individual engages in moderate or vigorous exercise at least once per week; "Y 2024": that the survey year is 2024. "Disease1" through "Disease6" are indicators for diseases under treatment or with lasting effects: "Disease1" is hypertension, "Disease2" is diabetes, "Disease3" is hyperlipidemia, "Disease4" is heat disease, "Disease 5" is kindey disease,, and "Disease 6" is other diseases. "ApoE4 hetero" and "ApoE4 homo" are dummy variables indicating carriers of the ApoE4 heterozygous and homozygous genotypes, respectively.

Table S2 (continued). Regression results (ALT)

ALT	Males						Females					
	Coef.	S.E.	t	P(t)	[95%	C.I.]	Coef.	S.E.	t	P(t)	[95%	C.I.]
1[Age<65] × Dietary 1	24.60	2.206	11.15	0.000	20.27	28.93	24.31	1.639	14.83	0.000	21.09	27.52
1[Age<65] × Dietary 2	21.30	2.294	9.283	0.000	16.79	25.80	25.80	1.753	14.71	0.000	22.36	29.24
1[Age<65] × Dietary 3	22.06	2.396	9.206	0.000	17.36	26.76	26.35	1.666	15.82	0.000	23.08	29.62
1[Age<65] × Dietary 4	25.00	2.847	8.780	0.000	19.41	30.59	23.85	1.847	12.91	0.000	20.22	27.47
1[Age<65] × Dietary 5	22.88	2.200	10.40	0.000	18.56	27.19	24.00	1.570	15.29	0.000	20.92	27.08
1[Age<65] × Dietary 6	17.18	3.223	5.331	0.000	10.86	23.51	23.68	1.945	12.18	0.000	19.86	27.49
1[Age≥65] × Dietary 1	24.51	2.077	11.80	0.000	20.43	28.59	22.86	1.895	12.07	0.000	19.14	26.58
1[Age≥65] × Dietary 2	20.58	2.074	9.925	0.000	16.51	24.65	23.31	1.886	12.36	0.000	19.61	27.01
1[Age≥65] × Dietary 3	21.66	2.120	10.22	0.000	17.50	25.82	23.91	1.728	13.84	0.000	20.52	27.30
1[Age≥65] × Dietary 4	23.76	2.152	11.04	0.000	19.53	27.98	22.10	1.671	13.23	0.000	18.82	25.38
1[Age≥65] × Dietary 5	22.82	2.051	11.13	0.000	18.80	26.85	22.37	1.588	14.09	0.000	19.25	25.48
1[Age≥65] × Dietary 6	21.77	2.376	9.164	0.000	17.11	26.44	21.49	1.783	12.05	0.000	18.00	24.99
School	0.835	1.085	0.769	0.442	-1.295	2.965	-0.262	0.799	-0.328	0.743	-1.830	1.306
Non cohabitants	0.721	1.885	0.383	0.702	-2.979	4.422	-1.161	1.101	-1.055	0.292	-3.322	0.999
Lifestyle change	0.136	0.671	0.202	0.840	-1.181	1.453	-0.368	0.528	-0.696	0.487	-1.404	0.669
Paid	1.063	0.949	1.120	0.263	-0.799	2.925	-0.807	0.736	-1.096	0.273	-2.251	0.638
Well off	-0.644	0.891	-0.723	0.470	-2.392	1.104	-0.098	0.659	-0.149	0.882	-1.390	1.195
Alcohol	0.332	0.977	0.340	0.734	-1.586	2.250	-0.185	0.682	-0.271	0.787	-1.522	1.153
Smoking	-0.792	1.446	-0.548	0.584	-3.630	2.046	-0.081	1.651	-0.049	0.961	-3.320	3.157
Healthy 1	0.148	1.602	0.093	0.926	-2.996	3.292	-0.454	1.309	-0.347	0.729	-3.021	2.114
Healthy 2	2.797	1.173	2.384	0.017	0.494	5.099	-0.445	1.023	-0.435	0.664	-2.452	1.562
Exercise 1	-1.476	1.029	-1.434	0.152	-3.496	0.545	-0.210	0.787	-0.267	0.790	-1.753	1.334
Exercise 2	0.372	0.865	0.430	0.667	-1.326	2.069	-0.300	0.650	-0.461	0.645	-1.575	0.976
Disease 1	-0.225	0.948	-0.237	0.813	-2.085	1.636	-0.328	0.916	-0.358	0.720	-2.124	1.469
Disease 2	0.538	1.419	0.379	0.705	-2.247	3.324	5.627	2.036	2.764	0.006	1.633	9.621
Disease 3	2.170	1.163	1.867	0.062	-0.112	4.452	2.365	0.909	2.601	0.009	0.581	4.148
Disease 4	-0.785	1.532	-0.512	0.609	-3.790	2.221	7.149	2.468	2.897	0.004	2.308	11.99
Disease 5	-0.403	1.219	-0.331	0.741	-2.795	1.989	0.563	3.388	0.166	0.868	-6.084	7.210
disease 6	-0.346	0.736	-0.470	0.639	-1.791	1.099	-0.358	0.629	-0.569	0.570	-1.592	0.876
ApoE4 hetero	-1.037	1.163	-0.892	0.373	-3.320	1.246	-0.887	0.985	-0.900	0.368	-2.819	1.046
ApoE4 homo	4.641	6.220	0.746	0.456	-7.567	16.85	-1.444	3.247	-0.445	0.657	-7.814	4.926
Y2024	-0.913	0.419	-2.180	0.030	-1.734	-0.091	-1.040	0.352	-2.952	0.003	-1.731	-0.349
	N		915	F		1.612	N		1424	F		2.375
	R2 (Between)		0.051	P(F)		0.019	R2 (Between)		0.062	P(F)		0.000
	R2 (Within)		0.056	σ2(u)		92.47	R2 (Within)		0.025	σ2(u)		88.10
	R2 (Overall)		0.052	σ2(ε)		36.92	R2 (Overall)		0.052	σ2(ε)		39.62
	Log-likelihood		-2939.9	σ2(u)/σ2(u+ε)		0.715	Log-likelihood		-4638.9	σ2(u)/σ2(u+ε)		0.690

Note: "Dietary" denotes the dietary habit cluster: "Dietary 1" is a dummy variable indicating whether an individual belongs to dietary habit cluster 1, and so on. "School": an individual has 13 or more years of education; "Non cohabitants": the individual lives alone; "Lifestyle change": the individual experienced a major change in lifestyle during the past year; "Paid": the individual is engaged in paid work; "Well off": the household is financially comfortable; "Alcohol": the individual has a drinking habit; "Smoking": the individual has a smoking habit; "Health 1 (or 2)": the subjective health status is "very" (or "fairly") healthy; "Exercise1": the individual engages in light exercise at least once per week; "Exercise2": the individual engages in moderate or vigorous exercise at least once per week; "Y 2024": that the survey year is 2024. "Disease1" through "Disease6" are indicators for diseases under treatment or with lasting effects: "Disease1" is hypertension, "Disease2" is diabetes, "Disease3" is hyperlipidemia, "Disease4" is heat disease, "Disease 5" is kindey disease,, and "Disease 6" is other diseases. "ApoE4 hetero" and "ApoE4 homo" are dummy variables indicating carriers of the ApoE4 heterozygous and homozygous genotypes, respectively.

Table S2 (continued). Regression results (LDH)

LDH	Males						Females					
	Coef.	S.E.	t	P(t)	[95%	C.I.]	Coef.	S.E.	t	P(t)	[95%	C.I.]
1[Age<65] × Dietary 1	189.15	6.259	30.22	0.000	176.87	201.44	194.58	3.999	48.66	0.000	186.74	202.43
1[Age<65] × Dietary 2	186.61	6.507	28.68	0.000	173.84	199.38	194.77	4.262	45.71	0.000	186.41	203.13
1[Age<65] × Dietary 3	179.47	6.794	26.42	0.000	166.14	192.81	196.00	4.033	48.60	0.000	188.09	203.91
1[Age<65] × Dietary 4	187.14	8.071	23.19	0.000	171.30	202.98	197.50	4.443	44.46	0.000	188.79	206.22
1[Age<65] × Dietary 5	188.04	6.240	30.14	0.000	175.79	200.28	196.87	3.824	51.48	0.000	189.37	204.37
1[Age<65] × Dietary 6	180.65	9.136	19.78	0.000	162.72	198.58	200.32	4.703	42.60	0.000	191.10	209.55
1[Age≥65] × Dietary 1	191.29	5.892	32.47	0.000	179.73	202.86	197.01	4.574	43.07	0.000	188.03	205.98
1[Age≥65] × Dietary 2	187.65	5.882	31.90	0.000	176.10	199.19	200.35	4.607	43.49	0.000	191.31	209.38
1[Age≥65] × Dietary 3	187.19	6.012	31.14	0.000	175.39	198.99	199.76	4.174	47.86	0.000	191.58	207.95
1[Age≥65] × Dietary 4	195.84	6.102	32.09	0.000	183.87	207.82	204.45	4.056	50.41	0.000	196.49	212.41
1[Age≥65] × Dietary 5	195.00	5.816	33.53	0.000	183.58	206.41	201.36	3.856	52.22	0.000	193.80	208.93
1[Age≥65] × Dietary 6	188.86	6.735	28.04	0.000	175.64	202.08	208.88	4.323	48.32	0.000	200.40	217.36
School	-4.332	3.088	-1.403	0.161	-10.39	1.729	-3.415	2.102	-1.624	0.105	-7.539	0.710
Non cohabitants	4.957	5.354	0.926	0.355	-5.550	15.46	1.929	2.770	0.697	0.486	-3.504	7.362
Lifestyle change	0.951	1.899	0.501	0.617	-2.776	4.678	1.615	1.230	1.313	0.190	-0.798	4.029
Paid	0.327	2.692	0.121	0.903	-4.956	5.610	2.928	1.818	1.610	0.108	-0.639	6.495
Well off	-2.885	2.525	-1.143	0.254	-7.841	2.070	-0.315	1.580	-0.200	0.842	-3.414	2.783
Alcohol	-0.993	2.775	-0.358	0.721	-6.440	4.454	-0.236	1.690	-0.140	0.889	-3.551	3.079
Smoking	-1.152	4.109	-0.280	0.779	-9.216	6.913	-0.598	4.191	-0.143	0.887	-8.820	7.623
Healthy 1	6.586	4.535	1.452	0.147	-2.315	15.49	2.443	3.112	0.785	0.433	-3.661	8.547
Healthy 2	1.824	3.321	0.549	0.583	-4.694	8.343	3.318	2.437	1.361	0.174	-1.463	8.098
Exercise 1	3.778	2.914	1.296	0.195	-1.942	9.498	0.809	1.858	0.436	0.663	-2.835	4.453
Exercise 2	0.837	2.449	0.342	0.733	-3.970	5.645	-0.460	1.546	-0.297	0.766	-3.492	2.573
Disease 1	2.523	2.689	0.938	0.349	-2.756	7.801	0.373	2.246	0.166	0.868	-4.033	4.779
Disease 2	-3.936	4.030	-0.977	0.329	-11.84	3.973	-7.642	5.028	-1.520	0.129	-17.51	2.221
Disease 3	2.371	3.297	0.719	0.472	-4.100	8.842	1.796	2.212	0.812	0.417	-2.544	6.136
Disease 4	1.458	4.340	0.336	0.737	-7.060	9.975	0.694	6.078	0.114	0.909	-11.23	12.62
Disease 5	-0.112	3.452	-0.032	0.974	-6.886	6.662	-12.14	7.950	-1.526	0.127	-27.73	3.461
disease 6	0.996	2.084	0.478	0.633	-3.095	5.086	-0.896	1.496	-0.599	0.549	-3.831	2.039
ApoE4 hetero	0.431	3.310	0.130	0.897	-6.065	6.927	0.676	2.595	0.261	0.794	-4.414	5.766
ApoE4 homo	22.40	17.70	1.265	0.206	-12.34	57.14	9.025	8.544	1.056	0.291	-7.735	25.79
Y2024	5.469	1.183	4.625	0.000	3.148	7.790	6.767	0.793	8.529	0.000	5.211	8.324
	N		915	F		2.403	N		1424	F		6.856
	R2 (Between)		0.032	P(F)		0.000	R2 (Between)		0.061	P(F)		0.000
	R2 (Within)		0.094	σ2(u)		759.25	R2 (Within)		0.098	σ2(u)		652.34
	R2 (Overall)		0.042	σ2(ε)		297.59	R2 (Overall)		0.065	σ2(ε)		198.69
	Log-likelihood		-3889.9	σ2(u)/σ2(u+ε)		0.718	Log-likelihood		-5786.7	σ2(u)/σ2(u+ε)		0.767

Note: "Dietary" denotes the dietary habit cluster: "Dietary 1" is a dummy variable indicating whether an individual belongs to dietary habit cluster 1, and so on. "School": an individual has 13 or more years of education; "Non cohabitants": the individual lives alone; "Lifestyle change": the individual experienced a major change in lifestyle during the past year; "Paid": the individual is engaged in paid work; "Well off": the household is financially comfortable; "Alcohol": the individual has a drinking habit; "Smoking": the individual has a smoking habit; "Health 1 (or 2)": the subjective health status is "very" (or "fairly") healthy; "Exercise1": the individual engages in light exercise at least once per week; "Exercise2": the individual engages in moderate or vigorous exercise at least once per week; "Y 2024": that the survey year is 2024. "Disease1" through "Disease6" are indicators for diseases under treatment or with lasting effects: "Disease1" is hypertension, "Disease2" is diabetes, "Disease3" is hyperlipidemia, "Disease4" is heat disease, "Disease 5" is kindey disease,, and "Disease 6" is other diseases. "ApoE4 hetero" and "ApoE4 homo" are dummy variables indicating carriers of the ApoE4 heterozygous and homozygous genotypes, respectively.

Table S2 (continued). Regression results (ALP)

ALP	Males						Females					
	Coef.	S.E.	t	P(t)	[95%	C.I.]	Coef.	S.E.	t	P(t)	[95%	C.I.]
1[Age<65] × Dietary 1	76.44	3.168	24.13	0.000	70.22	82.65	84.57	3.543	23.87	0.000	77.62	91.52
1[Age<65] × Dietary 2	72.87	3.245	22.46	0.000	66.50	79.23	82.40	3.793	21.72	0.000	74.96	89.84
1[Age<65] × Dietary 3	74.84	3.316	22.57	0.000	68.34	81.35	85.26	3.610	23.62	0.000	78.18	92.34
1[Age<65] × Dietary 4	76.20	3.816	19.97	0.000	68.71	83.69	82.14	4.009	20.49	0.000	74.28	90.00
1[Age<65] × Dietary 5	74.27	3.109	23.89	0.000	68.17	80.37	82.75	3.396	24.37	0.000	76.09	89.42
1[Age<65] × Dietary 6	73.65	4.236	17.39	0.000	65.34	81.97	82.10	4.213	19.49	0.000	73.84	90.37
1[Age≥65] × Dietary 1	74.06	2.963	24.99	0.000	68.25	79.88	89.30	4.108	21.74	0.000	81.24	97.36
1[Age≥65] × Dietary 2	74.61	2.943	25.36	0.000	68.84	80.39	84.01	4.073	20.63	0.000	76.02	92.00
1[Age≥65] × Dietary 3	76.56	3.010	25.43	0.000	70.65	82.46	85.66	3.746	22.87	0.000	78.31	93.01
1[Age≥65] × Dietary 4	75.51	3.041	24.83	0.000	69.54	81.48	83.74	3.616	23.16	0.000	76.64	90.83
1[Age≥65] × Dietary 5	73.69	2.923	25.21	0.000	67.96	79.43	83.34	3.437	24.25	0.000	76.60	90.08
1[Age≥65] × Dietary 6	76.71	3.229	23.76	0.000	70.37	83.05	83.17	3.861	21.54	0.000	75.60	90.74
School	0.154	2.019	0.077	0.939	-3.809	4.118	0.655	1.689	0.388	0.698	-2.658	3.969
Non cohabitants	-4.451	2.789	-1.596	0.111	-9.925	1.022	-1.619	2.356	-0.687	0.492	-6.241	3.002
Lifestyle change	1.498	0.780	1.919	0.055	-0.034	3.029	-0.783	1.159	-0.676	0.499	-3.056	1.490
Paid	-0.426	1.304	-0.327	0.744	-2.985	2.134	-0.068	1.584	-0.043	0.966	-3.174	3.039
Well off	-1.343	1.146	-1.171	0.242	-3.592	0.907	0.286	1.430	0.200	0.842	-2.520	3.092
Alcohol	-2.382	1.458	-1.634	0.103	-5.243	0.478	-2.928	1.464	-2.000	0.046	-5.801	-0.056
Smoking	3.675	2.296	1.601	0.110	-0.831	8.181	-0.190	3.522	-0.054	0.957	-7.099	6.720
Healthy 1	-2.189	1.928	-1.135	0.257	-5.973	1.596	0.453	2.850	0.159	0.874	-5.137	6.042
Healthy 2	1.147	1.397	0.821	0.412	-1.595	3.890	0.854	2.226	0.384	0.701	-3.513	5.221
Exercise 1	-0.203	1.227	-0.165	0.869	-2.611	2.206	-1.425	1.717	-0.830	0.407	-4.794	1.943
Exercise 2	1.229	1.052	1.169	0.243	-0.835	3.294	0.625	1.417	0.441	0.659	-2.154	3.404
Disease 1	0.284	1.295	0.219	0.827	-2.258	2.826	-0.189	1.974	-0.096	0.924	-4.061	3.683
Disease 2	-0.972	2.053	-0.473	0.636	-5.002	3.058	2.500	4.379	0.571	0.568	-6.091	11.09
Disease 3	-2.319	1.538	-1.507	0.132	-5.337	0.700	-2.382	1.964	-1.213	0.226	-6.234	1.471
Disease 4	0.485	1.938	0.250	0.802	-3.319	4.289	8.523	5.312	1.604	0.109	-1.898	18.94
Disease 5	0.151	1.476	0.102	0.919	-2.747	3.049	0.427	7.410	0.058	0.954	-14.11	14.96
disease 6	0.417	0.886	0.471	0.638	-1.322	2.157	-1.165	1.369	-0.851	0.395	-3.851	1.521
ApoE4 hetero	-4.200	2.163	-1.942	0.053	-8.445	0.045	-5.313	2.081	-2.553	0.011	-9.394	-1.231
ApoE4 homo	45.63	11.62	3.926	0.000	22.82	68.44	-5.180	6.861	-0.755	0.450	-18.64	8.279
Y2024	0.632	0.448	1.411	0.159	-0.247	1.510	-0.100	0.783	-0.127	0.899	-1.635	1.436
	N		915	F		2.073	N		1424	F		1.323
	R2 (Between)		0.074	P(F)		0.001	R2 (Between)		0.031	P(F)		0.111
	R2 (Within)		0.046	σ2(u)		376.93	R2 (Within)		0.004	σ2(u)		392.05
	R2 (Overall)		0.073	σ2(ε)		41.40	R2 (Overall)		0.027	σ2(ε)		201.04
	Log-likelihood		-2985.0	σ2(u)/σ2(u+ε)		0.901	Log-likelihood		-5777.9	σ2(u)/σ2(u+ε)		0.661

Note: "Dietary" denotes the dietary habit cluster: "Dietary 1" is a dummy variable indicating whether an individual belongs to dietary habit cluster 1, and so on. "School": an individual has 13 or more years of education; "Non cohabitants": the individual lives alone; "Lifestyle change": the individual experienced a major change in lifestyle during the past year; "Paid": the individual is engaged in paid work; "Well off": the household is financially comfortable; "Alcohol": the individual has a drinking habit; "Smoking": the individual has a smoking habit; "Health 1 (or 2)": the subjective health status is "very" (or "fairly") healthy; "Exercise1": the individual engages in light exercise at least once per week; "Exercise2": the individual engages in moderate or vigorous exercise at least once per week; "Y 2024": that the survey year is 2024. "Disease1" through "Disease6" are indicators for diseases under treatment or with lasting effects: "Disease1" is hypertension, "Disease2" is diabetes, "Disease3" is hyperlipidemia, "Disease4" is heat disease, "Disease 5" is kindey disease,, and "Disease 6" is other diseases. "ApoE4 hetero" and "ApoE4 homo" are dummy variables indicating carriers of the ApoE4 heterozygous and homozygous genotypes, respectively.

Table S2 (continued). Regression results (γ -GTP)

γ-GTP	Males						Females					
	Coef.	S.E.	t	P(t)	[95%	C.I.]	Coef.	S.E.	t	P(t)	[95%	C.I.]
1[Age<65] × Dietary 1	31.61	7.464	4.235	0.000	16.96	46.26	25.38	3.699	6.860	0.000	18.12	32.63
1[Age<65] × Dietary 2	27.57	7.677	3.591	0.000	12.50	42.64	26.16	3.957	6.612	0.000	18.40	33.93
1[Age<65] × Dietary 3	33.40	7.903	4.226	0.000	17.89	48.91	30.26	3.761	8.046	0.000	22.88	37.64
1[Age<65] × Dietary 4	32.32	9.213	3.508	0.001	14.24	50.40	28.28	4.170	6.782	0.000	20.10	36.46
1[Age<65] × Dietary 5	35.73	7.353	4.860	0.000	21.30	50.17	27.92	3.544	7.877	0.000	20.96	34.87
1[Age<65] × Dietary 6	28.73	10.31	2.787	0.005	8.497	48.96	28.31	4.390	6.447	0.000	19.69	36.92
1[Age≥65] × Dietary 1	36.42	6.980	5.218	0.000	22.72	50.12	26.82	4.278	6.270	0.000	18.43	35.21
1[Age≥65] × Dietary 2	37.16	6.942	5.353	0.000	23.53	50.79	27.68	4.256	6.506	0.000	19.34	36.03
1[Age≥65] × Dietary 3	32.83	7.104	4.622	0.000	18.89	46.77	27.77	3.902	7.119	0.000	20.12	35.43
1[Age≥65] × Dietary 4	34.04	7.184	4.738	0.000	19.94	48.14	24.04	3.772	6.374	0.000	16.64	31.44
1[Age≥65] × Dietary 5	28.97	6.881	4.210	0.000	15.47	42.48	24.50	3.584	6.836	0.000	17.47	31.53
1[Age≥65] × Dietary 6	31.29	7.723	4.051	0.000	16.13	46.44	21.70	4.025	5.391	0.000	13.81	29.60
School	-0.378	4.338	-0.087	0.931	-8.892	8.136	0.578	1.798	0.321	0.748	-2.948	4.104
Non cohabitants	-4.416	6.578	-0.671	0.502	-17.33	8.494	-4.606	2.481	-1.856	0.064	-9.474	0.261
Lifestyle change	4.680	1.981	2.362	0.018	0.791	8.568	-0.667	1.195	-0.558	0.577	-3.011	1.677
Paid	0.789	3.156	0.250	0.803	-5.405	6.983	-0.986	1.660	-0.594	0.553	-4.243	2.271
Well off	4.500	2.832	1.589	0.112	-1.057	10.06	2.007	1.488	1.349	0.178	-0.912	4.926
Alcohol	14.78	3.431	4.307	0.000	8.042	21.51	1.245	1.537	0.810	0.418	-1.770	4.260
Smoking	2.737	5.279	0.519	0.604	-7.623	13.10	4.980	3.718	1.339	0.181	-2.315	12.27
Healthy 1	-3.893	4.851	-0.803	0.423	-13.41	5.628	3.551	2.957	1.201	0.230	-2.250	9.352
Healthy 2	-0.574	3.526	-0.163	0.871	-7.495	6.347	3.540	2.312	1.531	0.126	-0.995	8.074
Exercise 1	-1.149	3.095	-0.371	0.711	-7.224	4.926	-0.754	1.779	-0.424	0.672	-4.243	2.735
Exercise 2	-3.707	2.639	-1.405	0.160	-8.885	1.472	1.813	1.470	1.234	0.218	-1.070	4.696
Disease 1	6.114	3.141	1.947	0.052	-0.050	12.28	2.246	2.066	1.087	0.277	-1.807	6.299
Disease 2	-4.292	4.887	-0.878	0.380	-13.88	5.299	0.245	4.591	0.053	0.958	-8.762	9.251
Disease 3	-0.942	3.769	-0.250	0.803	-8.339	6.456	-0.604	2.052	-0.295	0.768	-4.629	3.420
Disease 4	-0.693	4.810	-0.144	0.886	-10.13	8.747	5.920	5.566	1.064	0.288	-5.000	16.84
Disease 5	-2.133	3.712	-0.575	0.566	-9.418	5.152	3.860	7.662	0.504	0.615	-11.17	18.89
disease 6	1.755	2.230	0.787	0.432	-2.623	6.133	0.047	1.421	0.033	0.973	-2.741	2.835
ApoE4 hetero	4.626	4.647	0.995	0.320	-4.495	13.75	-2.655	2.215	-1.198	0.231	-7.000	1.691
ApoE4 homo	70.05	24.94	2.809	0.005	21.11	118.99	-1.217	7.302	-0.167	0.868	-15.54	13.11
Y2024	-2.888	1.159	-2.492	0.013	-5.163	-0.614	-2.316	0.799	-2.900	0.004	-3.883	-0.750
	N		915	F		1.994	N		1424	F		1.335
	R2 (Between)		0.095	P(F)		0.001	R2 (Between)		0.033	P(F)		0.104
	R2 (Within)		0.034	σ2(u)		1659.47	R2 (Within)		0.019	σ2(u)		452.56
	R2 (Overall)		0.091	σ2(ε)		276.41	R2 (Overall)		0.032	σ2(ε)		207.77
	Log-likelihood		-3860.6	σ2(u)/σ2(u+ε)		0.857	Log-likelihood		-5804.5	σ2(u)/σ2(u+ε)		0.685

Note: "Dietary" denotes the dietary habit cluster: "Dietary 1" is a dummy variable indicating whether an individual belongs to dietary habit cluster 1, and so on. "School": an individual has 13 or more years of education; "Non cohabitants": the individual lives alone; "Lifestyle change": the individual experienced a major change in lifestyle during the past year; "Paid": the individual is engaged in paid work; "Well off": the household is financially comfortable; "Alcohol": the individual has a drinking habit; "Smoking": the individual has a smoking habit; "Health 1 (or 2)": the subjective health status is "very" (or "fairly") healthy; "Exercise1": the individual engages in light exercise at least once per week; "Exercise2": the individual engages in moderate or vigorous exercise at least once per week; "Y 2024": that the survey year is 2024. "Disease1" through "Disease6" are indicators for diseases under treatment or with lasting effects: "Disease1" is hypertension, "Disease2" is diabetes, "Disease3" is hyperlipidemia, "Disease4" is heat disease, "Disease 5" is kindey disease,, and "Disease 6" is other diseases. "ApoE4 hetero" and "ApoE4 homo" are dummy variables indicating carriers of the ApoE4 heterozygous and homozygous genotypes, respectively.

Table S2 (continued). Regression results (Casual blood glucose)

Casual blood glucose	Males						Females					
	Coef.	S.E.	t	P(t)	[95%	C.I.]	Coef.	S.E.	t	P(t)	[95%	C.I.]
1[Age<65] × Dietary 1	99.25	4.389	22.61	0.000	90.63	107.86	96.75	1.755	55.12	0.000	93.31	100.19
1[Age<65] × Dietary 2	105.94	4.655	22.76	0.000	96.80	115.07	95.20	1.885	50.50	0.000	91.50	98.90
1[Age<65] × Dietary 3	103.72	4.920	21.08	0.000	94.07	113.38	96.43	1.802	53.52	0.000	92.90	99.97
1[Age<65] × Dietary 4	100.50	5.925	16.96	0.000	88.87	112.13	100.80	2.011	50.13	0.000	96.86	104.75
1[Age<65] × Dietary 5	101.18	4.467	22.65	0.000	92.42	109.95	97.85	1.686	58.02	0.000	94.54	101.16
1[Age<65] × Dietary 6	98.69	6.745	14.63	0.000	85.45	111.93	98.67	2.101	46.97	0.000	94.55	102.79
1[Age≥65] × Dietary 1	101.72	4.211	24.15	0.000	93.45	109.98	97.36	2.052	47.44	0.000	93.34	101.39
1[Age≥65] × Dietary 2	105.38	4.208	25.05	0.000	97.12	113.64	98.00	2.012	48.70	0.000	94.06	101.95
1[Age≥65] × Dietary 3	101.15	4.294	23.56	0.000	92.73	109.58	99.48	1.873	53.11	0.000	95.80	103.15
1[Age≥65] × Dietary 4	102.53	4.399	23.31	0.000	93.90	111.16	99.92	1.799	55.55	0.000	96.39	103.44
1[Age≥65] × Dietary 5	101.13	4.180	24.19	0.000	92.93	109.34	100.05	1.711	58.49	0.000	96.69	103.40
1[Age≥65] × Dietary 6	103.07	5.022	20.53	0.000	93.22	112.93	99.58	1.921	51.84	0.000	95.82	103.35
School	-1.436	1.825	-0.787	0.432	-5.018	2.146	-2.046	0.789	-2.593	0.010	-3.594	-0.498
Non cohabitants	2.151	3.516	0.612	0.541	-4.750	9.051	-0.596	1.134	-0.526	0.599	-2.820	1.627
Lifestyle change	-1.897	1.550	-1.224	0.221	-4.940	1.146	-0.101	0.597	-0.169	0.866	-1.272	1.070
Paid	-3.220	1.843	-1.747	0.081	-6.836	0.397	-0.859	0.773	-1.111	0.267	-2.374	0.657
Well off	2.223	1.827	1.217	0.224	-1.364	5.809	0.027	0.716	0.038	0.970	-1.377	1.432
Alcohol	1.914	1.802	1.062	0.289	-1.624	5.451	0.319	0.712	0.447	0.655	-1.079	1.716
Smoking	1.760	2.583	0.682	0.496	-3.308	6.829	-0.788	1.684	-0.468	0.640	-4.091	2.514
Healthy 1	-4.985	3.541	-1.408	0.160	-11.94	1.965	-3.581	1.438	-2.490	0.013	-6.402	-0.760
Healthy 2	-3.813	2.621	-1.455	0.146	-8.958	1.331	-3.468	1.121	-3.093	0.002	-5.667	-1.268
Exercise 1	1.296	2.309	0.561	0.575	-3.236	5.828	-0.124	0.873	-0.142	0.887	-1.836	1.588
Exercise 2	-1.785	1.893	-0.943	0.346	-5.500	1.929	0.770	0.716	1.076	0.282	-0.634	2.174
Disease 1	3.246	1.851	1.754	0.080	-0.387	6.878	0.702	0.968	0.725	0.469	-1.198	2.602
Disease 2	20.70	2.660	7.784	0.000	15.48	25.92	5.591	2.138	2.615	0.009	1.396	9.785
Disease 3	-2.438	2.319	-1.051	0.293	-6.991	2.114	-0.980	0.970	-1.010	0.313	-2.882	0.923
Disease 4	-2.718	3.196	-0.850	0.395	-8.992	3.555	3.323	2.597	1.279	0.201	-1.772	8.417
Disease 5	1.093	2.649	0.413	0.680	-4.106	6.292	7.277	3.787	1.922	0.055	-0.151	14.71
disease 6	-0.851	1.619	-0.526	0.599	-4.029	2.327	-1.194	0.691	-1.729	0.084	-2.548	0.161
ApoE4 hetero	-0.550	1.958	-0.281	0.779	-4.392	3.292	-0.248	0.971	-0.255	0.799	-2.152	1.657
ApoE4 homo	17.14	10.41	1.646	0.100	-3.294	37.58	1.210	3.205	0.378	0.706	-5.077	7.498
Y2024	-2.810	1.139	-2.466	0.014	-5.046	-0.574	-3.016	0.420	-7.175	0.000	-3.840	-2.191
	N		915	F		3.802	N		1424	F		7.964
	R2 (Between)		0.217	P(F)		0.000	R2 (Between)		0.112	P(F)		0.000
	R2 (Within)		-0.016	σ2(u)		161.56	R2 (Within)		0.046	σ2(u)		73.98
	R2 (Overall)		0.153	σ2(ε)		272.46	R2 (Overall)		0.099	σ2(ε)		55.94
	Log-likelihood		-3869.5	σ2(u)/σ2(u+ε)		0.372	Log-likelihood		-4900.2	σ2(u)/σ2(u+ε)		0.569

Note: “Dietary” denotes the dietary habit cluster: “Dietary 1” is a dummy variable indicating whether an individual belongs to dietary habit cluster 1, and so on. “School”: an individual has 13 or more years of education; “Non cohabitants”: the individual lives alone; “Lifestyle change”: the individual experienced a major change in lifestyle during the past year; “Paid”: the individual is engaged in paid work; “Well off”: the household is financially comfortable; “Alcohol”: the individual has a drinking habit; “Smoking”: the individual has a smoking habit; “Health 1 (or 2)”: the subjective health status is “very” (or “fairly”) healthy; “Exercise1”: the individual engages in light exercise at least once per week; “Exercise2”: the individual engages in moderate or vigorous exercise at least once per week; “Y 2024”: that the survey year is 2024. “Disease1” through “Disease6” are indicators for diseases under treatment or with lasting effects: “Disease1” is hypertension, “Disease2” is diabetes, “Disease3” is hyperlipidemia, “Disease4” is heat disease, “Disease 5” is kindey disease,, and “Disease 6” is other diseases. “ApoE4 hetero” and “ApoE4 homo” are dummy variables indicating carriers of the ApoE4 heterozygous and homozygous genotypes, respectively.

Table S2 (continued). Regression results (HbA1c)

HbA1c	Males						Females					
	Coef.	S.E.	t	P(t)	[95%	C.I.]	Coef.	S.E.	t	P(t)	[95%	C.I.]
1[Age<65] × Dietary 1	5.621	0.096	58.75	0.000	5.433	5.809	5.698	0.058	98.59	0.000	5.584	5.811
1[Age<65] × Dietary 2	5.780	0.099	58.55	0.000	5.586	5.973	5.682	0.062	92.21	0.000	5.562	5.803
1[Age<65] × Dietary 3	5.655	0.102	55.38	0.000	5.455	5.855	5.718	0.058	97.98	0.000	5.603	5.832
1[Age<65] × Dietary 4	5.674	0.120	47.33	0.000	5.439	5.909	5.754	0.064	89.39	0.000	5.627	5.880
1[Age<65] × Dietary 5	5.712	0.095	60.40	0.000	5.526	5.897	5.731	0.055	103.68	0.000	5.622	5.839
1[Age<65] × Dietary 6	5.731	0.135	42.53	0.000	5.466	5.995	5.760	0.068	84.61	0.000	5.626	5.893
1[Age≥65] × Dietary 1	5.660	0.090	63.19	0.000	5.484	5.836	5.713	0.066	86.25	0.000	5.583	5.843
1[Age≥65] × Dietary 2	5.709	0.089	64.00	0.000	5.534	5.884	5.721	0.067	85.95	0.000	5.591	5.852
1[Age≥65] × Dietary 3	5.655	0.091	61.97	0.000	5.476	5.834	5.770	0.060	95.48	0.000	5.651	5.888
1[Age≥65] × Dietary 4	5.689	0.092	61.59	0.000	5.508	5.871	5.722	0.059	97.54	0.000	5.607	5.838
1[Age≥65] × Dietary 5	5.703	0.088	64.58	0.000	5.530	5.877	5.719	0.056	102.54	0.000	5.609	5.828
1[Age≥65] × Dietary 6	5.793	0.100	57.84	0.000	5.597	5.990	5.698	0.063	91.10	0.000	5.576	5.821
School	-0.012	0.053	-0.224	0.823	-0.115	0.091	-0.034	0.030	-1.119	0.263	-0.092	0.025
Non cohabitants	0.114	0.084	1.355	0.176	-0.051	0.278	-0.047	0.040	-1.170	0.242	-0.125	0.032
Lifestyle change	0.007	0.027	0.273	0.785	-0.045	0.059	-0.025	0.018	-1.384	0.167	-0.060	0.010
Paid	-0.019	0.041	-0.463	0.644	-0.099	0.061	-0.033	0.026	-1.261	0.208	-0.085	0.018
Well off	0.125	0.037	3.350	0.001	0.052	0.198	-0.017	0.023	-0.718	0.473	-0.061	0.029
Alcohol	-0.052	0.044	-1.195	0.232	-0.138	0.034	-0.040	0.024	-1.629	0.104	-0.087	0.008
Smoking	0.018	0.066	0.268	0.789	-0.112	0.148	0.021	0.060	0.349	0.727	-0.097	0.139
Healthy 1	-0.181	0.065	-2.804	0.005	-0.308	-0.054	-0.064	0.045	-1.407	0.160	-0.152	0.025
Healthy 2	-0.033	0.047	-0.701	0.484	-0.125	0.059	-0.080	0.035	-2.248	0.025	-0.149	-0.010
Exercise 1	0.048	0.041	1.156	0.248	-0.033	0.129	-0.005	0.027	-0.185	0.854	-0.058	0.048
Exercise 2	-0.037	0.035	-1.057	0.291	-0.106	0.032	0.017	0.023	0.763	0.445	-0.027	0.061
Disease 1	0.032	0.041	0.781	0.435	-0.048	0.112	0.037	0.032	1.147	0.252	-0.026	0.101
Disease 2	0.584	0.063	9.330	0.000	0.461	0.707	0.447	0.073	6.160	0.000	0.304	0.589
Disease 3	0.059	0.049	1.204	0.229	-0.037	0.156	0.019	0.032	0.595	0.552	-0.044	0.082
Disease 4	0.044	0.063	0.690	0.490	-0.081	0.168	0.163	0.088	1.863	0.063	-0.009	0.336
Disease 5	-0.026	0.049	-0.527	0.598	-0.123	0.071	0.000	0.116	0.004	0.997	-0.227	0.228
disease 6	-0.021	0.030	-0.690	0.490	-0.079	0.038	-0.017	0.022	-0.782	0.435	-0.060	0.026
ApoE4 hetero	-0.084	0.056	-1.485	0.138	-0.194	0.027	-0.008	0.037	-0.216	0.829	-0.080	0.065
ApoE4 homo	0.873	0.302	2.893	0.004	0.281	1.465	-0.072	0.122	-0.590	0.556	-0.310	0.167
Y2024	0.008	0.016	0.489	0.625	-0.023	0.039	-0.025	0.012	-2.157	0.031	-0.048	-0.002
	N		915	F		7.316	N		1424	F		13.857
	R2 (Between)		0.312	P(F)		0.000	R2 (Between)		0.185	P(F)		0.000
	R2 (Within)		-0.112	σ2(u)		0.197	R2 (Within)		-0.113	σ2(u)		0.116
	R2 (Overall)		0.286	σ2(ε)		0.043	R2 (Overall)		0.163	σ2(ε)		0.038
	Log-likelihood		65.6	σ2(u)/σ2(u+ε)		0.820	Log-likelihood		223.7	σ2(u)/σ2(u+ε)		0.753

Note: “Dietary” denotes the dietary habit cluster: “Dietary 1” is a dummy variable indicating whether an individual belongs to dietary habit cluster 1, and so on. “School”: an individual has 13 or more years of education; “Non cohabitants”: the individual lives alone; “Lifestyle change”: the individual experienced a major change in lifestyle during the past year; “Paid”: the individual is engaged in paid work; “Well off”: the household is financially comfortable; “Alcohol”: the individual has a drinking habit; “Smoking”: the individual has a smoking habit; “Health 1 (or 2)”: the subjective health status is “very” (or “fairly”) healthy; “Exercise1”: the individual engages in light exercise at least once per week; “Exercise2”: the individual engages in moderate or vigorous exercise at least once per week; “Y 2024”: that the survey year is 2024. “Disease1” through “Disease6” are indicators for diseases under treatment or with lasting effects: “Disease1” is hypertension, “Disease2” is diabetes, “Disease3” is hyperlipidemia, “Disease4” is heat disease, “Disease 5” is kindey disease,, and “Disease 6” is other diseases. “ApoE4 hetero” and “ApoE4 homo” are dummy variables indicating carriers of the ApoE4 heterozygous and homozygous genotypes, respectively.

Table S2 (continued). Regression results (BUN)

BUN	Males						Females					
	Coef.	S.E.	t	P(t)	[95%	C.I.]	Coef.	S.E.	t	P(t)	[95%	C.I.]
1[Age<65] × Dietary 1	14.62	0.739	19.79	0.000	13.17	16.07	14.38	0.528	27.25	0.000	13.34	15.41
1[Age<65] × Dietary 2	15.99	0.771	20.74	0.000	14.48	17.51	14.37	0.565	25.42	0.000	13.26	15.48
1[Age<65] × Dietary 3	13.83	0.808	17.11	0.000	12.24	15.42	14.90	0.539	27.67	0.000	13.84	15.96
1[Age<65] × Dietary 4	14.20	0.965	14.72	0.000	12.31	16.09	14.92	0.599	24.91	0.000	13.74	16.09
1[Age<65] × Dietary 5	14.79	0.740	20.00	0.000	13.34	16.25	14.79	0.506	29.23	0.000	13.80	15.78
1[Age<65] × Dietary 6	14.01	1.095	12.79	0.000	11.86	16.15	14.96	0.629	23.81	0.000	13.73	16.20
1[Age≥65] × Dietary 1	15.43	0.698	22.11	0.000	14.06	16.80	14.22	0.613	23.19	0.000	13.02	15.42
1[Age≥65] × Dietary 2	15.91	0.697	22.81	0.000	14.54	17.27	15.69	0.606	25.88	0.000	14.50	16.88
1[Age≥65] × Dietary 3	15.89	0.713	22.31	0.000	14.50	17.29	15.19	0.559	27.16	0.000	14.09	16.29
1[Age≥65] × Dietary 4	15.76	0.724	21.75	0.000	14.34	17.18	15.57	0.539	28.87	0.000	14.51	16.62
1[Age≥65] × Dietary 5	15.89	0.690	23.04	0.000	14.54	17.24	15.22	0.512	29.71	0.000	14.22	16.23
1[Age≥65] × Dietary 6	15.89	0.807	19.70	0.000	14.31	17.47	16.06	0.576	27.90	0.000	14.93	17.19
School	0.077	0.348	0.223	0.824	-0.605	0.759	-0.430	0.248	-1.735	0.083	-0.915	0.056
Non cohabitants	0.608	0.622	0.977	0.329	-0.613	1.829	0.080	0.348	0.228	0.819	-0.604	0.763
Lifestyle change	-0.114	0.233	-0.488	0.626	-0.572	0.344	-0.088	0.174	-0.504	0.614	-0.430	0.254
Paid	0.619	0.317	1.955	0.051	-0.003	1.241	0.013	0.235	0.054	0.957	-0.448	0.474
Well off	-0.430	0.302	-1.426	0.154	-1.022	0.162	0.062	0.214	0.292	0.771	-0.357	0.481
Alcohol	-0.994	0.322	-3.089	0.002	-1.625	-0.362	-0.381	0.217	-1.753	0.080	-0.806	0.045
Smoking	-0.626	0.472	-1.327	0.185	-1.551	0.300	-1.452	0.520	-2.793	0.005	-2.471	-0.432
Healthy 1	0.298	0.552	0.540	0.590	-0.785	1.381	0.355	0.427	0.833	0.405	-0.481	1.192
Healthy 2	0.205	0.405	0.507	0.612	-0.590	1.001	0.062	0.333	0.188	0.851	-0.591	0.716
Exercise 1	0.693	0.356	1.948	0.052	-0.005	1.391	0.287	0.257	1.115	0.265	-0.218	0.792
Exercise 2	0.388	0.297	1.304	0.193	-0.196	0.971	0.038	0.212	0.177	0.859	-0.378	0.454
Disease 1	0.089	0.317	0.280	0.779	-0.533	0.711	0.522	0.293	1.779	0.076	-0.054	1.097
Disease 2	0.398	0.470	0.847	0.397	-0.524	1.319	-0.236	0.650	-0.363	0.717	-1.510	1.039
Disease 3	-0.219	0.391	-0.560	0.575	-0.987	0.549	0.180	0.292	0.616	0.538	-0.393	0.754
Disease 4	0.157	0.521	0.301	0.764	-0.866	1.179	-0.668	0.789	-0.848	0.397	-2.215	0.879
Disease 5	0.461	0.419	1.101	0.271	-0.361	1.282	1.262	1.113	1.134	0.257	-0.920	3.445
disease 6	-0.285	0.253	-1.126	0.261	-0.783	0.212	0.190	0.205	0.927	0.354	-0.212	0.592
ApoE4 hetero	0.168	0.373	0.450	0.653	-0.563	0.899	-0.158	0.305	-0.518	0.604	-0.756	0.440
ApoE4 homo	3.513	1.989	1.766	0.078	-0.392	7.417	-0.389	1.006	-0.387	0.699	-2.362	1.584
Y2024	0.751	0.150	5.008	0.000	0.456	1.045	0.855	0.119	7.195	0.000	0.622	1.089
	N		915	F		3.300	N		1424	F		4.574
	R2 (Between)		0.104	P(F)		0.000	R2 (Between)		0.085	P(F)		0.000
	R2 (Within)		0.091	σ2(u)		9.175	R2 (Within)		0.077	σ2(u)		8.183
	R2 (Overall)		0.103	σ2(ε)		4.889	R2 (Overall)		0.083	σ2(ε)		4.617
	Log-likelihood		-2003.1	σ2(u)/σ2(u+ε)		0.652	Log-likelihood		-3096.3	σ2(u)/σ2(u+ε)		0.639

Note: "Dietary" denotes the dietary habit cluster: "Dietary 1" is a dummy variable indicating whether an individual belongs to dietary habit cluster 1, and so on. "School": an individual has 13 or more years of education; "Non cohabitants": the individual lives alone; "Lifestyle change": the individual experienced a major change in lifestyle during the past year; "Paid": the individual is engaged in paid work; "Well off": the household is financially comfortable; "Alcohol": the individual has a drinking habit; "Smoking": the individual has a smoking habit; "Health 1 (or 2)": the subjective health status is "very" (or "fairly") healthy; "Exercise1": the individual engages in light exercise at least once per week; "Exercise2": the individual engages in moderate or vigorous exercise at least once per week; "Y 2024": that the survey year is 2024. "Disease1" through "Disease6" are indicators for diseases under treatment or with lasting effects: "Disease1" is hypertension, "Disease2" is diabetes, "Disease3" is hyperlipidemia, "Disease4" is heat disease, "Disease 5" is kindey disease,, and "Disease 6" is other diseases. "ApoE4 hetero" and "ApoE4 homo" are dummy variables indicating carriers of the ApoE4 heterozygous and homozygous genotypes, respectively.

Table S2 (continued). Regression results (Creatinine)

Creatinine	Males						Females					
	Coef.	S.E.	t	P(t)	[95%	C.I.]	Coef.	S.E.	t	P(t)	[95%	C.I.]
1[Age<65] × Dietary 1	0.959	0.025	38.24	0.000	0.910	1.008	0.700	0.013	54.62	0.000	0.675	0.726
1[Age<65] × Dietary 2	0.962	0.026	37.42	0.000	0.911	1.012	0.690	0.014	50.83	0.000	0.663	0.716
1[Age<65] × Dietary 3	0.946	0.026	35.97	0.000	0.894	0.997	0.702	0.013	54.93	0.000	0.677	0.727
1[Age<65] × Dietary 4	0.973	0.030	32.09	0.000	0.914	1.033	0.689	0.014	49.50	0.000	0.662	0.716
1[Age<65] × Dietary 5	0.960	0.025	38.98	0.000	0.912	1.008	0.700	0.012	57.15	0.000	0.676	0.724
1[Age<65] × Dietary 6	0.939	0.034	27.85	0.000	0.872	1.005	0.710	0.015	47.92	0.000	0.681	0.739
1[Age≥65] × Dietary 1	0.976	0.024	41.59	0.000	0.930	1.022	0.704	0.014	48.96	0.000	0.676	0.733
1[Age≥65] × Dietary 2	1.003	0.023	43.05	0.000	0.957	1.049	0.681	0.015	46.18	0.000	0.652	0.710
1[Age≥65] × Dietary 3	0.965	0.024	40.47	0.000	0.918	1.011	0.692	0.013	52.51	0.000	0.666	0.718
1[Age≥65] × Dietary 4	0.983	0.024	40.82	0.000	0.936	1.030	0.694	0.013	53.86	0.000	0.669	0.720
1[Age≥65] × Dietary 5	0.984	0.023	42.52	0.000	0.938	1.029	0.697	0.012	56.68	0.000	0.673	0.721
1[Age≥65] × Dietary 6	0.993	0.026	38.75	0.000	0.942	1.043	0.710	0.014	51.87	0.000	0.683	0.737
School	0.006	0.016	0.375	0.708	-0.025	0.037	-0.004	0.008	-0.487	0.627	-0.019	0.012
Non cohabitants	0.018	0.022	0.819	0.413	-0.025	0.062	-0.001	0.009	-0.117	0.907	-0.019	0.017
Lifestyle change	-0.004	0.006	-0.621	0.535	-0.016	0.008	-0.002	0.004	-0.504	0.615	-0.009	0.005
Paid	0.000	0.010	0.039	0.969	-0.020	0.021	-0.009	0.006	-1.530	0.126	-0.021	0.003
Well off	-0.025	0.009	-2.707	0.007	-0.043	-0.007	0.002	0.005	0.320	0.749	-0.008	0.011
Alcohol	-0.028	0.012	-2.380	0.018	-0.050	-0.005	-0.008	0.006	-1.476	0.140	-0.019	0.003
Smoking	-0.015	0.018	-0.822	0.411	-0.051	0.021	0.012	0.014	0.813	0.417	-0.016	0.040
Healthy 1	-0.007	0.015	-0.468	0.640	-0.038	0.023	-0.004	0.009	-0.400	0.690	-0.022	0.015
Healthy 2	-0.001	0.011	-0.053	0.958	-0.023	0.021	0.009	0.007	1.174	0.241	-0.006	0.023
Exercise 1	-0.021	0.010	-2.122	0.034	-0.040	-0.002	0.003	0.006	0.505	0.614	-0.008	0.014
Exercise 2	0.001	0.008	0.084	0.933	-0.016	0.017	0.008	0.005	1.736	0.083	-0.001	0.017
Disease 1	0.013	0.010	1.288	0.198	-0.007	0.034	0.014	0.007	1.887	0.059	-0.001	0.028
Disease 2	-0.030	0.016	-1.834	0.067	-0.062	0.002	-0.014	0.016	-0.871	0.384	-0.046	0.018
Disease 3	0.010	0.012	0.826	0.409	-0.014	0.034	0.009	0.007	1.249	0.212	-0.005	0.022
Disease 4	0.000	0.016	0.009	0.993	-0.030	0.031	-0.020	0.020	-1.023	0.307	-0.058	0.018
Disease 5	0.009	0.012	0.791	0.429	-0.014	0.033	0.041	0.024	1.723	0.085	-0.006	0.087
disease 6	-0.011	0.007	-1.557	0.120	-0.025	0.003	-0.003	0.005	-0.669	0.504	-0.012	0.006
ApoE4 hetero	0.005	0.017	0.280	0.780	-0.028	0.038	-0.002	0.010	-0.209	0.835	-0.021	0.017
ApoE4 homo	0.061	0.091	0.668	0.505	-0.118	0.239	-0.053	0.032	-1.671	0.095	-0.115	0.009
Y2024	0.029	0.004	7.932	0.000	0.021	0.036	0.020	0.002	8.842	0.000	0.015	0.024
	N		915	F		3.811	N		1424	F		7.463
	R2 (Between)		0.048	P(F)		0.000	R2 (Between)		0.030	P(F)		0.000
	R2 (Within)		0.167	σ2(u)		0.023	R2 (Within)		0.152	σ2(u)		0.010
	R2 (Overall)		0.054	σ2(ε)		0.003	R2 (Overall)		0.038	σ2(ε)		0.002
	Log-likelihood		1429.4	σ2(u)/σ2(u+ε)		0.896	Log-likelihood		2584.1	σ2(u)/σ2(u+ε)		0.861

Note: "Dietary" denotes the dietary habit cluster: "Dietary 1" is a dummy variable indicating whether an individual belongs to dietary habit cluster 1, and so on. "School": an individual has 13 or more years of education; "Non cohabitants": the individual lives alone; "Lifestyle change": the individual experienced a major change in lifestyle during the past year; "Paid": the individual is engaged in paid work; "Well off": the household is financially comfortable; "Alcohol": the individual has a drinking habit; "Smoking": the individual has a smoking habit; "Health 1 (or 2)": the subjective health status is "very" (or "fairly") healthy; "Exercise1": the individual engages in light exercise at least once per week; "Exercise2": the individual engages in moderate or vigorous exercise at least once per week; "Y 2024": that the survey year is 2024. "Disease1" through "Disease6" are indicators for diseases under treatment or with lasting effects: "Disease1" is hypertension, "Disease2" is diabetes, "Disease3" is hyperlipidemia, "Disease4" is heat disease, "Disease 5" is kindey disease,, and "Disease 6" is other diseases. "ApoE4 hetero" and "ApoE4 homo" are dummy variables indicating carriers of the ApoE4 heterozygous and homozygous genotypes, respectively.

Table S2 (continued). Regression results (UA)

Uric acid	Males						Females					
	Coef.	S.E.	t	P(t)	[95%	C.I.]	Coef.	S.E.	t	P(t)	[95%	C.I.]
1[Age<65] × Dietary 1	5.760	0.205	28.13	0.000	5.358	6.162	4.752	0.127	37.39	0.000	4.503	5.001
1[Age<65] × Dietary 2	5.718	0.212	26.98	0.000	5.303	6.134	4.660	0.135	34.57	0.000	4.395	4.924
1[Age<65] × Dietary 3	5.610	0.220	25.48	0.000	5.178	6.042	4.709	0.127	37.04	0.000	4.460	4.959
1[Age<65] × Dietary 4	5.519	0.260	21.23	0.000	5.009	6.029	4.698	0.139	33.80	0.000	4.425	4.970
1[Age<65] × Dietary 5	5.778	0.203	28.45	0.000	5.380	6.177	4.760	0.121	39.22	0.000	4.522	4.998
1[Age<65] × Dietary 6	5.484	0.293	18.70	0.000	4.908	6.059	4.762	0.148	32.24	0.000	4.473	5.052
1[Age≥65] × Dietary 1	5.645	0.192	29.39	0.000	5.268	6.022	4.695	0.144	32.71	0.000	4.414	4.977
1[Age≥65] × Dietary 2	5.866	0.192	30.63	0.000	5.490	6.242	4.647	0.146	31.76	0.000	4.360	4.934
1[Age≥65] × Dietary 3	5.773	0.196	29.47	0.000	5.389	6.158	4.751	0.131	36.20	0.000	4.494	5.009
1[Age≥65] × Dietary 4	5.796	0.199	29.20	0.000	5.406	6.186	4.807	0.128	37.51	0.000	4.555	5.058
1[Age≥65] × Dietary 5	5.673	0.190	29.94	0.000	5.301	6.045	4.747	0.122	38.89	0.000	4.507	4.986
1[Age≥65] × Dietary 6	5.692	0.217	26.24	0.000	5.266	6.118	4.821	0.136	35.38	0.000	4.554	5.089
School	0.041	0.107	0.381	0.703	-0.169	0.251	-0.086	0.073	-1.175	0.240	-0.230	0.058
Non cohabitants	0.253	0.178	1.421	0.156	-0.096	0.601	-0.138	0.091	-1.527	0.127	-0.316	0.039
Lifestyle change	0.048	0.059	0.803	0.422	-0.069	0.164	-0.025	0.037	-0.676	0.499	-0.098	0.048
Paid	0.011	0.088	0.123	0.902	-0.162	0.183	-0.085	0.058	-1.466	0.143	-0.199	0.029
Well off	0.012	0.081	0.149	0.881	-0.147	0.171	-0.015	0.049	-0.306	0.760	-0.111	0.081
Alcohol	0.055	0.092	0.598	0.550	-0.126	0.237	0.042	0.054	0.771	0.441	-0.065	0.148
Smoking	0.045	0.139	0.324	0.746	-0.227	0.317	0.184	0.139	1.322	0.186	-0.089	0.456
Healthy 1	-0.124	0.143	-0.870	0.385	-0.405	0.156	-0.080	0.096	-0.838	0.403	-0.267	0.107
Healthy 2	-0.044	0.104	-0.426	0.670	-0.249	0.160	-0.089	0.075	-1.192	0.234	-0.236	0.058
Exercise 1	-0.200	0.092	-2.180	0.030	-0.379	-0.020	-0.101	0.057	-1.787	0.074	-0.212	0.010
Exercise 2	0.014	0.077	0.184	0.854	-0.138	0.166	0.015	0.047	0.319	0.750	-0.078	0.108
Disease 1	0.180	0.088	2.047	0.041	0.007	0.352	0.244	0.071	3.432	0.001	0.105	0.384
Disease 2	-0.470	0.133	-3.530	0.000	-0.732	-0.209	-0.184	0.161	-1.145	0.253	-0.500	0.132
Disease 3	-0.066	0.107	-0.615	0.539	-0.275	0.144	0.021	0.070	0.301	0.763	-0.116	0.157
Disease 4	-0.180	0.139	-1.297	0.195	-0.452	0.092	0.236	0.194	1.218	0.224	-0.144	0.616
Disease 5	-0.110	0.109	-1.004	0.316	-0.324	0.105	0.425	0.241	1.766	0.078	-0.047	0.897
disease 6	-0.149	0.066	-2.261	0.024	-0.278	-0.020	-0.027	0.046	-0.591	0.555	-0.117	0.063
ApoE4 hetero	0.077	0.115	0.672	0.502	-0.148	0.302	-0.025	0.091	-0.274	0.784	-0.203	0.153
ApoE4 homo	0.084	0.614	0.138	0.891	-1.121	1.290	-0.547	0.298	-1.835	0.067	-1.133	0.038
Y2024	-0.026	0.036	-0.723	0.470	-0.096	0.045	0.006	0.023	0.267	0.790	-0.039	0.052
	N		915	F		1.875	N		1424	F		2.660
	R2 (Between)		0.088	P(F)		0.003	R2 (Between)		0.041	P(F)		0.000
	R2 (Within)		0.003	σ2(u)		0.951	R2 (Within)		0.017	σ2(u)		0.836
	R2 (Overall)		0.080	σ2(ε)		0.269	R2 (Overall)		0.039	σ2(ε)		0.167
	Log-likelihood		-687.5	σ2(u)/σ2(u+ε)		0.780	Log-likelihood		-746.5	σ2(u)/σ2(u+ε)		0.834

Note: “Dietary” denotes the dietary habit cluster: “Dietary 1” is a dummy variable indicating whether an individual belongs to dietary habit cluster 1, and so on. “School”: an individual has 13 or more years of education; “Non cohabitants”: the individual lives alone; “Lifestyle change”: the individual experienced a major change in lifestyle during the past year; “Paid”: the individual is engaged in paid work; “Well off”: the household is financially comfortable; “Alcohol”: the individual has a drinking habit; “Smoking”: the individual has a smoking habit; “Health 1 (or 2)”: the subjective health status is “very” (or “fairly”) healthy; “Exercise1”: the individual engages in light exercise at least once per week; “Exercise2”: the individual engages in moderate or vigorous exercise at least once per week; “Y 2024”: that the survey year is 2024. “Disease1” through “Disease6” are indicators for diseases under treatment or with lasting effects: “Disease1” is hypertension, “Disease2” is diabetes, “Disease3” is hyperlipidemia, “Disease4” is heat disease, “Disease 5” is kindey disease,, and “Disease 6” is other diseases. “ApoE4 hetero” and “ApoE4 homo” are dummy variables indicating carriers of the ApoE4 heterozygous and homozygous genotypes, respectively.

Table S2 (continued). Regression results (Triglycerides)

Triglycerides	Males						Females					
	Coef.	S.E.	t	P(t)	[95%	C.I.]	Coef.	S.E.	t	P(t)	[95%	C.I.]
1[Age<65] × Dietary 1	167.21	17.62	9.488	0.000	132.62	201.79	122.62	11.24	10.91	0.000	100.57	144.67
1[Age<65] × Dietary 2	140.75	18.45	7.629	0.000	104.54	176.96	125.53	12.09	10.39	0.000	101.82	149.24
1[Age<65] × Dietary 3	162.11	19.38	8.366	0.000	124.08	200.14	138.92	11.57	12.01	0.000	116.22	161.61
1[Age<65] × Dietary 4	142.54	23.18	6.150	0.000	97.05	188.03	128.69	12.93	9.949	0.000	103.32	154.06
1[Age<65] × Dietary 5	143.52	17.71	8.106	0.000	108.77	178.26	134.19	10.81	12.41	0.000	112.98	155.39
1[Age<65] × Dietary 6	134.29	26.33	5.100	0.000	82.61	185.96	137.29	13.48	10.18	0.000	110.84	163.74
1[Age≥65] × Dietary 1	164.71	16.70	9.865	0.000	131.94	197.48	133.81	13.18	10.15	0.000	107.96	159.67
1[Age≥65] × Dietary 2	149.74	16.69	8.973	0.000	116.99	182.49	136.25	12.87	10.59	0.000	111.00	161.50
1[Age≥65] × Dietary 3	156.93	17.04	9.207	0.000	123.48	190.38	138.05	12.04	11.47	0.000	114.44	161.66
1[Age≥65] × Dietary 4	151.74	17.35	8.746	0.000	117.69	185.79	138.04	11.53	11.97	0.000	115.42	160.67
1[Age≥65] × Dietary 5	155.71	16.51	9.432	0.000	123.31	188.12	132.98	10.97	12.12	0.000	111.45	154.50
1[Age≥65] × Dietary 6	159.95	19.42	8.237	0.000	121.84	198.06	132.40	12.32	10.75	0.000	108.23	156.57
School	-1.539	8.075	-0.191	0.849	-17.39	14.31	-7.300	4.955	-1.473	0.141	-17.02	2.419
Non cohabitants	-7.493	14.70	-0.510	0.611	-36.35	21.37	-11.89	7.183	-1.655	0.098	-25.98	2.202
Lifestyle change	-2.810	5.698	-0.493	0.622	-13.99	8.373	2.024	3.876	0.522	0.602	-5.580	9.628
Paid	-15.53	7.540	-2.059	0.040	-30.33	-0.729	-2.854	4.919	-0.580	0.562	-12.50	6.795
Well off	-6.185	7.239	-0.855	0.393	-20.39	8.022	3.701	4.598	0.805	0.421	-5.318	12.72
Alcohol	2.948	7.592	0.388	0.698	-11.95	17.85	-2.770	4.530	-0.611	0.541	-11.66	6.117
Smoking	16.05	11.07	1.450	0.148	-5.679	37.77	15.62	10.65	1.467	0.143	-5.271	36.50
Healthy 1	-19.08	13.39	-1.425	0.155	-45.35	7.200	-16.95	9.265	-1.830	0.068	-35.12	1.224
Healthy 2	-6.645	9.845	-0.675	0.500	-25.97	12.68	-13.08	7.218	-1.812	0.070	-27.24	1.079
Exercise 1	2.614	8.646	0.302	0.762	-14.36	19.58	-9.035	5.634	-1.604	0.109	-20.09	2.018
Exercise 2	-3.744	7.200	-0.520	0.603	-17.87	10.39	6.043	4.614	1.310	0.191	-3.007	15.09
Disease 1	15.68	7.550	2.077	0.038	0.864	30.50	11.28	6.175	1.827	0.068	-0.834	23.39
Disease 2	-14.79	11.11	-1.332	0.183	-36.59	7.010	37.49	13.62	2.753	0.006	10.78	64.20
Disease 3	-1.853	9.348	-0.198	0.843	-20.20	16.49	11.48	6.199	1.851	0.064	-0.686	23.64
Disease 4	-8.856	12.53	-0.707	0.480	-33.45	15.74	24.66	16.54	1.490	0.136	-7.796	57.11
Disease 5	-13.94	10.13	-1.376	0.169	-33.83	5.947	11.55	24.50	0.472	0.637	-36.51	59.62
disease 6	-10.89	6.144	-1.773	0.077	-22.95	1.165	-7.183	4.447	-1.615	0.107	-15.91	1.540
ApoE4 hetero	12.54	8.657	1.448	0.148	-4.454	29.53	6.436	6.094	1.056	0.291	-5.519	18.39
ApoE4 homo	-46.73	46.20	-1.012	0.312	-137.41	43.95	18.71	20.13	0.930	0.353	-20.78	58.20
Y2024	10.50	3.735	2.810	0.005	3.164	17.83	5.896	2.776	2.124	0.034	0.451	11.34
	N		915	F		1.423	N		1424	F		2.001
	R2 (Between)		0.059	P(F)		0.064	R2 (Between)		0.083	P(F)		0.001
	R2 (Within)		0.038	σ2(u)		4609.97	R2 (Within)		-0.001	σ2(u)		2874.08
	R2 (Overall)		0.055	σ2(ε)		2972.39	R2 (Overall)		0.065	σ2(ε)		2524.93
	Log-likelihood		-4947.7	σ2(u)/σ2(u+ε)		0.608	Log-likelihood		-7590.8	σ2(u)/σ2(u+ε)		0.532

Note: "Dietary" denotes the dietary habit cluster: "Dietary 1" is a dummy variable indicating whether an individual belongs to dietary habit cluster 1, and so on. "School": an individual has 13 or more years of education; "Non cohabitants": the individual lives alone; "Lifestyle change": the individual experienced a major change in lifestyle during the past year; "Paid": the individual is engaged in paid work; "Well off": the household is financially comfortable; "Alcohol": the individual has a drinking habit; "Smoking": the individual has a smoking habit; "Health 1 (or 2)": the subjective health status is "very" (or "fairly") healthy; "Exercise1": the individual engages in light exercise at least once per week; "Exercise2": the individual engages in moderate or vigorous exercise at least once per week; "Y 2024": that the survey year is 2024. "Disease1" through "Disease6" are indicators for diseases under treatment or with lasting effects: "Disease1" is hypertension, "Disease2" is diabetes, "Disease3" is hyperlipidemia, "Disease4" is heat disease, "Disease 5" is kindey disease,, and "Disease 6" is other diseases. "ApoE4 hetero" and "ApoE4 homo" are dummy variables indicating carriers of the ApoE4 heterozygous and homozygous genotypes, respectively.

Table S2 (continued). Regression results (Total cholesterol)

Total cholesterol	Males						Females					
	Coef.	S.E.	t	P(t)	[95%	C.I.]	Coef.	S.E.	t	P(t)	[95%	C.I.]
1[Age<65] × Dietary 1	214.13	5.929	36.12	0.000	202.49	225.77	236.13	4.575	51.61	0.000	227.16	245.11
1[Age<65] × Dietary 2	213.19	6.144	34.70	0.000	201.14	225.25	232.18	4.867	47.71	0.000	222.63	241.73
1[Age<65] × Dietary 3	212.53	6.393	33.25	0.000	199.98	225.08	234.72	4.599	51.04	0.000	225.70	243.74
1[Age<65] × Dietary 4	218.26	7.562	28.86	0.000	203.41	233.10	239.25	5.052	47.36	0.000	229.34	249.16
1[Age<65] × Dietary 5	209.73	5.889	35.61	0.000	198.17	221.29	239.09	4.372	54.69	0.000	230.52	247.67
1[Age<65] × Dietary 6	218.51	8.539	25.59	0.000	201.75	235.26	242.44	5.357	45.26	0.000	231.93	252.95
1[Age≥65] × Dietary 1	206.76	5.567	37.14	0.000	195.83	217.68	229.87	5.208	44.14	0.000	219.65	240.09
1[Age≥65] × Dietary 2	209.89	5.553	37.80	0.000	198.99	220.79	240.90	5.271	45.71	0.000	230.56	251.24
1[Age≥65] × Dietary 3	212.29	5.678	37.39	0.000	201.15	223.43	234.48	4.755	49.32	0.000	225.16	243.81
1[Age≥65] × Dietary 4	213.78	5.756	37.14	0.000	202.49	225.08	238.26	4.630	51.46	0.000	229.18	247.34
1[Age≥65] × Dietary 5	213.07	5.491	38.80	0.000	202.29	223.84	233.18	4.404	52.94	0.000	224.54	241.82
1[Age≥65] × Dietary 6	211.37	6.307	33.51	0.000	198.99	223.75	232.01	4.931	47.05	0.000	222.34	241.69
School	-1.904	3.047	-0.625	0.532	-7.885	4.077	-1.533	2.492	-0.615	0.539	-6.421	3.355
Non cohabitants	0.803	5.127	0.157	0.876	-9.259	10.87	-4.727	3.207	-1.474	0.141	-11.02	1.564
Lifestyle change	2.629	1.739	1.512	0.131	-0.784	6.041	0.236	1.378	0.171	0.864	-2.468	2.939
Paid	-1.058	2.548	-0.415	0.678	-6.060	3.943	0.347	2.087	0.166	0.868	-3.748	4.441
Well off	1.058	2.362	0.448	0.654	-3.577	5.693	-0.268	1.790	-0.150	0.881	-3.779	3.244
Alcohol	6.996	2.663	2.627	0.009	1.770	12.22	2.303	1.944	1.185	0.236	-1.510	6.116
Smoking	2.957	3.978	0.743	0.457	-4.850	10.77	3.151	4.875	0.646	0.518	-6.413	12.71
Healthy 1	-1.494	4.184	-0.357	0.721	-9.705	6.717	-0.917	3.514	-0.261	0.794	-7.811	5.977
Healthy 2	-2.705	3.057	-0.885	0.377	-8.705	3.295	2.829	2.754	1.027	0.305	-2.574	8.231
Exercise 1	-1.201	2.682	-0.448	0.655	-6.465	4.064	-0.917	2.092	-0.438	0.661	-5.021	3.187
Exercise 2	1.485	2.264	0.656	0.512	-2.959	5.929	-0.571	1.746	-0.327	0.744	-3.996	2.854
Disease 1	-3.592	2.543	-1.412	0.158	-8.584	1.400	-0.297	2.570	-0.116	0.908	-5.339	4.745
Disease 2	-9.552	3.848	-2.482	0.013	-17.11	-1.999	-17.49	5.773	-3.030	0.003	-28.82	-6.166
Disease 3	-5.799	3.101	-1.870	0.062	-11.89	0.287	-20.18	2.523	-7.995	0.000	-25.13	-15.23
Disease 4	-9.899	4.046	-2.447	0.015	-17.84	-1.959	-7.320	6.968	-1.051	0.294	-20.99	6.349
Disease 5	-5.957	3.190	-1.867	0.062	-12.22	0.305	2.225	8.929	0.249	0.803	-15.29	19.74
disease 6	-0.493	1.923	-0.256	0.798	-4.268	3.282	0.386	1.690	0.229	0.819	-2.929	3.702
ApoE4 hetero	7.519	3.266	2.302	0.022	1.110	13.93	5.240	3.077	1.703	0.089	-0.797	11.28
ApoE4 homo	-14.14	17.48	-0.809	0.419	-48.45	20.17	3.723	10.13	0.368	0.713	-16.15	23.59
Y2024	0.218	1.061	0.206	0.837	-1.864	2.300	2.183	0.878	2.487	0.013	0.461	3.906
	N		915	F		3.069	N		1424	F		7.366
	R2 (Between)		0.110	P(F)		0.000	R2 (Between)		0.119	P(F)		0.000
	R2 (Within)		0.014	σ2(u)		771.65	R2 (Within)		0.048	σ2(u)		944.10
	R2 (Overall)		0.100	σ2(ε)		238.84	R2 (Overall)		0.111	σ2(ε)		243.66
	Log-likelihood		-3787.4	σ2(u)/σ2(u+ε)		0.764	Log-likelihood		-5927.3	σ2(u)/σ2(u+ε)		0.795

Note: "Dietary" denotes the dietary habit cluster: "Dietary 1" is a dummy variable indicating whether an individual belongs to dietary habit cluster 1, and so on. "School": an individual has 13 or more years of education; "Non cohabitants": the individual lives alone; "Lifestyle change": the individual experienced a major change in lifestyle during the past year; "Paid": the individual is engaged in paid work; "Well off": the household is financially comfortable; "Alcohol": the individual has a drinking habit; "Smoking": the individual has a smoking habit; "Health 1 (or 2)": the subjective health status is "very" (or "fairly") healthy; "Exercise1": the individual engages in light exercise at least once per week; "Exercise2": the individual engages in moderate or vigorous exercise at least once per week; "Y 2024": that the survey year is 2024. "Disease1" through "Disease6" are indicators for diseases under treatment or with lasting effects: "Disease1" is hypertension, "Disease2" is diabetes, "Disease3" is hyperlipidemia, "Disease4" is heat disease, "Disease 5" is kindey disease,, and "Disease 6" is other diseases. "ApoE4 hetero" and "ApoE4 homo" are dummy variables indicating carriers of the ApoE4 heterozygous and homozygous genotypes, respectively.

Table S2 (continued). Regression results (HDL-C)

HDL-C	Males						Females					
	Coef.	S.E.	t	P(t)	[95%	C.I.]	Coef.	S.E.	t	P(t)	[95%	C.I.]
1[Age<65] × Dietary 1	59.35	2.794	21.25	0.000	53.87	64.84	75.56	1.991	37.95	0.000	71.65	79.46
1[Age<65] × Dietary 2	60.92	2.862	21.28	0.000	55.30	66.53	74.49	2.099	35.48	0.000	70.37	78.61
1[Age<65] × Dietary 3	61.84	2.926	21.14	0.000	56.10	67.58	74.85	1.977	37.86	0.000	70.97	78.73
1[Age<65] × Dietary 4	64.46	3.370	19.13	0.000	57.84	71.07	76.87	2.142	35.89	0.000	72.67	81.07
1[Age<65] × Dietary 5	60.96	2.742	22.23	0.000	55.58	66.34	75.29	1.902	39.59	0.000	71.56	79.02
1[Age<65] × Dietary 6	62.19	3.742	16.62	0.000	54.84	69.53	74.93	2.280	32.86	0.000	70.46	79.40
1[Age≥65] × Dietary 1	60.31	2.613	23.08	0.000	55.18	65.44	73.62	2.216	33.22	0.000	69.27	77.97
1[Age≥65] × Dietary 2	63.25	2.595	24.37	0.000	58.15	68.34	74.13	2.283	32.47	0.000	69.65	78.61
1[Age≥65] × Dietary 3	61.29	2.655	23.08	0.000	56.08	66.50	72.38	2.034	35.58	0.000	68.39	76.37
1[Age≥65] × Dietary 4	62.86	2.682	23.44	0.000	57.59	68.12	73.12	1.996	36.64	0.000	69.20	77.03
1[Age≥65] × Dietary 5	64.26	2.578	24.93	0.000	59.20	69.31	73.67	1.908	38.61	0.000	69.93	77.42
1[Age≥65] × Dietary 6	61.15	2.850	21.46	0.000	55.56	66.75	74.23	2.115	35.10	0.000	70.08	78.38
School	-0.678	1.773	-0.382	0.702	-4.158	2.802	-0.329	1.290	-0.255	0.799	-2.859	2.202
Non cohabitants	5.595	2.461	2.274	0.023	0.766	10.43	-0.064	1.448	-0.044	0.965	-2.905	2.777
Lifestyle change	0.256	0.691	0.371	0.711	-1.099	1.612	0.172	0.541	0.318	0.750	-0.889	1.234
Paid	1.885	1.152	1.636	0.102	-0.376	4.146	2.709	0.906	2.991	0.003	0.932	4.486
Well off	1.276	1.014	1.259	0.208	-0.713	3.266	-0.285	0.734	-0.388	0.698	-1.725	1.156
Alcohol	5.816	1.286	4.523	0.000	3.292	8.340	3.519	0.850	4.142	0.000	1.853	5.186
Smoking	-2.957	2.023	-1.462	0.144	-6.928	1.013	-0.927	2.255	-0.411	0.681	-5.350	3.496
Healthy 1	0.479	1.706	0.281	0.779	-2.870	3.828	0.756	1.425	0.531	0.596	-2.039	3.550
Healthy 2	-2.542	1.237	-2.055	0.040	-4.969	-0.114	1.706	1.118	1.525	0.127	-0.488	3.900
Exercise 1	1.061	1.086	0.977	0.329	-1.071	3.193	0.590	0.839	0.704	0.482	-1.055	2.235
Exercise 2	1.296	0.931	1.392	0.164	-0.531	3.122	-0.021	0.708	-0.029	0.977	-1.410	1.369
Disease 1	-1.996	1.144	-1.744	0.082	-4.241	0.250	-1.254	1.098	-1.142	0.254	-3.408	0.899
Disease 2	-1.433	1.813	-0.790	0.430	-4.990	2.125	-5.819	2.508	-2.320	0.021	-10.74	-0.899
Disease 3	0.433	1.360	0.318	0.750	-2.236	3.101	-1.356	1.063	-1.275	0.203	-3.442	0.730
Disease 4	2.017	1.714	1.177	0.240	-1.347	5.381	-6.053	3.003	-2.016	0.044	-11.94	-0.163
Disease 5	-0.362	1.306	-0.277	0.782	-2.926	2.202	-3.159	3.541	-0.892	0.372	-10.11	3.786
disease 6	0.343	0.784	0.438	0.662	-1.196	1.882	-0.208	0.685	-0.304	0.761	-1.552	1.135
ApoE4 hetero	-0.418	1.899	-0.220	0.826	-4.146	3.309	-2.443	1.598	-1.529	0.127	-5.577	0.692
ApoE4 homo	7.129	10.20	0.699	0.485	-12.90	27.15	-3.007	5.253	-0.572	0.567	-13.31	7.297
Y2024	-0.044	0.397	-0.110	0.912	-0.822	0.735	0.227	0.332	0.685	0.494	-0.424	0.878
	N		915	F		2.852	N		1424	F		3.466
	R2 (Between)		0.107	P(F)		0.000	R2 (Between)		0.075	P(F)		0.000
	R2 (Within)		0.038	σ2(u)		280.42	R2 (Within)		0.013	σ2(u)		272.80
	R2 (Overall)		0.101	σ2(ε)		31.41	R2 (Overall)		0.072	σ2(ε)		34.09
	Log-likelihood		-2874.4	σ2(u)/σ2(u+ε)		0.899	Log-likelihood		-4525.6	σ2(u)/σ2(u+ε)		0.889

Note: "Dietary" denotes the dietary habit cluster: "Dietary 1" is a dummy variable indicating whether an individual belongs to dietary habit cluster 1, and so on. "School": an individual has 13 or more years of education; "Non cohabitants": the individual lives alone; "Lifestyle change": the individual experienced a major change in lifestyle during the past year; "Paid": the individual is engaged in paid work; "Well off": the household is financially comfortable; "Alcohol": the individual has a drinking habit; "Smoking": the individual has a smoking habit; "Health 1 (or 2)": the subjective health status is "very" (or "fairly") healthy; "Exercise1": the individual engages in light exercise at least once per week; "Exercise2": the individual engages in moderate or vigorous exercise at least once per week; "Y 2024": that the survey year is 2024. "Disease1" through "Disease6" are indicators for diseases under treatment or with lasting effects: "Disease1" is hypertension, "Disease2" is diabetes, "Disease3" is hyperlipidemia, "Disease4" is heat disease, "Disease 5" is kindey disease,, and "Disease 6" is other diseases. "ApoE4 hetero" and "ApoE4 homo" are dummy variables indicating carriers of the ApoE4 heterozygous and homozygous genotypes, respectively.

Table S2 (continued). Regression results (LDL-C)

LDL-C	Males						Females					
	Coef.	S.E.	t	P(t)	[95%	C.I.]	Coef.	S.E.	t	P(t)	[95%	C.I.]
1[Age<65] × Dietary 1	125.81	5.177	24.30	0.000	115.65	135.97	133.66	3.948	33.86	0.000	125.91	141.40
1[Age<65] × Dietary 2	127.57	5.362	23.79	0.000	117.05	138.10	128.68	4.196	30.67	0.000	120.45	136.91
1[Age<65] × Dietary 3	123.30	5.576	22.11	0.000	112.36	134.25	130.50	3.963	32.93	0.000	122.72	138.27
1[Age<65] × Dietary 4	132.94	6.592	20.17	0.000	120.00	145.88	133.53	4.348	30.71	0.000	125.00	142.06
1[Age<65] × Dietary 5	124.28	5.140	24.18	0.000	114.19	134.36	134.57	3.772	35.68	0.000	127.17	141.97
1[Age<65] × Dietary 6	132.45	7.441	17.80	0.000	117.85	147.06	136.92	4.614	29.67	0.000	127.86	145.97
1[Age≥65] × Dietary 1	120.06	4.859	24.71	0.000	110.53	129.60	127.32	4.485	28.39	0.000	118.52	136.12
1[Age≥65] × Dietary 2	120.73	4.846	24.91	0.000	111.21	130.24	135.88	4.548	29.88	0.000	126.96	144.80
1[Age≥65] × Dietary 3	124.72	4.955	25.17	0.000	114.99	134.44	131.53	4.096	32.11	0.000	123.50	139.57
1[Age≥65] × Dietary 4	126.40	5.023	25.17	0.000	116.54	136.25	134.19	3.991	33.62	0.000	126.36	142.01
1[Age≥65] × Dietary 5	124.18	4.793	25.91	0.000	114.77	133.58	129.70	3.798	34.15	0.000	122.25	137.15
1[Age≥65] × Dietary 6	126.03	5.499	22.92	0.000	115.24	136.82	128.68	4.249	30.28	0.000	120.34	137.01
School	-0.189	2.676	-0.071	0.944	-5.441	5.063	0.307	2.182	0.141	0.888	-3.974	4.587
Non cohabitants	-2.727	4.482	-0.608	0.543	-11.52	6.071	-3.736	2.780	-1.344	0.179	-9.189	1.717
Lifestyle change	1.963	1.511	1.299	0.194	-1.002	4.929	-0.189	1.179	-0.160	0.873	-2.501	2.123
Paid	-0.697	2.224	-0.313	0.754	-5.062	3.668	-0.545	1.803	-0.302	0.763	-4.082	2.992
Well off	-0.403	2.058	-0.196	0.845	-4.443	3.636	-0.094	1.538	-0.061	0.951	-3.111	2.923
Alcohol	0.196	2.328	0.084	0.933	-4.374	4.766	-0.867	1.680	-0.516	0.606	-4.163	2.429
Smoking	4.156	3.483	1.193	0.233	-2.680	10.99	1.859	4.235	0.439	0.661	-6.448	10.17
Healthy 1	-3.347	3.639	-0.920	0.358	-10.49	3.795	-0.358	3.016	-0.119	0.906	-6.274	5.558
Healthy 2	-1.202	2.658	-0.452	0.651	-6.419	4.016	1.806	2.364	0.764	0.445	-2.831	6.443
Exercise 1	-3.530	2.333	-1.513	0.131	-8.107	1.048	-0.942	1.793	-0.525	0.600	-4.460	2.576
Exercise 2	-0.762	1.970	-0.387	0.699	-4.628	3.104	-0.240	1.498	-0.160	0.873	-3.179	2.699
Disease 1	-3.330	2.220	-1.500	0.134	-7.686	1.027	0.669	2.217	0.302	0.763	-3.680	5.018
Disease 2	-4.698	3.363	-1.397	0.163	-11.30	1.903	-11.80	4.987	-2.366	0.018	-21.58	-2.016
Disease 3	-6.231	2.704	-2.304	0.021	-11.54	-0.923	-17.32	2.174	-7.967	0.000	-21.58	-13.06
Disease 4	-10.25	3.524	-2.908	0.004	-17.16	-3.332	-3.609	6.016	-0.600	0.549	-15.41	8.191
Disease 5	-4.376	2.776	-1.576	0.115	-9.823	1.073	6.977	7.645	0.913	0.362	-8.021	21.97
disease 6	-0.813	1.673	-0.486	0.627	-4.097	2.471	0.891	1.450	0.614	0.539	-1.955	3.736
ApoE4 hetero	5.808	2.868	2.025	0.043	0.179	11.44	6.480	2.695	2.404	0.016	1.193	11.77
ApoE4 homo	-13.72	15.35	-0.893	0.372	-43.85	16.42	5.267	8.871	0.594	0.553	-12.13	22.67
Y2024	1.082	0.919	1.177	0.240	-0.722	2.886	3.213	0.748	4.298	0.000	1.746	4.679
	N		915	F		2.402	N		1424	F		5.770
	R2 (Between)		0.098	P(F)		0.000	R2 (Between)		0.111	P(F)		0.000
	R2 (Within)		0.023	σ2(u)		594.48	R2 (Within)		0.054	σ2(u)		726.86
	R2 (Overall)		0.091	σ2(ε)		178.28	R2 (Overall)		0.105	σ2(ε)		175.46
	Log-likelihood		-3656.2	σ2(u)/σ2(u+ε)		0.769	Log-likelihood		-5696.8	σ2(u)/σ2(u+ε)		0.806

Note: "Dietary" denotes the dietary habit cluster: "Dietary 1" is a dummy variable indicating whether an individual belongs to dietary habit cluster 1, and so on. "School": an individual has 13 or more years of education; "Non cohabitants": the individual lives alone; "Lifestyle change": the individual experienced a major change in lifestyle during the past year; "Paid": the individual is engaged in paid work; "Well off": the household is financially comfortable; "Alcohol": the individual has a drinking habit; "Smoking": the individual has a smoking habit; "Health 1 (or 2)": the subjective health status is "very" (or "fairly") healthy; "Exercise1": the individual engages in light exercise at least once per week; "Exercise2": the individual engages in moderate or vigorous exercise at least once per week; "Y 2024": that the survey year is 2024. "Disease1" through "Disease6" are indicators for diseases under treatment or with lasting effects: "Disease1" is hypertension, "Disease2" is diabetes, "Disease3" is hyperlipidemia, "Disease4" is heat disease, "Disease 5" is kindey disease,, and "Disease 6" is other diseases. "ApoE4 hetero" and "ApoE4 homo" are dummy variables indicating carriers of the ApoE4 heterozygous and homozygous genotypes, respectively.

Table S2 (continued). Regression results (Na)

Na	Males						Females					
	Coef.	S.E.	t	P(t)	[95%	C.I.]	Coef.	S.E.	t	P(t)	[95%	C.I.]
1[Age<65] × Dietary 1	141.05	0.342	412.39	0.000	140.38	141.72	141.45	0.239	591.43	0.000	140.98	141.92
1[Age<65] × Dietary 2	140.36	0.360	389.40	0.000	139.65	141.06	141.33	0.257	550.72	0.000	140.83	141.84
1[Age<65] × Dietary 3	141.04	0.380	371.12	0.000	140.29	141.78	141.42	0.245	577.45	0.000	140.94	141.90
1[Age<65] × Dietary 4	141.06	0.457	309.03	0.000	140.17	141.96	141.33	0.273	517.89	0.000	140.79	141.86
1[Age<65] × Dietary 5	140.86	0.346	407.14	0.000	140.18	141.54	141.45	0.230	616.09	0.000	141.00	141.90
1[Age<65] × Dietary 6	141.10	0.519	271.68	0.000	140.08	142.12	141.49	0.286	495.22	0.000	140.93	142.05
1[Age≥65] × Dietary 1	140.89	0.326	432.00	0.000	140.25	141.53	141.71	0.279	508.10	0.000	141.16	142.26
1[Age≥65] × Dietary 2	140.61	0.326	431.26	0.000	139.97	141.25	141.07	0.275	513.97	0.000	140.54	141.61
1[Age≥65] × Dietary 3	140.57	0.333	422.36	0.000	139.92	141.22	141.40	0.255	555.68	0.000	140.90	141.90
1[Age≥65] × Dietary 4	140.72	0.340	414.08	0.000	140.05	141.38	141.21	0.245	576.85	0.000	140.73	141.69
1[Age≥65] × Dietary 5	140.82	0.323	435.83	0.000	140.19	141.45	141.31	0.233	607.22	0.000	140.85	141.77
1[Age≥65] × Dietary 6	140.98	0.385	366.65	0.000	140.22	141.73	141.27	0.261	540.38	0.000	140.76	141.78
School	0.112	0.149	0.751	0.453	-0.180	0.403	-0.046	0.110	-0.420	0.675	-0.261	0.169
Non cohabitants	0.023	0.279	0.081	0.936	-0.526	0.571	0.070	0.156	0.450	0.653	-0.236	0.376
Lifestyle change	0.050	0.116	0.433	0.665	-0.178	0.278	0.011	0.080	0.134	0.893	-0.147	0.168
Paid	0.271	0.145	1.867	0.062	-0.014	0.556	0.133	0.106	1.254	0.210	-0.075	0.340
Well off	-0.062	0.142	-0.440	0.660	-0.341	0.216	-0.150	0.097	-1.544	0.123	-0.341	0.041
Alcohol	0.135	0.144	0.942	0.347	-0.147	0.417	0.016	0.098	0.166	0.868	-0.175	0.208
Smoking	0.164	0.207	0.789	0.431	-0.244	0.571	0.134	0.232	0.576	0.565	-0.322	0.589
Healthy 1	-0.030	0.269	-0.110	0.912	-0.557	0.498	0.045	0.195	0.232	0.816	-0.337	0.427
Healthy 2	0.176	0.198	0.889	0.375	-0.213	0.566	0.191	0.152	1.255	0.210	-0.108	0.489
Exercise 1	-0.255	0.174	-1.463	0.144	-0.597	0.087	-0.174	0.118	-1.474	0.141	-0.405	0.058
Exercise 2	-0.053	0.144	-0.366	0.715	-0.335	0.230	-0.028	0.097	-0.289	0.773	-0.218	0.162
Disease 1	-0.005	0.146	-0.036	0.971	-0.291	0.280	0.041	0.132	0.310	0.757	-0.219	0.301
Disease 2	0.518	0.211	2.452	0.014	0.103	0.933	0.196	0.293	0.670	0.503	-0.378	0.771
Disease 3	0.179	0.181	0.989	0.323	-0.177	0.535	0.190	0.132	1.434	0.152	-0.070	0.449
Disease 4	0.456	0.247	1.849	0.065	-0.028	0.941	0.020	0.356	0.057	0.954	-0.677	0.718
Disease 5	-0.216	0.202	-1.066	0.287	-0.613	0.181	-1.363	0.511	-2.668	0.008	-2.365	-0.361
disease 6	-0.234	0.123	-1.903	0.057	-0.476	0.007	0.173	0.094	1.845	0.065	-0.011	0.356
ApoE4 hetero	-0.085	0.159	-0.536	0.592	-0.398	0.227	0.162	0.135	1.203	0.229	-0.102	0.427
ApoE4 homo	0.304	0.848	0.358	0.720	-1.361	1.969	0.240	0.445	0.538	0.590	-0.634	1.113
Y2024	1.435	0.080	17.87	0.000	1.278	1.593	1.212	0.056	21.76	0.000	1.103	1.321
	N		915	F		77.480	N		1424	F		331.980
	R2 (Between)		0.085	P(F)		0.000	R2 (Between)		0.050	P(F)		0.000
	R2 (Within)		0.424	σ2(u)		1.367	R2 (Within)		0.425	σ2(u)		1.540
	R2 (Overall)		0.205	σ2(ε)		1.401	R2 (Overall)		0.154	σ2(ε)		1.017
	Log-likelihood		-1439.1	σ2(u)/σ2(u+ε)		0.494	Log-likelihood		-2019.6	σ2(u)/σ2(u+ε)		0.602

Note: "Dietary" denotes the dietary habit cluster: "Dietary 1" is a dummy variable indicating whether an individual belongs to dietary habit cluster 1, and so on. "School": an individual has 13 or more years of education; "Non cohabitants": the individual lives alone; "Lifestyle change": the individual experienced a major change in lifestyle during the past year; "Paid": the individual is engaged in paid work; "Well off": the household is financially comfortable; "Alcohol": the individual has a drinking habit; "Smoking": the individual has a smoking habit; "Health 1 (or 2)": the subjective health status is "very" (or "fairly") healthy; "Exercise1": the individual engages in light exercise at least once per week; "Exercise2": the individual engages in moderate or vigorous exercise at least once per week; "Y 2024": that the survey year is 2024. "Disease1" through "Disease6" are indicators for diseases under treatment or with lasting effects: "Disease1" is hypertension, "Disease2" is diabetes, "Disease3" is hyperlipidemia, "Disease4" is heat disease, "Disease 5" is kindey disease,, and "Disease 6" is other diseases. "ApoE4 hetero" and "ApoE4 homo" are dummy variables indicating carriers of the ApoE4 heterozygous and homozygous genotypes, respectively.

Table S2 (continued). Regression results (Mg)

Mg	Males						Females					
	Coef.	S.E.	t	P(t)	[95%	C.I.]	Coef.	S.E.	t	P(t)	[95%	C.I.]
1[Age<65] × Dietary 1	2.114	0.033	64.78	0.000	2.050	2.178	2.133	0.024	88.92	0.000	2.086	2.180
1[Age<65] × Dietary 2	2.067	0.034	60.52	0.000	2.000	2.134	2.124	0.026	82.59	0.000	2.073	2.174
1[Age<65] × Dietary 3	2.028	0.036	56.53	0.000	1.958	2.098	2.109	0.025	86.03	0.000	2.061	2.157
1[Age<65] × Dietary 4	2.095	0.043	48.83	0.000	2.011	2.179	2.117	0.027	77.60	0.000	2.064	2.171
1[Age<65] × Dietary 5	2.105	0.033	64.21	0.000	2.040	2.169	2.139	0.023	92.96	0.000	2.094	2.184
1[Age<65] × Dietary 6	2.148	0.049	44.09	0.000	2.053	2.244	2.173	0.029	75.98	0.000	2.117	2.229
1[Age≥65] × Dietary 1	2.134	0.031	69.03	0.000	2.073	2.194	2.123	0.028	76.05	0.000	2.068	2.177
1[Age≥65] × Dietary 2	2.130	0.031	68.93	0.000	2.069	2.190	2.137	0.028	77.58	0.000	2.083	2.191
1[Age≥65] × Dietary 3	2.117	0.032	67.10	0.000	2.055	2.179	2.122	0.026	83.33	0.000	2.072	2.172
1[Age≥65] × Dietary 4	2.105	0.032	65.53	0.000	2.042	2.168	2.103	0.025	85.76	0.000	2.055	2.152
1[Age≥65] × Dietary 5	2.142	0.031	70.09	0.000	2.082	2.202	2.130	0.023	91.35	0.000	2.084	2.175
1[Age≥65] × Dietary 6	2.110	0.036	58.73	0.000	2.040	2.181	2.110	0.026	80.57	0.000	2.059	2.161
School	0.002	0.015	0.122	0.903	-0.028	0.031	-0.010	0.011	-0.863	0.389	-0.032	0.012
Non cohabitants	-0.035	0.027	-1.267	0.205	-0.088	0.019	0.002	0.016	0.124	0.901	-0.029	0.033
Lifestyle change	-0.016	0.011	-1.532	0.126	-0.037	0.005	-0.001	0.008	-0.165	0.869	-0.017	0.014
Paid	0.017	0.014	1.196	0.232	-0.011	0.044	-0.010	0.011	-0.887	0.375	-0.030	0.012
Well off	-0.001	0.013	-0.061	0.952	-0.027	0.026	-0.002	0.010	-0.185	0.853	-0.021	0.017
Alcohol	-0.006	0.014	-0.390	0.697	-0.033	0.022	0.017	0.010	1.720	0.086	-0.002	0.036
Smoking	-0.025	0.021	-1.208	0.227	-0.065	0.016	-0.051	0.024	-2.157	0.031	-0.097	-0.005
Healthy 1	0.048	0.025	1.938	0.053	-0.001	0.097	0.036	0.019	1.857	0.064	-0.002	0.074
Healthy 2	0.016	0.018	0.902	0.367	-0.019	0.052	0.028	0.015	1.858	0.064	-0.002	0.058
Exercise 1	-0.008	0.016	-0.514	0.607	-0.040	0.023	0.004	0.012	0.361	0.718	-0.019	0.027
Exercise 2	0.006	0.013	0.481	0.631	-0.020	0.033	-0.016	0.010	-1.680	0.093	-0.035	0.003
Disease 1	-0.033	0.014	-2.383	0.017	-0.061	-0.006	0.008	0.013	0.633	0.527	-0.018	0.035
Disease 2	-0.039	0.021	-1.883	0.060	-0.079	0.002	-0.052	0.030	-1.757	0.079	-0.110	0.006
Disease 3	0.046	0.017	2.662	0.008	0.012	0.080	0.023	0.013	1.696	0.090	-0.004	0.049
Disease 4	0.019	0.023	0.807	0.420	-0.027	0.064	-0.007	0.036	-0.204	0.838	-0.078	0.063
Disease 5	-0.009	0.019	-0.489	0.625	-0.046	0.028	0.000	0.051	0.008	0.993	-0.099	0.100
disease 6	-0.006	0.011	-0.551	0.582	-0.029	0.016	-0.007	0.009	-0.757	0.449	-0.025	0.011
ApoE4 hetero	-0.011	0.016	-0.652	0.515	-0.042	0.021	-0.006	0.014	-0.406	0.685	-0.033	0.021
ApoE4 homo	-0.042	0.086	-0.490	0.624	-0.210	0.126	-0.024	0.045	-0.531	0.595	-0.113	0.065
Y2024	0.033	0.007	4.708	0.000	0.019	0.046	0.024	0.006	4.404	0.000	0.013	0.035
	N		915	F		4.963	N		1424	F		8.667
	R2 (Between)		0.047	P(F)		0.000	R2 (Between)		0.034	P(F)		0.000
	R2 (Within)		0.112	σ2(u)		0.016	R2 (Within)		0.042	σ2(u)		0.017
	R2 (Overall)		0.063	σ2(ε)		0.010	R2 (Overall)		0.036	σ2(ε)		0.010
	Log-likelihood		811.8	σ2(u)/σ2(u+ε)		0.611	Log-likelihood		1286.6	σ2(u)/σ2(u+ε)		0.626

Note: "Dietary" denotes the dietary habit cluster: "Dietary 1" is a dummy variable indicating whether an individual belongs to dietary habit cluster 1, and so on. "School": an individual has 13 or more years of education; "Non cohabitants": the individual lives alone; "Lifestyle change": the individual experienced a major change in lifestyle during the past year; "Paid": the individual is engaged in paid work; "Well off": the household is financially comfortable; "Alcohol": the individual has a drinking habit; "Smoking": the individual has a smoking habit; "Health 1 (or 2)": the subjective health status is "very" (or "fairly") healthy; "Exercise1": the individual engages in light exercise at least once per week; "Exercise2": the individual engages in moderate or vigorous exercise at least once per week; "Y 2024": that the survey year is 2024. "Disease1" through "Disease6" are indicators for diseases under treatment or with lasting effects: "Disease1" is hypertension, "Disease2" is diabetes, "Disease3" is hyperlipidemia, "Disease4" is heat disease, "Disease 5" is kindey disease,, and "Disease 6" is other diseases. "ApoE4 hetero" and "ApoE4 homo" are dummy variables indicating carriers of the ApoE4 heterozygous and homozygous genotypes, respectively.

Table S2 (continued). Regression results (Ca)

Ca	Males						Females					
	Coef.	S.E.	t	P(t)	[95%	C.I.]	Coef.	S.E.	t	P(t)	[95%	C.I.]
1[Age<65] × Dietary 1	9.279	0.066	139.65	0.000	9.149	9.409	9.454	0.052	181.68	0.000	9.352	9.556
1[Age<65] × Dietary 2	9.212	0.070	130.76	0.000	9.073	9.350	9.406	0.056	167.94	0.000	9.296	9.516
1[Age<65] × Dietary 3	9.255	0.075	124.30	0.000	9.109	9.401	9.432	0.054	175.62	0.000	9.327	9.537
1[Age<65] × Dietary 4	9.373	0.090	104.54	0.000	9.197	9.549	9.476	0.060	157.57	0.000	9.358	9.594
1[Age<65] × Dietary 5	9.282	0.068	137.29	0.000	9.149	9.415	9.452	0.050	188.72	0.000	9.354	9.550
1[Age<65] × Dietary 6	9.330	0.102	91.41	0.000	9.129	9.530	9.533	0.063	152.44	0.000	9.410	9.655
1[Age≥65] × Dietary 1	9.249	0.064	145.10	0.000	9.123	9.374	9.421	0.061	153.98	0.000	9.301	9.541
1[Age≥65] × Dietary 2	9.329	0.064	146.48	0.000	9.204	9.454	9.409	0.060	158.15	0.000	9.292	9.526
1[Age≥65] × Dietary 3	9.308	0.065	143.20	0.000	9.180	9.435	9.496	0.056	169.87	0.000	9.386	9.605
1[Age≥65] × Dietary 4	9.372	0.067	140.79	0.000	9.241	9.502	9.443	0.054	176.65	0.000	9.338	9.548
1[Age≥65] × Dietary 5	9.316	0.063	147.25	0.000	9.192	9.440	9.475	0.051	186.25	0.000	9.375	9.575
1[Age≥65] × Dietary 6	9.294	0.076	122.34	0.000	9.145	9.443	9.418	0.057	164.96	0.000	9.306	9.530
School	-0.019	0.028	-0.674	0.501	-0.073	0.036	-0.016	0.023	-0.719	0.472	-0.060	0.028
Non cohabitants	-0.068	0.053	-1.277	0.202	-0.173	0.037	-0.062	0.033	-1.877	0.061	-0.126	0.003
Lifestyle change	0.002	0.023	0.104	0.917	-0.044	0.048	-0.034	0.018	-1.893	0.059	-0.070	0.001
Paid	-0.037	0.028	-1.312	0.190	-0.091	0.018	0.010	0.023	0.431	0.667	-0.035	0.054
Well off	-0.009	0.028	-0.315	0.753	-0.063	0.046	-0.011	0.021	-0.492	0.623	-0.052	0.031
Alcohol	0.017	0.027	0.617	0.537	-0.037	0.071	-0.011	0.021	-0.512	0.609	-0.052	0.030
Smoking	0.073	0.039	1.873	0.061	-0.004	0.150	0.059	0.049	1.203	0.229	-0.037	0.154
Healthy 1	0.005	0.054	0.085	0.932	-0.101	0.110	-0.050	0.043	-1.158	0.247	-0.135	0.035
Healthy 2	-0.026	0.040	-0.654	0.513	-0.104	0.052	-0.011	0.034	-0.331	0.741	-0.077	0.055
Exercise 1	0.036	0.035	1.028	0.304	-0.033	0.104	0.012	0.026	0.467	0.641	-0.039	0.064
Exercise 2	0.017	0.029	0.592	0.554	-0.039	0.073	-0.050	0.022	-2.345	0.019	-0.093	-0.008
Disease 1	0.014	0.028	0.502	0.616	-0.041	0.069	0.028	0.028	0.990	0.323	-0.028	0.084
Disease 2	0.160	0.040	3.974	0.000	0.081	0.239	0.030	0.063	0.480	0.632	-0.093	0.153
Disease 3	0.070	0.035	1.991	0.047	0.001	0.139	0.012	0.029	0.410	0.682	-0.044	0.068
Disease 4	0.042	0.048	0.876	0.381	-0.053	0.137	0.057	0.076	0.746	0.456	-0.093	0.206
Disease 5	-0.058	0.040	-1.442	0.150	-0.136	0.021	0.245	0.115	2.135	0.033	0.020	0.469
disease 6	-0.057	0.025	-2.308	0.021	-0.105	-0.008	-0.009	0.021	-0.446	0.656	-0.050	0.031
ApoE4 hetero	-0.025	0.030	-0.826	0.409	-0.083	0.034	0.006	0.028	0.215	0.830	-0.048	0.060
ApoE4 homo	-0.237	0.158	-1.498	0.135	-0.547	0.073	-0.077	0.091	-0.842	0.400	-0.256	0.102
Y2024	-0.137	0.017	-7.956	0.000	-0.170	-0.103	-0.143	0.013	-10.75	0.000	-0.169	-0.117
	N		915	F		9.738	N		1424	F		28.328
	R2 (Between)		0.094	P(F)		0.000	R2 (Between)		0.028	P(F)		0.000
	R2 (Within)		0.158	σ2(u)		0.040	R2 (Within)		0.171	σ2(u)		0.057
	R2 (Overall)		0.115	σ2(ε)		0.065	R2 (Overall)		0.068	σ2(ε)		0.059
	Log-likelihood		-30.7	σ2(u)/σ2(u+ε)		0.378	Log-likelihood		13.3	σ2(u)/σ2(u+ε)		0.491

Note: "Dietary" denotes the dietary habit cluster: "Dietary 1" is a dummy variable indicating whether an individual belongs to dietary habit cluster 1, and so on. "School": an individual has 13 or more years of education; "Non cohabitants": the individual lives alone; "Lifestyle change": the individual experienced a major change in lifestyle during the past year; "Paid": the individual is engaged in paid work; "Well off": the household is financially comfortable; "Alcohol": the individual has a drinking habit; "Smoking": the individual has a smoking habit; "Health 1 (or 2)": the subjective health status is "very" (or "fairly") healthy; "Exercise1": the individual engages in light exercise at least once per week; "Exercise2": the individual engages in moderate or vigorous exercise at least once per week; "Y 2024": that the survey year is 2024. "Disease1" through "Disease6" are indicators for diseases under treatment or with lasting effects: "Disease1" is hypertension, "Disease2" is diabetes, "Disease3" is hyperlipidemia, "Disease4" is heat disease, "Disease 5" is kindey disease,, and "Disease 6" is other diseases. "ApoE4 hetero" and "ApoE4 homo" are dummy variables indicating carriers of the ApoE4 heterozygous and homozygous genotypes, respectively.

Table S2 (continued). Regression results (Fe)

Fe	Males						Females					
	Coef.	S.E.	t	P(t)	[95%	C.I.]	Coef.	S.E.	t	P(t)	[95%	C.I.]
1[Age<65] × Dietary 1	101.63	6.430	15.81	0.000	89.01	114.25	89.96	4.250	21.17	0.000	81.62	98.30
1[Age<65] × Dietary 2	92.50	6.842	13.52	0.000	79.08	105.93	92.86	4.586	20.25	0.000	83.87	101.86
1[Age<65] × Dietary 3	93.85	7.240	12.96	0.000	79.64	108.05	91.69	4.416	20.76	0.000	83.03	100.35
1[Age<65] × Dietary 4	101.09	8.727	11.58	0.000	83.96	118.22	85.49	4.962	17.23	0.000	75.76	95.23
1[Age<65] × Dietary 5	92.20	6.565	14.05	0.000	79.32	105.08	93.15	4.101	22.71	0.000	85.11	101.19
1[Age<65] × Dietary 6	90.63	9.933	9.124	0.000	71.14	110.13	92.12	5.128	17.96	0.000	82.06	102.18
1[Age≥65] × Dietary 1	94.02	6.190	15.19	0.000	81.87	106.16	89.55	5.028	17.81	0.000	79.69	99.41
1[Age≥65] × Dietary 2	90.90	6.181	14.71	0.000	78.77	103.04	92.42	4.841	19.09	0.000	82.93	101.92
1[Age≥65] × Dietary 3	89.47	6.308	14.18	0.000	77.09	101.85	90.90	4.603	19.75	0.000	81.87	99.93
1[Age≥65] × Dietary 4	100.98	6.472	15.60	0.000	88.28	113.69	88.58	4.377	20.24	0.000	80.00	97.17
1[Age≥65] × Dietary 5	98.93	6.151	16.08	0.000	86.86	111.00	91.50	4.171	21.94	0.000	83.32	99.68
1[Age≥65] × Dietary 6	87.51	7.421	11.79	0.000	72.94	102.07	87.85	4.673	18.80	0.000	78.69	97.02
School	-0.198	2.622	-0.076	0.940	-5.345	4.948	1.614	1.756	0.919	0.358	-1.831	5.058
Non cohabitants	-0.025	5.104	-0.005	0.996	-10.04	9.993	-3.382	2.615	-1.293	0.196	-8.513	1.748
Lifestyle change	2.790	2.314	1.206	0.228	-1.751	7.331	0.933	1.535	0.607	0.544	-2.079	3.944
Paid	-2.645	2.685	-0.985	0.325	-7.915	2.625	-0.017	1.818	-0.010	0.992	-3.583	3.548
Well off	1.687	2.680	0.629	0.529	-3.574	6.948	1.016	1.750	0.581	0.562	-2.416	4.448
Alcohol	8.806	2.612	3.372	0.001	3.680	13.93	2.536	1.668	1.520	0.129	-0.737	5.809
Smoking	4.657	3.731	1.248	0.212	-2.666	11.98	10.46	3.853	2.715	0.007	2.904	18.02
Healthy 1	14.48	5.248	2.759	0.006	4.177	24.78	1.291	3.566	0.362	0.717	-5.704	8.286
Healthy 2	5.507	3.891	1.415	0.157	-2.130	13.14	3.801	2.769	1.372	0.170	-1.632	9.233
Exercise 1	3.257	3.432	0.949	0.343	-3.478	9.992	2.421	2.188	1.107	0.269	-1.871	6.712
Exercise 2	-2.645	2.802	-0.944	0.345	-8.144	2.854	-1.685	1.780	-0.947	0.344	-5.177	1.807
Disease 1	1.224	2.699	0.454	0.650	-4.073	6.520	-0.826	2.295	-0.360	0.719	-5.327	3.676
Disease 2	-5.011	3.860	-1.298	0.195	-12.59	2.566	-5.130	5.040	-1.018	0.309	-15.02	4.757
Disease 3	-0.417	3.389	-0.123	0.902	-7.068	6.235	2.540	2.321	1.094	0.274	-2.013	7.093
Disease 4	-5.015	4.699	-1.067	0.286	-14.24	4.208	1.488	6.122	0.243	0.808	-10.52	13.50
Disease 5	-4.237	3.914	-1.082	0.279	-11.92	3.446	-9.054	9.575	-0.946	0.345	-27.84	9.728
disease 6	0.846	2.398	0.353	0.724	-3.860	5.551	1.473	1.710	0.861	0.389	-1.881	4.826
ApoE4 hetero	-3.236	2.813	-1.150	0.250	-8.757	2.285	0.470	2.157	0.218	0.828	-3.761	4.700
ApoE4 homo	0.027	14.95	0.002	0.999	-29.32	29.37	-5.594	7.137	-0.784	0.433	-19.60	8.407
Y2024	-2.716	1.759	-1.544	0.123	-6.169	0.737	-2.398	1.182	-2.028	0.043	-4.718	-0.079
	N		915	F		1.890	N		1424	F		1.242
	R2 (Between)		0.080	P(F)		0.003	R2 (Between)		0.033	P(F)		0.170
	R2 (Within)		0.036	σ2(u)		300.71	R2 (Within)		0.017	σ2(u)		287.15
	R2 (Overall)		0.066	σ2(ε)		682.94	R2 (Overall)		0.027	σ2(ε)		471.81
	Log-likelihood		-4268.7	σ2(u)/σ2(u+ε)		0.306	Log-likelihood		-6385.5	σ2(u)/σ2(u+ε)		0.378

Note: "Dietary" denotes the dietary habit cluster: "Dietary 1" is a dummy variable indicating whether an individual belongs to dietary habit cluster 1, and so on. "School": an individual has 13 or more years of education; "Non cohabitants": the individual lives alone; "Lifestyle change": the individual experienced a major change in lifestyle during the past year; "Paid": the individual is engaged in paid work; "Well off": the household is financially comfortable; "Alcohol": the individual has a drinking habit; "Smoking": the individual has a smoking habit; "Health 1 (or 2)": the subjective health status is "very" (or "fairly") healthy; "Exercise1": the individual engages in light exercise at least once per week; "Exercise2": the individual engages in moderate or vigorous exercise at least once per week; "Y 2024": that the survey year is 2024. "Disease1" through "Disease6" are indicators for diseases under treatment or with lasting effects: "Disease1" is hypertension, "Disease2" is diabetes, "Disease3" is hyperlipidemia, "Disease4" is heat disease, "Disease 5" is kindey disease,, and "Disease 6" is other diseases. "ApoE4 hetero" and "ApoE4 homo" are dummy variables indicating carriers of the ApoE4 heterozygous and homozygous genotypes, respectively.

Table S2 (continued). Regression results (High-sensitivity C-reactive protein)

Hs-CRP	Males						Females					
	Coef.	S.E.	t	P(t)	[95%	C.I.]	Coef.	S.E.	t	P(t)	[95%	C.I.]
1[Age<65] × Dietary 1	0.119	0.055	2.152	0.032	0.010	0.227	0.293	0.165	1.776	0.076	-0.031	0.618
1[Age<65] × Dietary 2	0.102	0.059	1.711	0.087	-0.015	0.218	0.241	0.179	1.345	0.179	-0.111	0.593
1[Age<65] × Dietary 3	0.110	0.063	1.742	0.082	-0.014	0.233	0.234	0.174	1.343	0.179	-0.108	0.576
1[Age<65] × Dietary 4	0.216	0.076	2.842	0.005	0.067	0.365	0.293	0.197	1.484	0.138	-0.094	0.679
1[Age<65] × Dietary 5	0.117	0.057	2.053	0.040	0.005	0.228	0.243	0.161	1.515	0.130	-0.072	0.558
1[Age<65] × Dietary 6	0.176	0.086	2.038	0.042	0.007	0.345	0.465	0.201	2.319	0.021	0.072	0.859
1[Age≥65] × Dietary 1	0.116	0.054	2.153	0.032	0.010	0.221	0.235	0.198	1.189	0.235	-0.153	0.623
1[Age≥65] × Dietary 2	0.147	0.054	2.751	0.006	0.042	0.252	0.256	0.186	1.378	0.168	-0.108	0.621
1[Age≥65] × Dietary 3	0.186	0.055	3.401	0.001	0.079	0.293	0.651	0.183	3.567	0.000	0.293	1.010
1[Age≥65] × Dietary 4	0.233	0.056	4.137	0.000	0.122	0.343	0.191	0.171	1.116	0.265	-0.144	0.526
1[Age≥65] × Dietary 5	0.145	0.054	2.711	0.007	0.040	0.250	0.204	0.164	1.246	0.213	-0.117	0.525
1[Age≥65] × Dietary 6	0.162	0.065	2.485	0.013	0.034	0.290	0.244	0.182	1.343	0.179	-0.112	0.601
School	0.022	0.022	1.027	0.305	-0.020	0.064	0.063	0.062	1.024	0.306	-0.058	0.184
Non cohabitants	0.032	0.043	0.737	0.461	-0.053	0.116	-0.057	0.095	-0.599	0.549	-0.243	0.130
Lifestyle change	0.006	0.021	0.300	0.765	-0.035	0.047	-0.091	0.064	-1.418	0.157	-0.217	0.035
Paid	-0.040	0.023	-1.758	0.079	-0.084	0.005	-0.089	0.067	-1.315	0.189	-0.221	0.044
Well off	0.001	0.023	0.056	0.956	-0.044	0.047	0.040	0.068	0.595	0.552	-0.093	0.174
Alcohol	-0.054	0.022	-2.478	0.013	-0.097	-0.011	-0.037	0.062	-0.593	0.553	-0.157	0.084
Smoking	0.021	0.031	0.690	0.491	-0.039	0.082	-0.017	0.139	-0.123	0.902	-0.290	0.256
Healthy 1	0.060	0.046	1.289	0.198	-0.031	0.151	-0.069	0.142	-0.489	0.625	-0.347	0.208
Healthy 2	0.041	0.035	1.188	0.235	-0.027	0.109	-0.008	0.109	-0.073	0.942	-0.222	0.206
Exercise 1	-0.024	0.031	-0.780	0.436	-0.084	0.036	0.010	0.088	0.110	0.912	-0.163	0.183
Exercise 2	0.014	0.025	0.585	0.559	-0.034	0.063	0.079	0.071	1.109	0.268	-0.061	0.218
Disease 1	0.014	0.023	0.624	0.533	-0.031	0.059	-0.002	0.086	-0.020	0.984	-0.170	0.167
Disease 2	0.021	0.032	0.644	0.520	-0.043	0.084	-0.005	0.188	-0.029	0.977	-0.374	0.363
Disease 3	0.029	0.029	1.014	0.311	-0.027	0.086	-0.008	0.088	-0.092	0.927	-0.180	0.164
Disease 4	0.021	0.041	0.526	0.599	-0.058	0.101	0.049	0.227	0.214	0.831	-0.397	0.495
Disease 5	-0.066	0.034	-1.914	0.056	-0.133	0.002	0.039	0.389	0.101	0.919	-0.724	0.802
disease 6	0.017	0.021	0.781	0.435	-0.025	0.058	-0.065	0.068	-0.957	0.339	-0.197	0.068
ApoE4 hetero	-0.062	0.023	-2.689	0.007	-0.107	-0.017	-0.107	0.076	-1.420	0.156	-0.256	0.041
ApoE4 homo	-0.116	0.122	-0.951	0.342	-0.356	0.124	-0.154	0.252	-0.614	0.540	-0.648	0.339
Y2024	-0.023	0.018	-1.305	0.192	-0.058	0.012	-0.116	0.061	-1.907	0.057	-0.234	0.003
	N		915	F		1.615	N		1424	F		0.984
	R2 (Between)		0.096	P(F)		0.019	R2 (Between)		0.036	P(F)		0.493
	R2 (Within)		0.004	σ2(u)		0.008	R2 (Within)		0.005	σ2(u)		0.000
	R2 (Overall)		0.059	σ2(ε)		0.070	R2 (Overall)		0.021	σ2(ε)		1.254
	Log-likelihood		-63.4	σ2(u)/σ2(u+ε)		0.103	Log-likelihood		-2173.0	σ2(u)/σ2(u+ε)		0.000

Note: "Dietary" denotes the dietary habit cluster: "Dietary 1" is a dummy variable indicating whether an individual belongs to dietary habit cluster 1, and so on. "School": an individual has 13 or more years of education; "Non cohabitants": the individual lives alone; "Lifestyle change": the individual experienced a major change in lifestyle during the past year; "Paid": the individual is engaged in paid work; "Well off": the household is financially comfortable; "Alcohol": the individual has a drinking habit; "Smoking": the individual has a smoking habit; "Health 1 (or 2)": the subjective health status is "very" (or "fairly") healthy; "Exercise1": the individual engages in light exercise at least once per week; "Exercise2": the individual engages in moderate or vigorous exercise at least once per week; "Y 2024": that the survey year is 2024. "Disease1" through "Disease6" are indicators for diseases under treatment or with lasting effects: "Disease1" is hypertension, "Disease2" is diabetes, "Disease3" is hyperlipidemia, "Disease4" is heat disease, "Disease 5" is kindey disease,, and "Disease 6" is other diseases. "ApoE4 hetero" and "ApoE4 homo" are dummy variables indicating carriers of the ApoE4 heterozygous and homozygous genotypes, respectively.

Table S2 (continued). Regression results (MMSE total score)

MMSE total score	Males						Females					
	Coef.	S.E.	t	P(t)	[95%	C.I.]	Coef.	S.E.	t	P(t)	[95%	C.I.]
1[Age<65] × Dietary 1	27.30	0.370	73.87	0.000	26.58	28.03	27.67	0.242	114.39	0.000	27.20	28.15
1[Age<65] × Dietary 2	27.07	0.390	69.45	0.000	26.30	27.83	27.48	0.261	105.39	0.000	26.97	27.99
1[Age<65] × Dietary 3	27.19	0.411	66.14	0.000	26.38	28.00	27.89	0.251	111.27	0.000	27.40	28.38
1[Age<65] × Dietary 4	26.85	0.494	54.36	0.000	25.88	27.82	27.56	0.281	98.00	0.000	27.01	28.11
1[Age<65] × Dietary 5	26.96	0.374	72.05	0.000	26.22	27.69	27.90	0.233	119.66	0.000	27.44	28.36
1[Age<65] × Dietary 6	27.98	0.562	49.77	0.000	26.87	29.08	28.00	0.291	96.09	0.000	27.43	28.57
1[Age≥65] × Dietary 1	26.66	0.353	75.61	0.000	25.97	27.36	27.21	0.285	95.32	0.000	26.65	27.77
1[Age≥65] × Dietary 2	26.75	0.353	75.87	0.000	26.06	27.44	27.20	0.276	98.54	0.000	26.66	27.74
1[Age≥65] × Dietary 3	26.85	0.360	74.59	0.000	26.14	27.55	27.21	0.261	104.22	0.000	26.70	27.72
1[Age≥65] × Dietary 4	27.06	0.368	73.62	0.000	26.34	27.78	27.41	0.249	110.13	0.000	26.92	27.90
1[Age≥65] × Dietary 5	27.07	0.350	77.46	0.000	26.38	27.76	27.19	0.237	114.69	0.000	26.72	27.65
1[Age≥65] × Dietary 6	26.38	0.416	63.36	0.000	25.56	27.20	27.25	0.266	102.54	0.000	26.73	27.78
School	0.618	0.160	3.869	0.000	0.304	0.931	0.358	0.102	3.518	0.000	0.159	0.558
Non cohabitants	-0.178	0.301	-0.590	0.555	-0.769	0.413	0.137	0.151	0.908	0.364	-0.159	0.432
Lifestyle change	0.153	0.126	1.216	0.224	-0.094	0.401	0.019	0.086	0.223	0.824	-0.150	0.188
Paid	0.022	0.157	0.143	0.887	-0.285	0.330	-0.066	0.104	-0.629	0.529	-0.270	0.139
Well off	-0.013	0.153	-0.083	0.934	-0.314	0.288	0.084	0.099	0.849	0.396	-0.111	0.280
Alcohol	0.242	0.155	1.564	0.118	-0.062	0.546	0.248	0.096	2.585	0.010	0.060	0.435
Smoking	-0.460	0.223	-2.059	0.040	-0.898	-0.022	-0.039	0.222	-0.173	0.863	-0.474	0.397
Healthy 1	-0.273	0.291	-0.935	0.350	-0.844	0.299	0.140	0.202	0.693	0.488	-0.256	0.536
Healthy 2	-0.013	0.215	-0.062	0.950	-0.436	0.409	0.291	0.157	1.850	0.065	-0.018	0.599
Exercise 1	0.225	0.189	1.188	0.235	-0.147	0.596	0.128	0.124	1.033	0.302	-0.115	0.370
Exercise 2	-0.278	0.156	-1.783	0.075	-0.585	0.028	-0.031	0.101	-0.310	0.757	-0.229	0.166
Disease 1	0.023	0.157	0.143	0.886	-0.286	0.331	-0.012	0.131	-0.093	0.926	-0.270	0.245
Disease 2	-0.147	0.228	-0.645	0.519	-0.594	0.300	-0.073	0.289	-0.254	0.800	-0.640	0.493
Disease 3	0.192	0.196	0.979	0.328	-0.193	0.577	0.131	0.133	0.991	0.322	-0.129	0.391
Disease 4	-0.042	0.267	-0.157	0.876	-0.566	0.482	-0.029	0.351	-0.082	0.934	-0.717	0.659
Disease 5	0.238	0.219	1.087	0.277	-0.192	0.668	0.268	0.540	0.496	0.620	-0.791	1.327
disease 6	0.050	0.134	0.373	0.710	-0.212	0.312	0.139	0.097	1.433	0.152	-0.051	0.329
ApoE4 hetero	0.042	0.171	0.243	0.808	-0.294	0.378	-0.184	0.125	-1.469	0.142	-0.429	0.062
ApoE4 homo	-2.335	0.912	-2.561	0.011	-4.124	-0.545	0.027	0.414	0.066	0.947	-0.785	0.839
Y2024	0.612	0.088	6.969	0.000	0.440	0.784	0.532	0.065	8.208	0.000	0.405	0.659
	N		915	F		5.411	N		1424	F		6.252
	R2 (Between)		0.104	P(F)		0.000	R2 (Between)		0.099	P(F)		0.000
	R2 (Within)		0.127	σ2(u)		1.554	R2 (Within)		0.086	σ2(u)		1.066
	R2 (Overall)		0.108	σ2(ε)		1.680	R2 (Overall)		0.089	σ2(ε)		1.419
	Log-likelihood		-1521.0	σ2(u)/σ2(u+ε)		0.480	Log-likelihood		-2246.9	σ2(u)/σ2(u+ε)		0.429

Note: "Dietary" denotes the dietary habit cluster: "Dietary 1" is a dummy variable indicating whether an individual belongs to dietary habit cluster 1, and so on. "School": an individual has 13 or more years of education; "Non cohabitants": the individual lives alone; "Lifestyle change": the individual experienced a major change in lifestyle during the past year; "Paid": the individual is engaged in paid work; "Well off": the household is financially comfortable; "Alcohol": the individual has a drinking habit; "Smoking": the individual has a smoking habit; "Health 1 (or 2)": the subjective health status is "very" (or "fairly") healthy; "Exercise1": the individual engages in light exercise at least once per week; "Exercise2": the individual engages in moderate or vigorous exercise at least once per week; "Y 2024": that the survey year is 2024. "Disease1" through "Disease6" are indicators for diseases under treatment or with lasting effects: "Disease1" is hypertension, "Disease2" is diabetes, "Disease3" is hyperlipidemia, "Disease4" is heat disease, "Disease 5" is kindey disease,, and "Disease 6" is other diseases. "ApoE4 hetero" and "ApoE4 homo" are dummy variables indicating carriers of the ApoE4 heterozygous and homozygous genotypes, respectively.

Table S2 (continued). Regression results (Moca-J total score)

Moca-J total score	Males						Females					
	Coef.	S.E.	t	P(t)	[95%	C.I.]	Coef.	S.E.	t	P(t)	[95%	C.I.]
1[Age<65] × Dietary 1	24.80	0.562	44.11	0.000	23.70	25.90	25.73	0.389	66.11	0.000	24.97	26.50
1[Age<65] × Dietary 2	24.11	0.591	40.80	0.000	22.95	25.27	25.92	0.418	62.00	0.000	25.10	26.74
1[Age<65] × Dietary 3	25.05	0.622	40.26	0.000	23.83	26.27	25.83	0.399	64.68	0.000	25.05	26.61
1[Age<65] × Dietary 4	23.89	0.746	32.02	0.000	22.42	25.35	25.59	0.445	57.45	0.000	24.72	26.46
1[Age<65] × Dietary 5	24.56	0.567	43.31	0.000	23.45	25.68	25.53	0.374	68.28	0.000	24.80	26.26
1[Age<65] × Dietary 6	25.21	0.849	29.71	0.000	23.54	26.87	25.40	0.466	54.55	0.000	24.49	26.32
1[Age≥65] × Dietary 1	22.81	0.535	42.65	0.000	21.76	23.86	24.47	0.455	53.79	0.000	23.57	25.36
1[Age≥65] × Dietary 2	23.10	0.535	43.21	0.000	22.05	24.15	23.77	0.446	53.25	0.000	22.90	24.65
1[Age≥65] × Dietary 3	23.72	0.546	43.45	0.000	22.64	24.79	24.51	0.415	59.06	0.000	23.70	25.33
1[Age≥65] × Dietary 4	23.04	0.557	41.40	0.000	21.95	24.13	24.21	0.399	60.71	0.000	23.43	24.99
1[Age≥65] × Dietary 5	23.06	0.529	43.57	0.000	22.02	24.10	24.35	0.379	64.22	0.000	23.61	25.09
1[Age≥65] × Dietary 6	22.53	0.627	35.94	0.000	21.30	23.76	23.95	0.426	56.23	0.000	23.11	24.78
School	0.243	0.249	0.974	0.330	-0.247	0.732	-0.375	0.176	-2.132	0.033	-0.720	-0.030
Non cohabitants	-0.275	0.463	-0.593	0.554	-1.184	0.635	0.654	0.252	2.593	0.010	0.159	1.148
Lifestyle change	0.350	0.187	1.870	0.062	-0.017	0.717	-0.140	0.132	-1.059	0.290	-0.399	0.119
Paid	-0.078	0.239	-0.325	0.745	-0.548	0.392	0.255	0.172	1.484	0.138	-0.082	0.591
Well off	0.338	0.232	1.454	0.146	-0.118	0.794	0.060	0.159	0.377	0.706	-0.251	0.371
Alcohol	0.244	0.239	1.023	0.307	-0.224	0.712	0.163	0.158	1.029	0.304	-0.148	0.473
Smoking	-0.370	0.346	-1.071	0.285	-1.049	0.309	-0.551	0.375	-1.470	0.142	-1.286	0.184
Healthy 1	-0.339	0.436	-0.777	0.437	-1.195	0.517	-0.049	0.318	-0.155	0.877	-0.674	0.575
Healthy 2	0.237	0.321	0.736	0.462	-0.394	0.867	0.178	0.248	0.715	0.475	-0.310	0.665
Exercise 1	0.241	0.282	0.854	0.393	-0.313	0.796	0.131	0.193	0.679	0.497	-0.248	0.510
Exercise 2	-0.509	0.234	-2.175	0.030	-0.968	-0.050	0.167	0.159	1.052	0.293	-0.144	0.478
Disease 1	0.095	0.240	0.396	0.692	-0.376	0.566	-0.013	0.215	-0.060	0.952	-0.435	0.409
Disease 2	-0.299	0.350	-0.854	0.394	-0.986	0.389	-0.361	0.475	-0.760	0.448	-1.292	0.571
Disease 3	0.177	0.298	0.592	0.554	-0.409	0.762	0.114	0.215	0.530	0.597	-0.308	0.536
Disease 4	-0.026	0.404	-0.064	0.949	-0.818	0.766	0.222	0.577	0.384	0.701	-0.910	1.353
Disease 5	0.838	0.329	2.549	0.011	0.193	1.484	-0.587	0.837	-0.701	0.484	-2.229	1.056
disease 6	0.392	0.200	1.962	0.050	0.000	0.785	0.444	0.153	2.901	0.004	0.144	0.744
ApoE4 hetero	-0.176	0.267	-0.657	0.511	-0.700	0.349	-0.355	0.217	-1.641	0.101	-0.780	0.069
ApoE4 homo	-2.694	1.426	-1.890	0.059	-5.492	0.103	-0.431	0.715	-0.603	0.547	-1.833	0.971
Y2024	0.496	0.127	3.923	0.000	0.248	0.745	0.725	0.092	7.841	0.000	0.544	0.906
	N		915	F		4.499	N		1424	F		6.613
	R2 (Between)		0.188	P(F)		0.000	R2 (Between)		0.131	P(F)		0.000
	R2 (Within)		0.037	σ2(u)		3.981	R2 (Within)		0.094	σ2(u)		3.915
	R2 (Overall)		0.158	σ2(ε)		3.349	R2 (Overall)		0.122	σ2(ε)		2.843
	Log-likelihood		-1852.9	σ2(u)/σ2(u+ε)		0.543	Log-likelihood		-2742.9	σ2(u)/σ2(u+ε)		0.579

Note: "Dietary" denotes the dietary habit cluster: "Dietary 1" is a dummy variable indicating whether an individual belongs to dietary habit cluster 1, and so on. "School": an individual has 13 or more years of education; "Non cohabitants": the individual lives alone; "Lifestyle change": the individual experienced a major change in lifestyle during the past year; "Paid": the individual is engaged in paid work; "Well off": the household is financially comfortable; "Alcohol": the individual has a drinking habit; "Smoking": the individual has a smoking habit; "Health 1 (or 2)": the subjective health status is "very" (or "fairly") healthy; "Exercise1": the individual engages in light exercise at least once per week; "Exercise2": the individual engages in moderate or vigorous exercise at least once per week; "Y 2024": that the survey year is 2024. "Disease1" through "Disease6" are indicators for diseases under treatment or with lasting effects: "Disease1" is hypertension, "Disease2" is diabetes, "Disease3" is hyperlipidemia, "Disease4" is heat disease, "Disease 5" is kindey disease,, and "Disease 6" is other diseases. "ApoE4 hetero" and "ApoE4 homo" are dummy variables indicating carriers of the ApoE4 heterozygous and homozygous genotypes, respectively.

Table S2 (continued). Regression results (Aβ-CM)

Aβ-CM	Males						Females					
	Coef.	S.E.	t	P(t)	[95%	C.I.]	Coef.	S.E.	t	P(t)	[95%	C.I.]
1[Age<65] × Dietary 1	-0.236	0.127	-1.855	0.064	-0.486	0.014	-0.219	0.099	-2.226	0.026	-0.413	-0.026
1[Age<65] × Dietary 2	0.002	0.134	0.018	0.986	-0.260	0.265	-0.101	0.106	-0.959	0.338	-0.309	0.106
1[Age<65] × Dietary 3	-0.181	0.141	-1.281	0.201	-0.457	0.096	-0.149	0.101	-1.478	0.140	-0.346	0.049
1[Age<65] × Dietary 4	-0.271	0.169	-1.601	0.110	-0.602	0.061	-0.205	0.112	-1.827	0.068	-0.425	0.015
1[Age<65] × Dietary 5	-0.161	0.129	-1.255	0.210	-0.413	0.091	-0.181	0.095	-1.918	0.055	-0.367	0.004
1[Age<65] × Dietary 6	-0.164	0.192	-0.854	0.393	-0.541	0.213	-0.132	0.118	-1.122	0.262	-0.362	0.099
1[Age≥65] × Dietary 1	-0.062	0.121	-0.510	0.610	-0.300	0.176	0.147	0.115	1.284	0.199	-0.078	0.372
1[Age≥65] × Dietary 2	0.189	0.121	1.562	0.119	-0.049	0.427	0.150	0.113	1.327	0.185	-0.072	0.372
1[Age≥65] × Dietary 3	-0.097	0.124	-0.783	0.434	-0.339	0.146	0.026	0.105	0.251	0.802	-0.179	0.231
1[Age≥65] × Dietary 4	-0.074	0.126	-0.584	0.560	-0.321	0.174	0.029	0.101	0.286	0.775	-0.169	0.227
1[Age≥65] × Dietary 5	-0.015	0.120	-0.125	0.901	-0.250	0.220	0.046	0.096	0.477	0.634	-0.142	0.234
1[Age≥65] × Dietary 6	0.159	0.142	1.117	0.264	-0.120	0.437	0.087	0.108	0.806	0.420	-0.124	0.298
School	0.054	0.057	0.963	0.336	-0.057	0.165	-0.078	0.046	-1.704	0.089	-0.168	0.012
Non cohabitants	-0.024	0.105	-0.229	0.819	-0.230	0.182	-0.064	0.065	-0.982	0.326	-0.191	0.064
Lifestyle change	-0.018	0.042	-0.421	0.674	-0.101	0.065	-0.046	0.033	-1.403	0.161	-0.110	0.018
Paid	0.071	0.054	1.310	0.190	-0.035	0.178	-0.024	0.044	-0.539	0.590	-0.110	0.062
Well off	-0.009	0.053	-0.166	0.869	-0.112	0.095	0.053	0.040	1.321	0.187	-0.026	0.131
Alcohol	0.042	0.054	0.773	0.440	-0.064	0.148	0.018	0.041	0.456	0.649	-0.061	0.098
Smoking	-0.065	0.078	-0.834	0.404	-0.219	0.088	0.053	0.097	0.549	0.583	-0.137	0.243
Healthy 1	-0.085	0.099	-0.864	0.388	-0.279	0.109	-0.079	0.080	-0.982	0.326	-0.235	0.078
Healthy 2	0.052	0.073	0.713	0.476	-0.091	0.195	-0.015	0.062	-0.243	0.808	-0.137	0.107
Exercise 1	-0.006	0.064	-0.087	0.931	-0.131	0.120	0.033	0.048	0.675	0.500	-0.062	0.127
Exercise 2	0.059	0.053	1.106	0.269	-0.045	0.163	-0.019	0.040	-0.477	0.633	-0.097	0.059
Disease 1	0.001	0.054	0.020	0.984	-0.106	0.108	-0.055	0.055	-1.012	0.312	-0.163	0.052
Disease 2	0.042	0.079	0.532	0.595	-0.114	0.198	0.181	0.121	1.497	0.135	-0.056	0.419
Disease 3	-0.114	0.068	-1.692	0.091	-0.247	0.018	-0.015	0.055	-0.266	0.791	-0.122	0.093
Disease 4	0.013	0.091	0.143	0.886	-0.166	0.192	0.059	0.147	0.404	0.687	-0.229	0.348
Disease 5	0.044	0.075	0.593	0.553	-0.102	0.190	0.278	0.209	1.330	0.184	-0.132	0.687
disease 6	0.073	0.045	1.616	0.106	-0.016	0.162	0.004	0.038	0.090	0.928	-0.072	0.079
ApoE4 hetero	0.203	0.061	3.349	0.001	0.084	0.322	0.338	0.057	5.987	0.000	0.228	0.449
ApoE4 homo	1.468	0.323	4.544	0.000	0.834	2.102	0.919	0.186	4.931	0.000	0.554	1.285
Y2024	-0.395	0.029	-13.81	0.000	-0.452	-0.339	-0.420	0.022	-18.73	0.000	-0.464	-0.376
	N		915	F		8.884	N		1424	F		14.920
	R2 (Between)		0.110	P(F)		0.000	R2 (Between)		0.132	P(F)		0.000
	R2 (Within)		0.334	σ2(u)		0.215	R2 (Within)		0.343	σ2(u)		0.279
	R2 (Overall)		0.174	σ2(ε)		0.180	R2 (Overall)		0.178	σ2(ε)		0.166
	Log-likelihood		-493.0	σ2(u)/σ2(u+ε)		0.545	Log-likelihood		-723.2	σ2(u)/σ2(u+ε)		0.628

Note: "Dietary" denotes the dietary habit cluster: "Dietary 1" is a dummy variable indicating whether an individual belongs to dietary habit cluster 1, and so on. "School": an individual has 13 or more years of education; "Non cohabitants": the individual lives alone; "Lifestyle change": the individual experienced a major change in lifestyle during the past year; "Paid": the individual is engaged in paid work; "Well off": the household is financially comfortable; "Alcohol": the individual has a drinking habit; "Smoking": the individual has a smoking habit; "Health 1 (or 2)": the subjective health status is "very" (or "fairly") healthy; "Exercise1": the individual engages in light exercise at least once per week; "Exercise2": the individual engages in moderate or vigorous exercise at least once per week; "Y 2024": that the survey year is 2024. "Disease1" through "Disease6" are indicators for diseases under treatment or with lasting effects: "Disease1" is hypertension, "Disease2" is diabetes, "Disease3" is hyperlipidemia, "Disease4" is heat disease, "Disease 5" is kindey disease,, and "Disease 6" is other diseases. "ApoE4 hetero" and "ApoE4 homo" are dummy variables indicating carriers of the ApoE4 heterozygous and homozygous genotypes, respectively.
